# A urea-modified tryptophan based *in situ* reducing and stabilizing agent for the fabrication of gold nanoparticles as a Suzuki–Miyaura cross-coupling catalyst in water†

**DOI:** 10.1039/c8na00273h

**Published:** 2019-01-07

**Authors:** Supriya Sasmal, Mintu Debnath, Sujay Kumar Nandi, Debasish Haldar

**Affiliations:** Department of Chemical Sciences, Indian Institute of Science Education and Research Kolkata Mohanpur 741246 West Bengal India deba_h76@iiserkol.ac.in deba_h76@yahoo.com

## Abstract

Urea-modified tryptophan has been used as an *in situ* reducing and stabilizing agent for the fabrication of gold nanoparticles in water. The tryptophan side chain NH has been used for the reduction of gold ions in HAuCl_4_ to metallic gold and carboxylic acid functionality helps to stabilize the gold nanoparticles. This was confirmed by a controlled reaction with urea-modified leucine which failed to form any gold nanoparticles. The resultant gold nanoparticles have been characterized by various spectroscopic techniques such as UV-visible spectroscopy, FT-IR spectroscopy and microscopic techniques such as FE-SEM and TEM. Moreover, we have shown that the urea-modified tryptophan stabilized gold nanoparticles catalyze the Suzuki–Miyaura cross-coupling reaction. The gold nanoparticle catalyzed Suzuki–Miyaura cross-coupling reaction between 4-bromobenzoic acid and phenylboronic acid in water provides 92% yield in 40 minutes. The high efficiency exhibited by the gold nanoparticle catalyst was effectively translated to a large number of Suzuki–Miyaura reactions between halides with phenylboronic acid. The results may inspire further research on gold nanoparticles catalysis in water.

## Abstract 2

Urea modified tryptophan has been used as an *in situ* reducing and stabilizing agent for the fabrication of gold nanoparticles and the gold nanoparticles efficiently catalyzed the Suzuki–Miyaura cross-coupling reaction in water.

## Graphical abstract

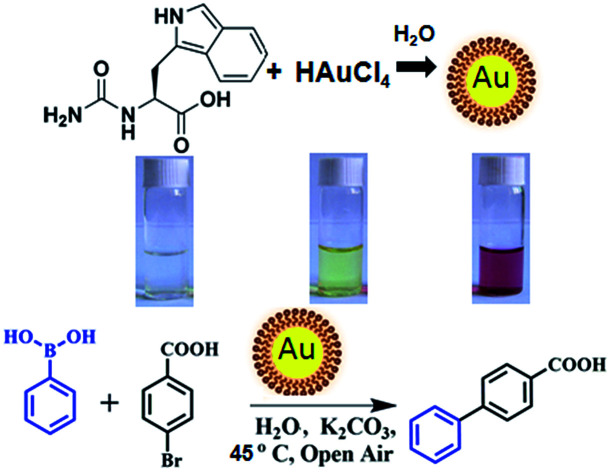

## 1. Introduction

The fabrication of gold nanoparticles of diverse shape, and size, and stabilization of their colloidal suspension is very important due to their potential application as sensors,^1–3^ medicines^4–6^ and catalysts.^7,8^ Gold nanoparticles also exhibit significant optoelectronic properties.^9^ Hence they are promising optical probes because of their colour change upon variation of shape and size. Gold nanoparticles are also useful for biological applications. Most of the reported gold nanoparticles have been prepared by reduction of HAuCl_4_ using borohydride/citrate and stabilization with efficient ligands.^10^ But in this method, there is always a possibility of nanoparticle aggregation.^11^ Hence an *in situ* reduction and stabilization technique is required to avoid nanoparticle aggregation. Recently, there have been few reports for the fabrication of gold nanoparticles with amine functionalities as a reducing agent at higher temperatures.^12–14^ In this regard use of tryptophan or tyrosine is very important. It has been reported that tryptophan^15^ or tyrosine can activate electron or hydrogen transfer in biochemical processes by radical formation.^16–23^ We have utilized this concept for transporting electrons to gold ions in HAuCl_4_ to form metallic gold and finally the gold nanoparticles.

Cross-coupling reactions are very important in synthetic organic chemistry for carbon–carbon bond formation.^24–27^ Among various reported cross-coupling reactions, Suzuki–Miyaura cross-coupling for C–C bond formation has great significance for biphenyl derivative syntheses.^28^ Suzuki–Miyaura coupling reactions generally occur under homogeneous conditions in the presence of a phosphine ligand and a palladium catalyst. These reaction conditions exhibit high activity as well as good selectivity.^29^ But, the costly reagents, long reaction time and high temperature are key issues for industrial applications.

**Fig. 1:**
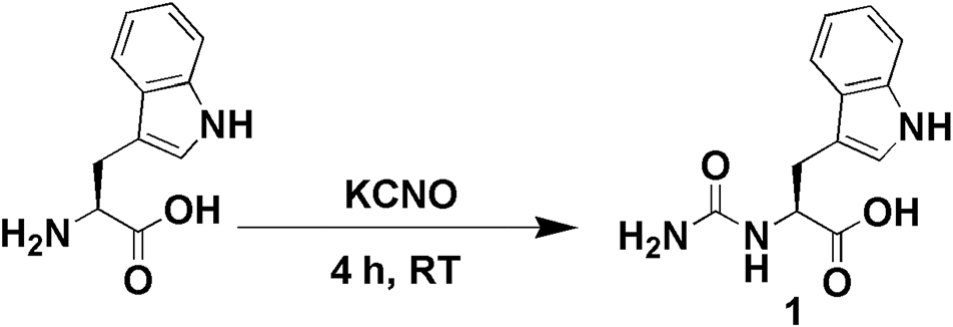
Schematic representation of synthesis of urea-modified tryptophan.

Saikia and co-workers have developed a low-cost method for Suzuki–Miyaura cross-coupling between aryl halides and aryl boronic acids in an isopropanol–water mixture at room temperature. They have used a mixture of Pd(OAc)_2_ and urea as a catalyst for the reaction which shows high conversion even at low catalytic loads.^30^ Guo and co-workers have reported the facile fabrication of very stable gold nanoparticles and used them as an efficient catalyst for Suzuki–Miyaura cross-coupling in an aqueous medium.^31^ With this background, we have fused urea and tryptophan to serve as an *in situ* reducing agent for HAuCl_4_ and Suzuki–Miyaura cross-coupling catalyst preparation. In a recent review, Echavarren and co-workers have raised a question whether gold can act as a catalyst for various cross-coupling reactions as a substitute of palladium.^32^ Combining all previous information, we have planned to develop stable gold nanoparticles for the catalysis of cross-coupling reactions under aerobic conditions. Herein, we have designed a new urea-modified tryptophan (Fig. 1) and used this compound to fabricate gold nanoparticles from the respective gold salts at room temperature in water. The synthesis of the gold nanoparticles was investigated by using various spectroscopic methods like UV-visible spectroscopy and FT-IR spectroscopy and microscopic techniques such as FE-SEM and TEM. The gold nanoparticles act as excellent catalysts for Suzuki–Miyaura cross-coupling between 4-bromobenzoic acid and phenylboronic acid at 45 °C in water.

## 2. Materials and methods

### 2.1. Materials and reagents

All amino acids and chemicals were purchased from Sigma-Aldrich chemicals.

#### Synthesis of urea-modified tryptophan 1

l-Tryptophan (200 mg, 1 mmol) was dissolved in hot H_2_O. Then it was cooled to 5 °C and KCNO (500 mg, 6 mmol) was added to it slowly. After complete addition, it was stirred for another 4 hours at room temperature. Then it was cooled to 5 °C and acidified to pH = 1. A white precipitation was observed. The precipitate was filtered and dried in a vacuum desiccator. A light pink solid was obtained, 0.220 g, yield = 90%.

^1^HNMR (400 MHz, DMSO-d_6_, *δ* in ppm): 10.84 (s, 1H, –ArNH), 7.49 (d, 1H, –ArNH, *J* = 7.63), 7.32 (d, 1H, –ArH, *J* = 8.39), 7.09 (d, 2H, –ArH, *J* = 7.59), 6.95 (d, 1H, –ArH, *J* = 7.63), 6.09 (d, 1H, –NH, *J* = 8.39), 5.59 (s, 2H, –NH_2_), 4.36 (m, 1H, –CH), 3.07 (m, 2H, –CH_2_); ^13^CNMR (100 MHz, DMSO-d_6_, *δ* in ppm): 174.29, 163.27, 136.07, 127.60, 124.20, 123.71, 120.97, 118.3327, 111.32, 109.58, 106.55, 53.0876, 27.73. FT-IR (KBr): 3430, 3388, 3221, 1709, 1651, 1540, 1241 and 740 cm^−1^. ESI-MS: *m*/*z* 175.1202 [M + H]^+^; *M*_calcd_: 175.1224, 213.0808 [M + K]^+^; *M*_calcd_: 213.1002, 349.2479 [2 M + H]^+^; *M*_calcd_: 349.2411.

#### Synthesis of urea-modified leucine 2

l-Leucine (130 mg, 1 mmol) was dissolved in hot H_2_O. Then it was cooled to 5 °C and KCNO (500 mg, 6 mmol) was added to it slowly. After complete addition, it was stirred for another 4 hours. Then it was cooled to 5 °C and acidified to pH = 1. A white precipitate was observed. The precipitate was filtered and dried in a vacuum desiccator. A white solid was obtained, 0.166 g, yield = 95%.

^1^HNMR (400 MHz, DMSO-d_6_, *δ* in ppm): 6.19 (d, 1H, –NH, *J* = 6.8), 5.54 (s, 2H, –NH_2_), 4.05 (t, 1H, –CαH, *J* = 4.04), 1.44–1.4 (m, 2H, –CH_2_), 0.87 (m, 6H, –CH_3_); ^13^CNMR (100 MHz, DMSO-d_6_, *δ* in ppm): 172.46, 162.77, 47.93, 38.17, 21.39, 19.93, 18.69. FT-IR (KBr): 3460, 3293, 2959, 1691, 1640, 1571, 1311,1017 and 719 cm^−1^, ESI-MS: *m*/*z* 247.527 [M]^+^; *M*_calcd_: 247.4987.

#### Suzuki–Miyaura cross-coupling reaction

0.2 g (1 mmol) of 4-bromobenzoic acid, 0.14 g (1.2 mmol) of phenylboronic acid and 0.7 g (5 mmol) of K_2_CO_3_ were taken in 15 mL water. To this mixture, compound 1-gold nanoparticles was added and heated at 45 °C for 45 minutes. The clear solution was acidified with 2 N HCl. The white precipitate was filtered, washed with water and dried under vacuum. A white solid was obtained, 198 mg, yield = 98.37%.

^1^H NMR (400 MHz, DMSO-d_6_. *δ* ppm, 298 K): 12.93 [s, 1H, –CO_2_H], 8.02 [d, 2H, *J* = 8.54, –Ar^1^H], 7.79 [d, 2H, *J* = 7.93, –Ar^1^H], 7.72 [d, 2H, *J* = 7.93, –Ar^2^H], 7.49 [d, 2H, *J* = 7.32, –Ar^2^H], 7.41 [t, 1H, *J* = 7.32, –Ar^2^H]; ^13^C NMR (100 MHz, DMSO-d_6_. *δ* ppm, 298 K): 144.33, 139.05, 130.01, 129.67, 129.32, 126.99, 126.85; FT-IR (KBr): 2984, 2840, 2667, 2552, 1682, 1608, 1423, 1292, 936, 863, 752, 698 cm^−1^; ESI-MS: *m*/*z* 236.86 [M + K]^+^; *M*_calcd_: 198.22.

### 2.2. NMR experiments

All NMR experiments were performed on a Jeol 400 MHz spectrometer at 278 K. Compound concentrations were in the range of 1–10 mM in DMSO-d_6_.

### 2.3. FT-IR spectroscopy

Solid-state FT-IR spectra following the KBr disk technique were measured using a Perkin Elmer Spectrum RX1 spectrophotometer.

### 2.4. Mass spectrometry

Mass spectra of the compounds were recorded on a Q-Tof Micro YA263 high-resolution (Waters Corporation) mass spectrometer by positive-mode electrospray ionization.

### 2.5. Single crystal X-ray diffraction study

Single crystal X-ray analysis of compound 2 was performed on a Bruker high-resolution X-ray diffractometer instrument with MoKα radiation. Data were processed using the Bruker SAINT package and the structure solution and refinement procedures were performed using SHELX97. CCDC 1871263 contains the supplementary crystallographic data for compound 2.

### 2.6. UV/vis spectroscopy

The absorption spectra of the samples were recorded on a Perkin Elmer UV-vis spectrophotometer.

### 2.7. Field emission scanning electron microscopy

The morphologies of the reported compound 1-gold nanoparticle conjugate were investigated using field emission-scanning electron microscopy (FE-SEM). For FE-SEM, a small amount of suspension of the conjugate was drop cast on a clean glass coverslip and dried by slow evaporation at room temperature. Finally, the sample was dried under reduced pressure for two days at 30 °C. The samples were gold-coated, and the micrographs were captured using a Jeol Scanning Microscope-JSM-6700F.

### 2.8. Transmission electron microscopy

The TEM experiments were performed using a small amount of the aqueous solution of the gold nanoparticles on a carbon-coated copper grid (300 mesh) by slow evaporation and allowed to dry under vacuum at 30 °C for 24 h. Images were taken in both the transmission mode and diffraction mode. TEM was done by using a JEOL JEM 2010 electron microscope.

## 3. Results and discussion

The synthesis of the urea-modified tryptophan 1 was a relatively simple one pot reaction. l-Tryptophan was allowed to react with KCNO in an aqueous medium to produce the urea-modified tryptophan (Fig. 1). The logic behind the design of the compound was to study the effect of urea substitution in l-tryptophan NH_2_ and to study its properties. The urea will dictate the intermolecular hydrogen bonding and the tryptophan moiety will act as a reducing agent. We have also developed a negative control as urea-modified leucine 2, using the same synthetic strategy. The synthesized compounds were purified by column chromatography and characterized by ^1^H-NMR (nuclear magnetic resonance), ^13^C-NMR, Fourier-transform infrared (FT-IR) analysis (see the ESI†) and mass spectrometry.

**Fig. 2:**
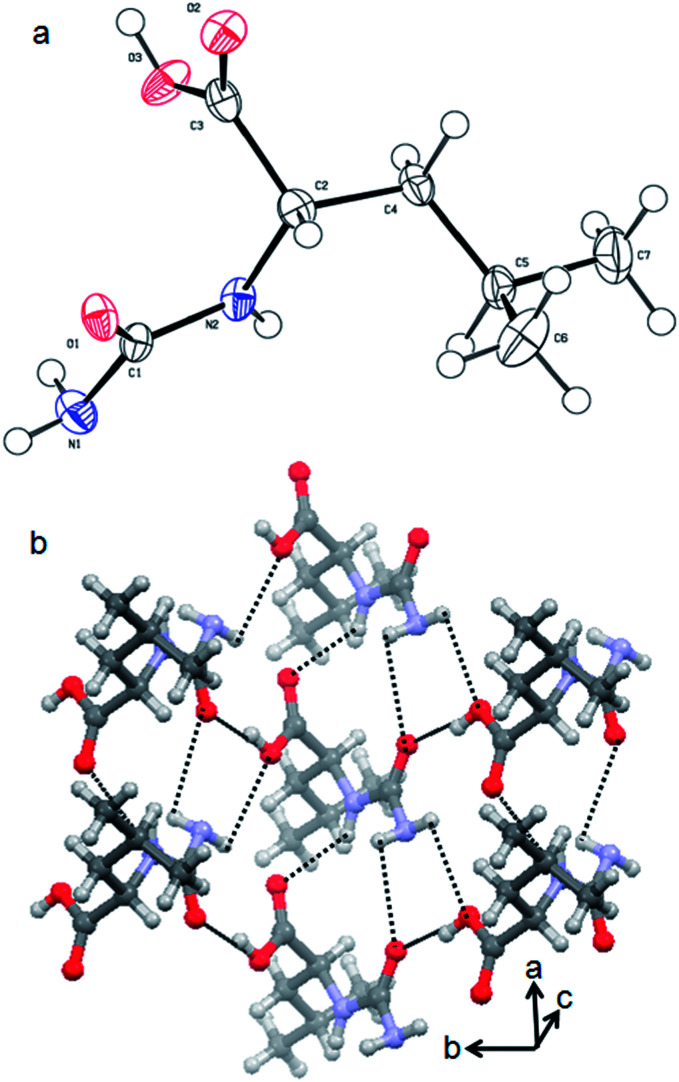
(a) ORTEP diagram of urea-modified leucine 2. (b) The packing diagram of urea-modified leucine 2. Hydrogen bonds are shown as dotted lines.

The urea-modified leucine 2 was also characterized by single crystal X-ray diffraction analysis. Colourless crystals of compound 2 suitable for X-ray crystallography were obtained from aqueous solutions by slow evaporation. The asymmetric unit contains one molecule of compound 2. The ORTEP diagram of compound 2 with the atomic numbering scheme is depicted in Fig. 2a. The packing diagram shows that each compound 2 molecule is interacting with six molecules through N–H⋯O and O–H⋯O hydrogen bonds and thus forms a hexagonal structure along crystallographic *a* and *b* directions. The hydrogen bonding parameters are listed in ESI Table 1.†

Previous reports showed that *in situ* gold nanoparticle synthesis can be achieved using tryptophan or tyrosine-containing proteins or peptides due to their significant electron-donating functionality. Tryptophan or tyrosine residues have been used to reduce Au^III^ ions into gold atoms.^29^ So, by modifying the redox active tryptophan residue with urea, we wish to develop a method for *in situ* gold nanoparticle synthesis. The following protocol has been used for the fabrication of gold nanoparticles with compound 1. 1 mg of urea-modified tryptophan 1 was dissolved in 5 mL water and 0.5 mL HAuCl_4_ solution (20 mg in 2 mL water) was added and was stirred to produce a homogeneous solution. The yellow colour solution became colourless [Au^III^ to Au^I^]. Finally, a violet coloured solution was formed within a couple of minutes which indicates the formation and presence of gold nanoparticles in solution [Au^0^] (ESI Fig. S1†). The gold nanoparticles have been formed due to the reduction of Au^III^ ions in HAuCl_4_ to gold atoms *via* the oxidation of the indole NH of tryptophan residue (ESI Fig. S2†). As per our assumption, the urea-modified leucine 2 has failed to form gold nanoparticles under the same conditions. This confirms that the urea NH is not acting as an *in situ* reducing agent.

**Fig. 3:**
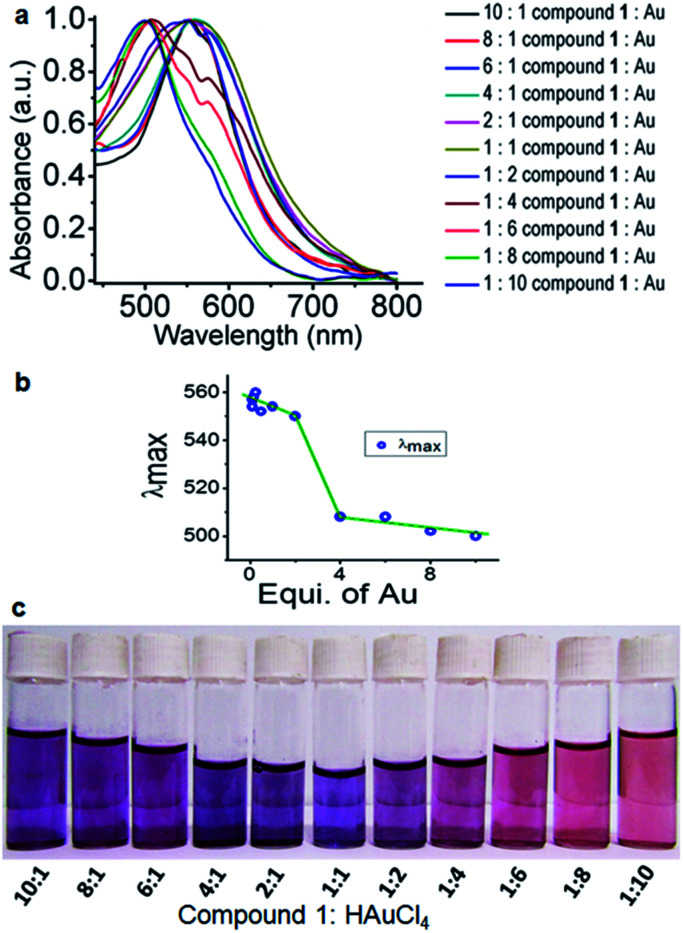
(a) Absorption spectra of compound 1 stabilized gold nanoparticles at different ratios of compound 1 and HAuCl_4_. (b) Plot of *λ*_max_ for gold nanoparticle sizes with variation of concentrations of compound 1 and HAuCl_4_. (c) Naked eye visualization of gold nanoparticles in water with variation of concentrations of compound 1 and HAuCl_4_.

The formation of gold nanoparticles using compound 1 was characterized by UV-visible spectroscopy of the solution. Fig. 3a exhibits the UV-visible spectrum of the compound-1-gold nanoparticle solution. From absorption spectra, the band at 545 nm is characteristic of colloidal gold nanoparticles having a size range of 15 nm for compound 1 : HAuCl_4_ 10 : 1.^27^ The gold nanoparticles are stable at room temperature for around three months showing no deposition. However, different sizes of gold nanoparticles are formed by varying the ratio of HAuCl_4_ and compound 1 which is confirmed by UV-visible spectra (Fig. 3a). Thus, for compound 1 : HAuCl_4_ 1 : 10 the absorption band blue shifts 40 nm and appeared at 505 nm (ESI Fig. S3†). The change of sizes of gold nanoparticles with the variation of concentrations is depicted in Fig. 3b. This phenomenon of variation of gold nanoparticle sizes at a different ratio of compound 1 and HAuCl_4_ can be visualized by the naked eye (Fig. 3c).

**Fig. 4:**
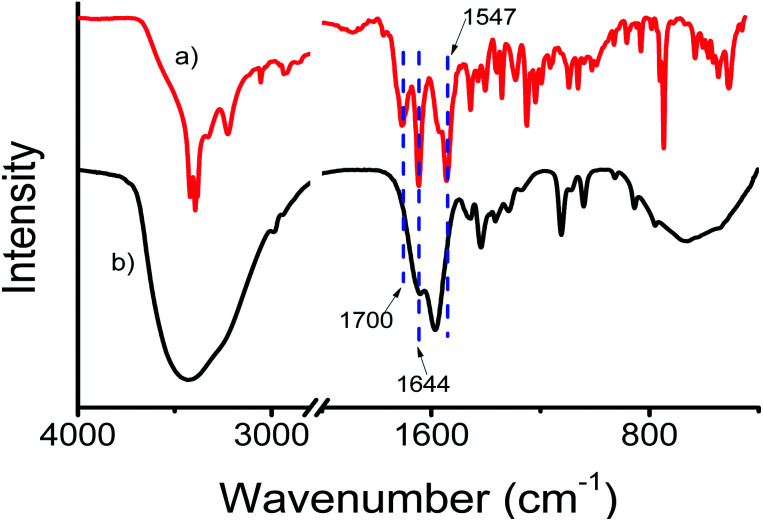
The FT-IR spectra (solid state) of (a) compound 1 and (b) compound 1 stabilized gold nanoparticles.

The presence of the urea-modified tryptophan 1 on the gold nanoparticle surface has been studied by FT-IR spectroscopy. The FT-IR spectrum of only urea-modified tryptophan 1 exhibits amide I and amide II bands at 1644 and 1547 cm^−1^ respectively (Fig. 4a). The band attributed to acid carbonyl vibration appeared at 1700 cm^−1^. Fig. 4b presents the spectrum of compound-1-gold nanoparticles. The spectrum exhibits bands at 1644 and at 1584 cm^−1^ attributed to amides I and II of compound 1. But the band attribute to acid function becomes broad, which indicates that the gold nanoparticles have been stabilized by the acid functional groups. So, the existence of the corresponding bands in FT-IR establishes the stabilization of gold nanoparticles by compound 1.

**Fig. 5:**
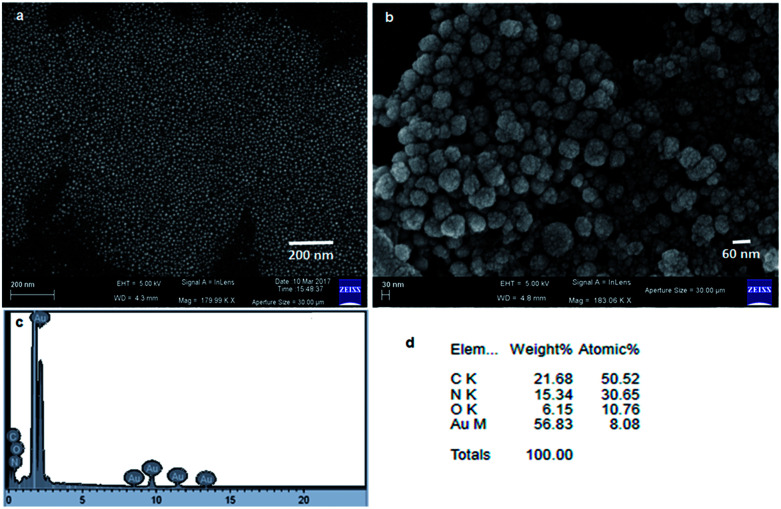
(a) and (b) FE-SEM images showing the spherical morphology of gold nanoparticles. (c) EDX shows the presence of gold. (d) Weight% of elements from EDX.

Furthermore, to characterize the gold nanoparticles, field-emission scanning electron microscopy (FE-SEM) has been used. For FE-SEM, a drop of gold nanoparticle violet solution (compound 1 : HAuCl_4_ 10 : 1) in water was drop cast on a microscopic glass cover slip and was dried under reduced pressure for two days at room temperature. Fig. 5a and b depict the images of spherical gold nanoparticles. The average diameters of the nanoparticles are in the range of 30 nm to 45 nm. Fig. 5c and d show the EDX pattern of gold nanoparticles and weight% of elements from EDX.

**Fig. 6:**
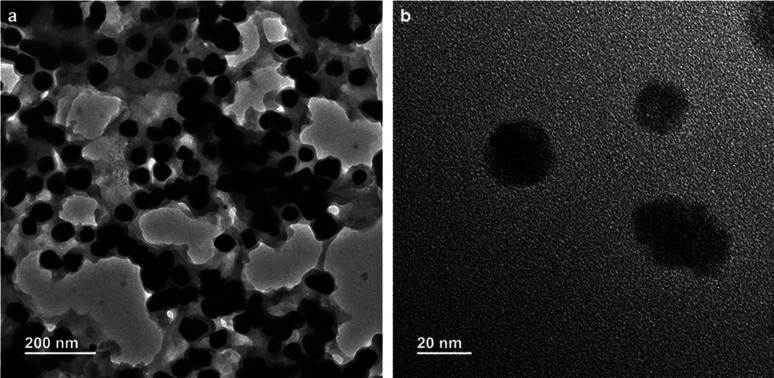
(a) TEM images showing the spherical morphology of gold nanoparticles. (b) TEM image of gold nanoparticles at high magnification.

The nanocomposite was also characterized by transmission electron microscopy (TEM). A freshly prepared violet suspension of gold nanoparticles (compound 1 : HAuCl_4_ 10 : 1) in water was drop cast on a carbon coated copper grid for TEM. The grid was dried under vacuum at 30 °C for three days. The transmission electron microscopy (TEM) images (Fig. 6a and b) of the compound 1 stabilized gold nanoparticles confirm the spherical shape of the nanoparticles. The average diameters of the compound 1 gold nanoparticles were calculated to be between 30 and 45 nm. These results exhibit that nanoparticles of nearly the same size have been obtained in a particular reagent ratio.

**Fig. 7:**
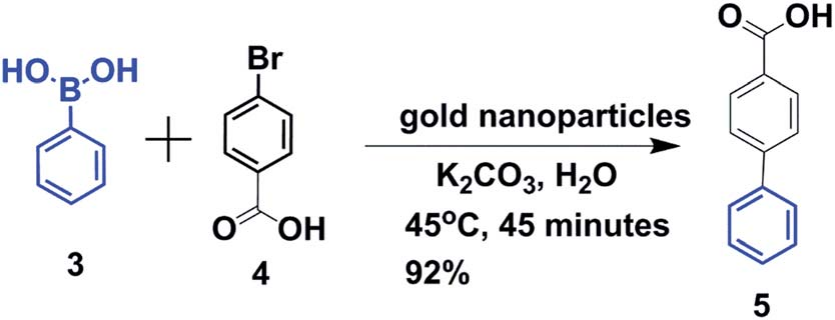
Schematic presentation of Suzuki–Miyaura cross-coupling of *p*-bromobenzoic acid and phenylboronic acid in water.

Recently, the catalytic behavior of gold nanoparticles in various organic syntheses has attracted significant attention.^36–38^ But, to the best of our knowledge, the use of gold nanoparticles as a catalyst for Suzuki–Miyaura cross-coupling in water is relatively rare.^31–35^ Herein, we show that gold nanoparticles are very good catalysts for Suzuki–Miyaura cross-coupling between aryl halides and aryl-boronic acids in water at comparatively low temperature. To investigate the catalytic activity of urea-modified tryptophan stabilized gold nanoparticles and to determine the optimal conditions for C–C bond formation, 4-bromobenzoic acid and phenylboronic acid were chosen as model substrates. Water was chosen as the reaction medium due to the low cost as well as the green-chemistry approach.^39,40^ 0.2 g (1 mmol) of 4-bromobenzoic acid, 0.14 g (1.2 mmol) of phenylboronic acid and 0.7 g (5 mmol) of K_2_CO_3_ were taken in 15 mL water (Fig. 7). To this solution, urea-modified tryptophan stabilized gold nanoparticles were added and the solution was heated at 45 °C for 45 minutes. Finally, the clear solution was centrifuged and decanted off and acidified with 2 N HCl. A white precipitate of the product biphenyl carboxylic acid appeared (Fig. 7). A proposed mechanism for gold nanoparticle catalyzed Suzuki–Miyaura reactions is depicted in ESI Fig. S4.†^27^

**Fig. 8:**
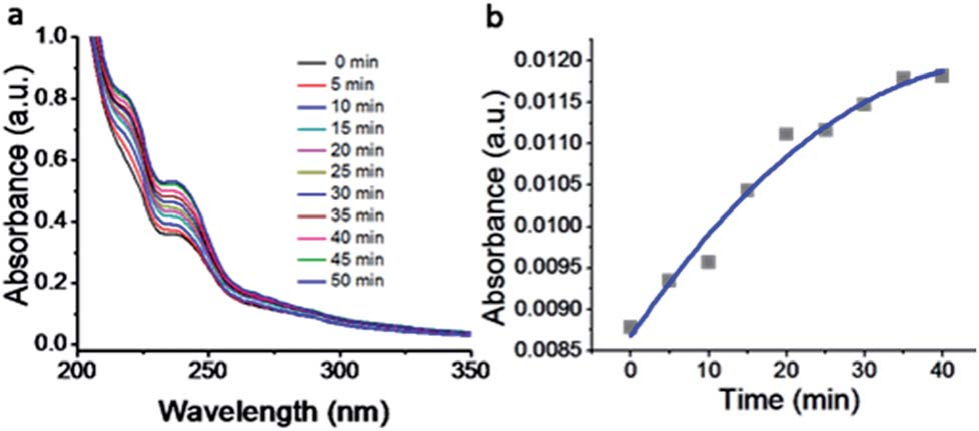
(a) UV-visible kinetic study of the Suzuki–Miyaura cross-coupling reaction catalyzed by compound 1-gold nanoparticles. (b) Plot of intensity with increasing time.

The kinetics of the gold nanoparticle catalyzed Suzuki–Miyaura C–C bond formation were studied by UV-visible spectroscopy by placing the reaction mixture directly in a 1.0 cm path length quartz cuvette. The spectrum in Fig. 7a shows the appearance of a new band at 220 nm. Finally, the intensity of this band increases with increasing time (Fig. 8a). The plot of kinetics shows that initially the Suzuki–Miyaura cross-coupling reaction rate increases and after 40 minutes it becomes saturated (Fig. 8b). The product was filtered, washed with water and dried under vacuum (92% yield in 45 minutes). The product was characterized by ^1^H-NMR, ^13^C-NMR and FT-IR spectroscopic analysis (see the ESI†). Repeating this experiment confirmed the reproducibility of the results. Moreover, when the gold nanoparticle catalysts were used after ageing for three months, almost the same yield was obtained. Moreover, with these optimized reaction conditions, we have explored the scope of gold nanoparticle catalyzed Suzuki–Miyaura C–C bond formation reactions (Table 1). The high efficiency exhibited by the gold nanoparticle catalyst in the model Suzuki–Miyaura C–C bond formation reaction was effectively translated to a large number of aryl halides with phenylboronic acid. 4-Chloro benzoic acid and 4-chloro benzaldehyde provided 78% and 88% yield, respectively, whereas 2-chloro benzaldehyde provided 76% yield. 2-Chlorobenzaldehyde affords a lower yield in Suzuki–Miyaura coupling due to steric effects. Due to the steric and electronic effects of the substituent on the aromatic cycle of aryl halides, 4-chlorobenzoic acid and 4-chlorobenzaldehyde provided higher yields than 2-chlorobenzaldehyde. The reactions of 1-chloro-4-vinylbenzene with phenylboronic acid were sluggish, and the products were obtained in 75% yield after 45 minutes.

**Table 1:** Suzuki–Miyaura reaction of aryl halides with phenylboronic acid

Reactant	Product	Reaction temp. (°C)	Reaction time (min)	Yield^a^ (%)
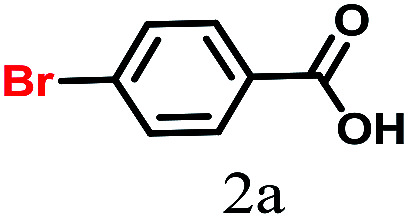	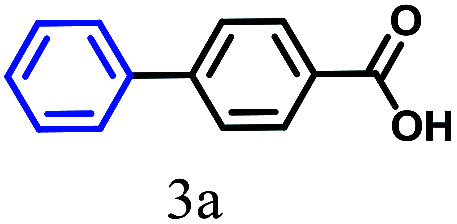	40	45	98.37
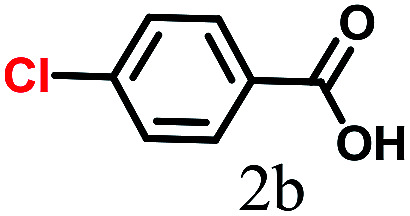	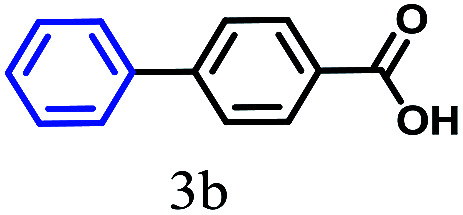	40	50	78
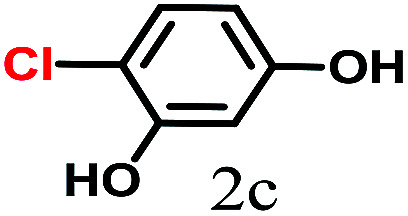	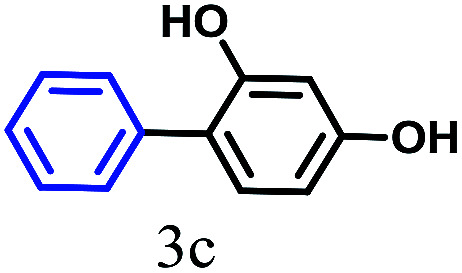	40	45	89.64
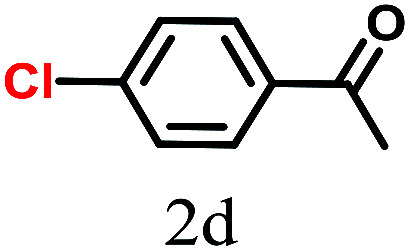	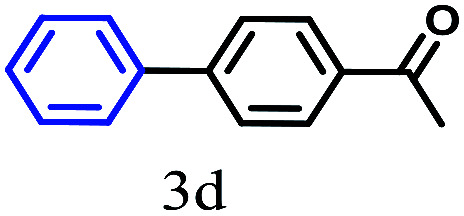	50	50	88
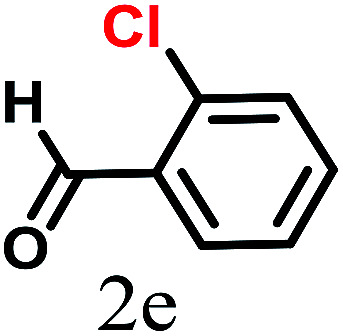	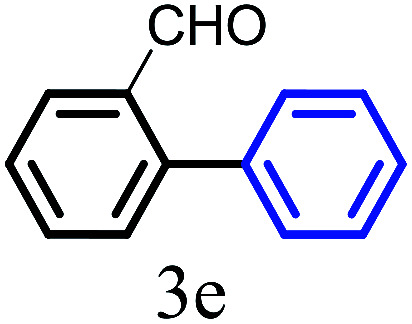	40	50	76
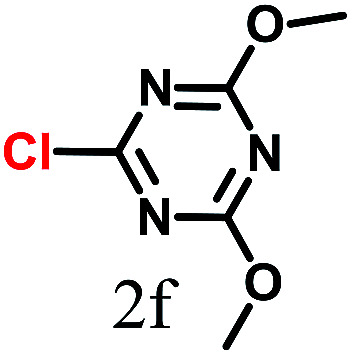	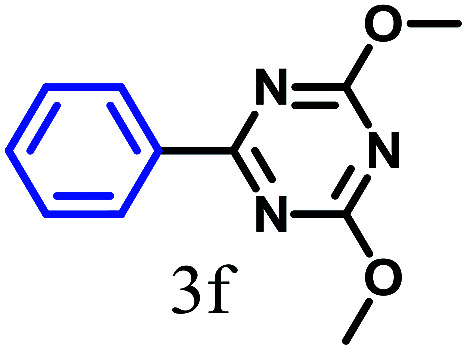	50	50	97
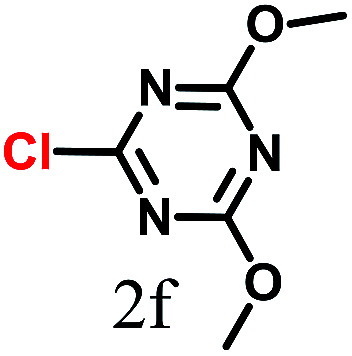	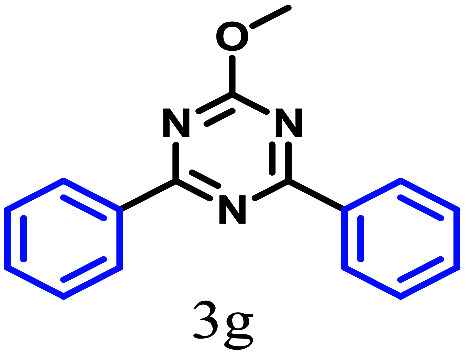	60	45	95
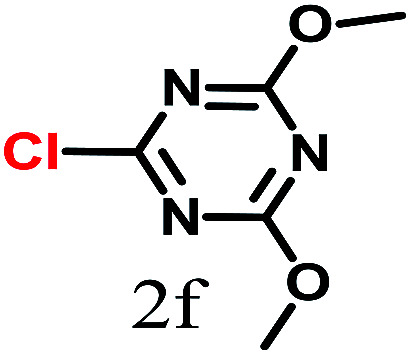	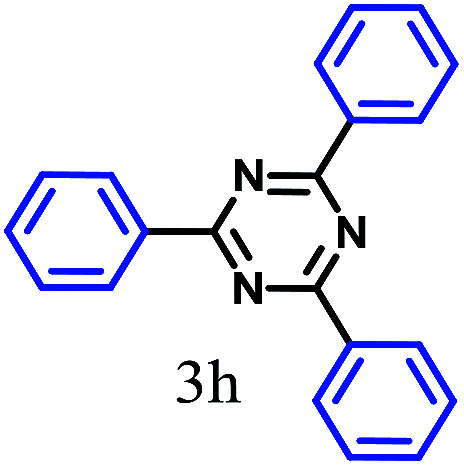	80	50	94
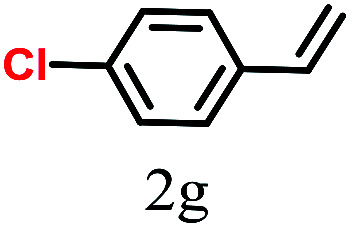	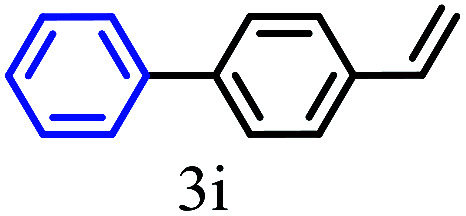	40	45	75
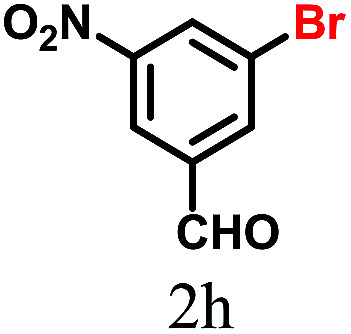	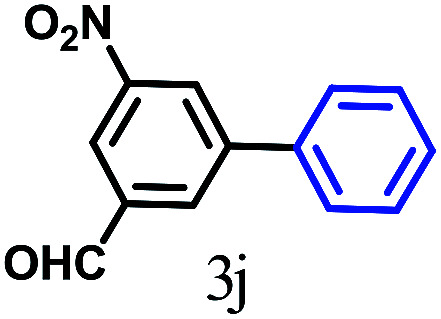	50	50	81
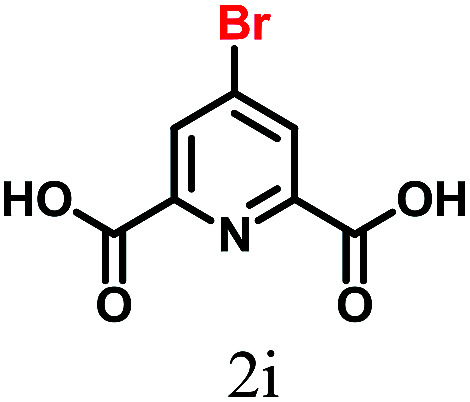	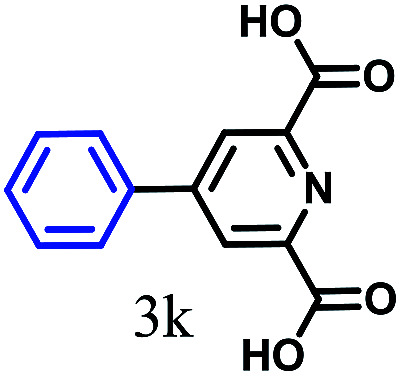	50	45	78

a Isolated yield.

**Fig. 9:**
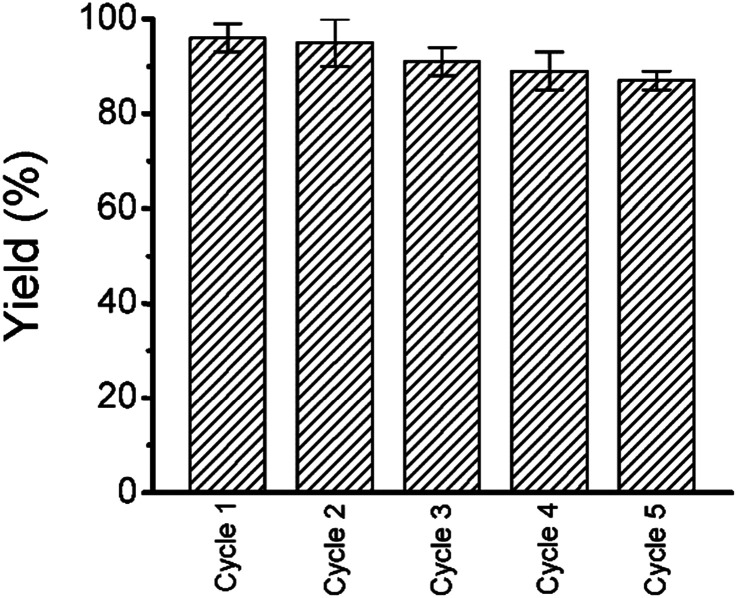
The reproducible nature of the catalyst up to 5 cycle with 90% yield.

To reuse the catalyst we have collected the gold nanoparticles by centrifugation. Simple drying is enough for the reuse of the gold nanoparticles. We checked the reusability; the Suzuki–Miyaura cross coupling between 4-bromobenzoic acid and phenylboronic acid was determined for five consecutive cycles with the recovered gold nanoparticles (Fig. 9).

**Fig. 10:**
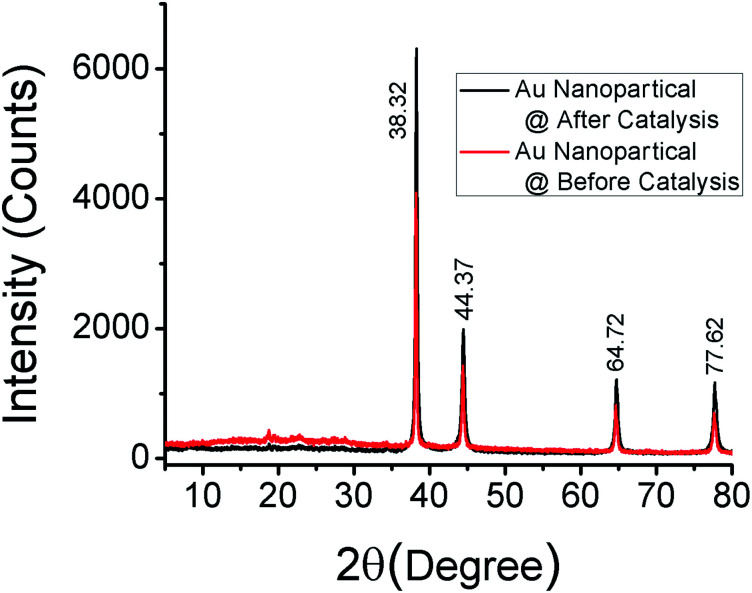
The XRD patterns of the as-synthesized gold nanoparticles and recovered gold nanoparticles after the Suzuki–Miyaura C–C bond formation reaction.

To characterize the recovered catalyst and compare with the fresh catalyst and to determine whether there have been any changes of the crystalline structure of gold nanoparticles, XRD analysis has been performed. Fig. 10 shows that there is no change of the crystalline structure of gold nanoparticles after the Suzuki–Miyaura C–C bond formation reaction.

**Fig. 11:**
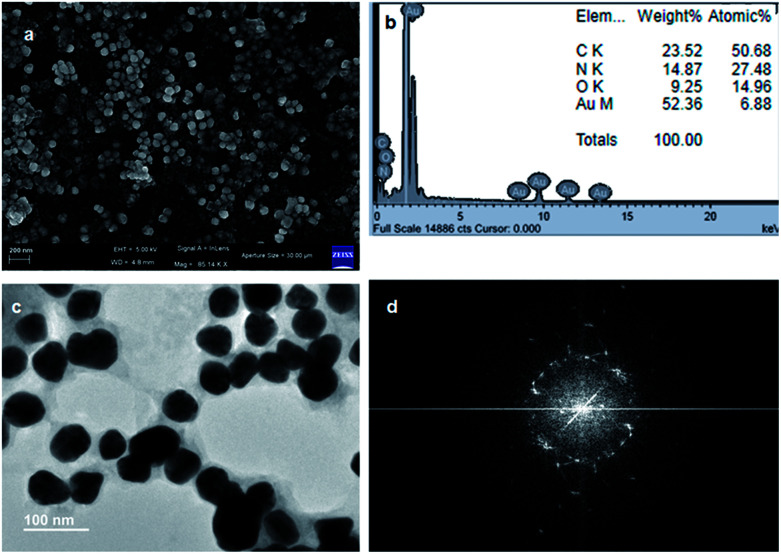
(a) FE-SEM image showing the spherical morphology of the recovered gold nanoparticles from the Suzuki–Miyaura C–C bond formation reaction. (b) EDX showing the presence of gold nanoparticles (inset: weight% of elements from EDX). (c) TEM image showing the spherical morphology of the recovered gold nanoparticles from the Suzuki–Miyaura C–C bond formation reaction. (d) The electron diffraction pattern of the recovered gold nanoparticles.

Moreover, we have used SEM and TEM to characterize the recovered catalyst form the Suzuki–Miyaura C–C bond formation reaction and compared with the fresh catalyst to determine whether there have been any changes of the morphology and aggregations of gold nanoparticles. The FESEM image (Fig. 11a) depicts the spherical shape of the recovered gold nanoparticles. Fig. 11b shows the EDX pattern of the gold nanoparticles and weight% of elements from EDX. There is no significant change with reference to those before the Suzuki–Miyaura C–C bond formation reaction. The TEM image (Fig. 11c) shows the spherical shape of the recovered gold nanoparticles from the Suzuki–Miyaura C–C bond formation reaction. The average diameters of the nanoparticles are in the range of 30 nm to 45 nm. Fig. 11d exhibits the electron diffraction pattern of the recovered gold nanoparticles from the Suzuki–Miyaura C–C bond formation reaction.

## 4. Conclusions

In conclusion, we have successfully synthesized urea-modified tryptophan and leucine. Furthermore, we have used the urea-modified tryptophan as an *in situ* reducing agent as well as a stabilizing agent for the fabrication of gold nanoparticles from HAuCl_4_ at room temperature in water. The urea-modified tryptophan gives an electron to a gold ion and forms an intermediate tryptophan radical. The tryptophan radical finally converts to either native tryptophan or ditryptophan. Moreover, the gold nanoparticles act as excellent catalysts for the Suzuki–Miyaura cross-coupling reaction between phenylboronic acid and *p*-bromobenzoic acid at 45 °C in water. The high efficiency exhibited by the gold nanoparticle catalyst in the model Suzuki–Miyaura C–C bond formation reaction was effectively translated to a large number of aryl halides with phenylboronic acid. The results presented in this study may foster research on gold nanoparticle catalyzed C–C bond formation.

## Conflicts of interest

The authors declare no conflict of interest.

## Supplementary Material

NA-001-C8NA00273H-s001

NA-001-C8NA00273H-s002
