# Supplementary material for: A urea-modified tryptophan based in situ reducing and stabilizing agent for the fabrication of gold nanoparticles as a Suzuki–Miyaura cross-coupling catalyst in water
Source: Nanoscale Adv. 2019 Jan 7;1(4):1380–6. doi: 10.1039/c8na00273h (PMC9418380; doi:10.1039/c8na00273h)
Supplement: NA-001-C8NA00273H-s001 [file NA-001-C8NA00273H-s001.pdf]

Electronic Supplementary Material (ESI) for Nanoscale Advances.
This journal is © The Royal Society of Chemistry 2019

Supporting Information,

Urea-modified tryptophan based *in situ* reducing and stabilizing agent for fabrication of gold nanoparticles as Suzuki-Miyaura cross-coupling catalyst in water

Supriya Sasmal, Mintu Debnath, Sujay Kumar Nandi and Debasish Haldar*

Department of Chemical Sciences, Indian Institute of Science Education and Research Kolkata, Mohanpur, West Bengal 741252, India,

Fax: (+)913325873020; Tel: +913325873119;

E-mail: deba_h76@yahoo.com; deba_h76@iiserkol.ac.in

Contain.	Page No.	Contain.	Page No.	Contain.	Page No.
ESI-Figure S1	2	Figure S18	17	Figure S38	27
ESI-Figure S2	2	Figure S19	18	Figure S39	28
ESI-Figure S3	3	Figure S20	18	Figure S40	28
Figure S4	4	Figure S21	19	Figure S41	29
Scheme 1	4	Figure S22	19	Figure S42	29
Figure S5	6	Figure S23	20	Figure S43	30
Figure S6	6	Figure S24	20	Figure S44	30
Figure S7	7	Figure S25	21	Figure S45	31
Figure S8	7	Figure S26	21	Figure S46	31
Figure S9	8	Figure S27	22	Figure S47	32
Figure S10	8	Figure S28	22	Figure S48	32
Figure S11	9	Figure S29	23	Figure S49	33
Figure S12	9	Figure S30	23	Figure S50	33
Scheme 2	10	Figure S31	24	Figure S51	34
Figure S13	15	Figure S32	24	Figure S52	34
Figure S14	15	Figure S33	25	ESI Table 1	35
Figure S15	16	Figure S34	25	Table 2	36
Figure S16	16	Figure S35	26	Table 3	37
Figure S17	17	Figure S36	26	Figure S53	37
Figure S37	27	Figure S37		Figure S54	38

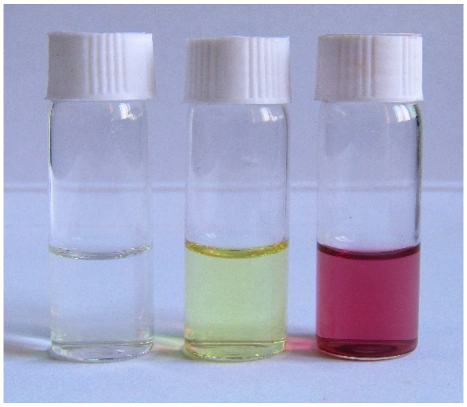

ESI-Figure S1. The water solution of urea modified tryptophan 1, $HAuCl_4$ and gold nanoparticles.

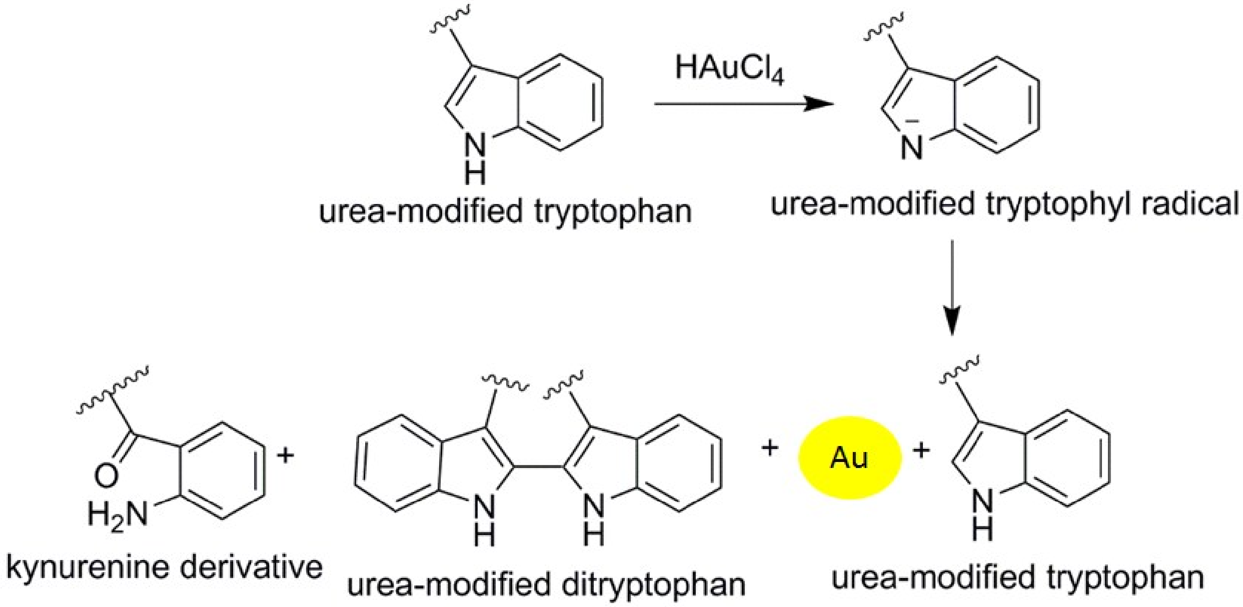

ESI-Figure S2. The proposed mechanism of gold nanoparticle from urea modified tryptophan 1 and $HAuCl_4$.

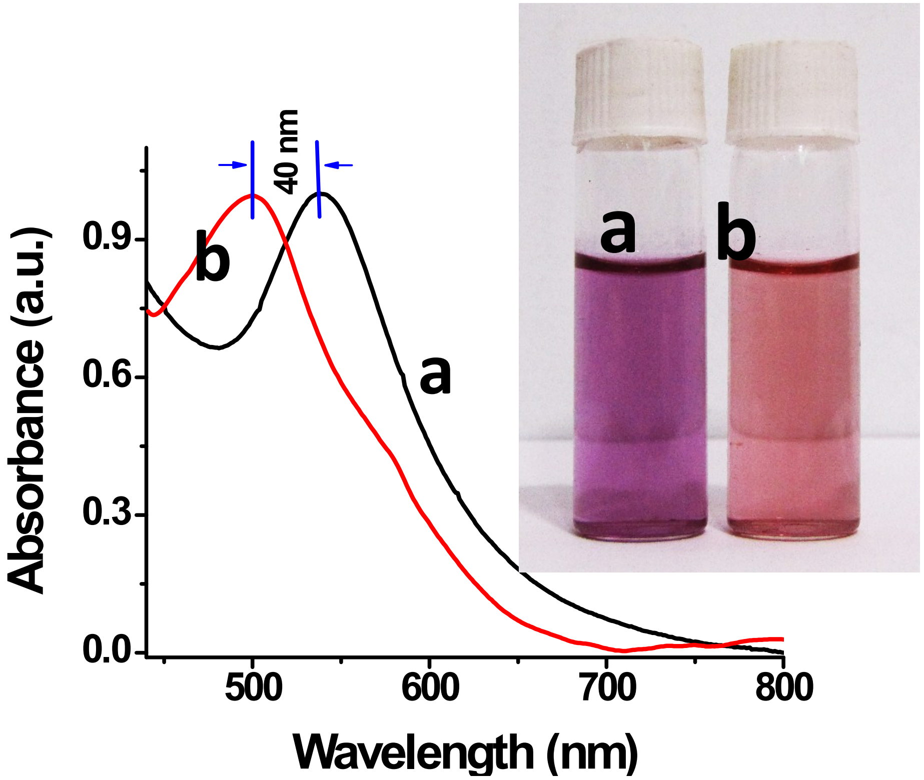

ESI-Figure S3. Absorption spectra of gold nanoparticle a) compound 1:$HAuCl_4$ = 10:1 and b) compound 1:$HAuCl_4$ = 1:10.

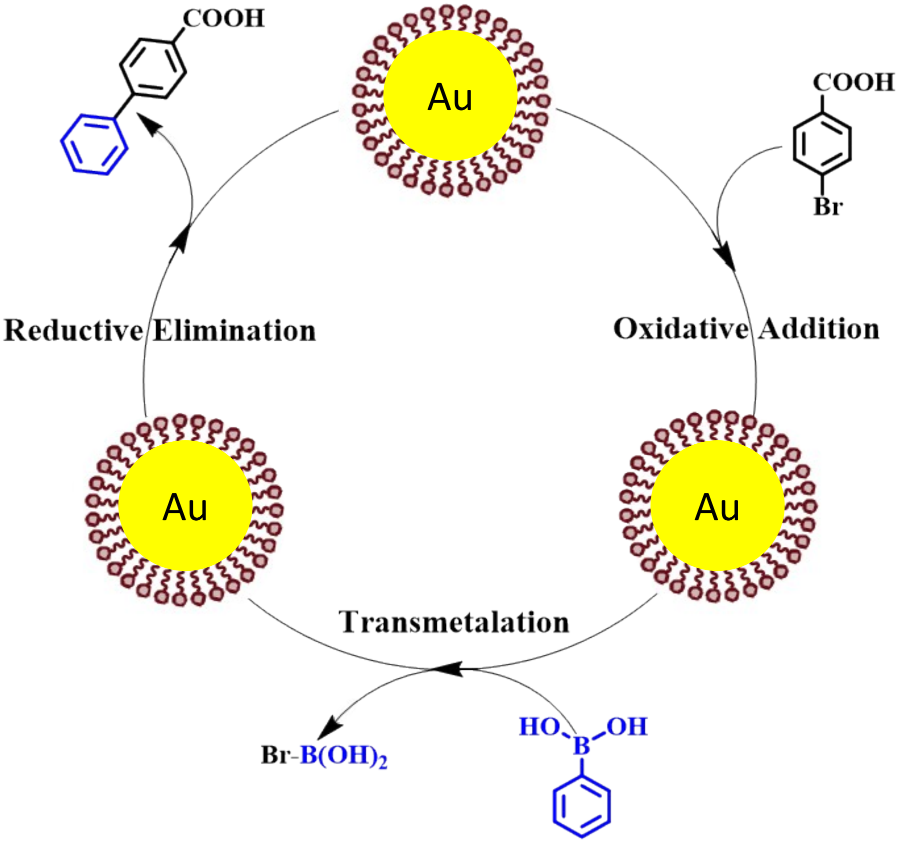

Figure S4. The mechanism of gold nanoparticle catalyzed cross-coupling reaction.

Experimental Section

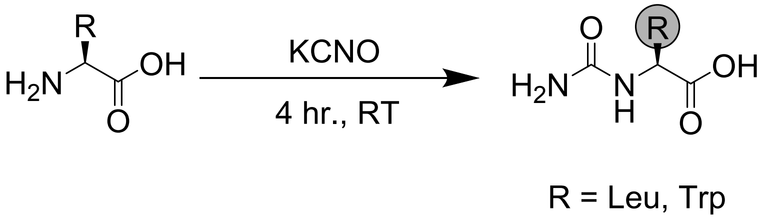

Scheme 1: Synthesis of Urea derivative Amino Acid

Materials: L-Tryptophan, L-Leucine KCNO are purchased from SRL and $HAuCl_4$ are purchased from Sigma-Aldrich.

Synthesis of Urea derivative: L-Amino Acid (1 mmol) was dissolved in hot H_2O. Then cooled it to 5°C and KCNO (6 mmol) was added to it slowly. After complete addition it was stirred for another 4 hours. Then cooled it to 5°C and acidified it to pH = 1. A white precipitation was observed. Filtered the precipitate and dried it in vacuum desiccator.

(a) Urea modified tryptophan 1

^{1}HNMR (400MHz, DMSO-d$_6$, δ in ppm): 10.84 (s, 1H, -ArNH), 7.49 (d, 1H, -ArNH J = 7.63), 7.32(d, 1H, , -ArH, J = 8.39), 7.09 (d, 2H, , -ArH, J = 7.59), 6.95 (d, 1H, , -ArH, J = 7.63), 6.09 (d, 1H, , -NH, J = 8.39), 5.59 (s, 2H, , -NH_2), 4.36 (m, 1H, -CH), 3.07 (m, 2H, -CH_2).;

^{13}CNMR (100MHz, DMSO-d$_6$, δ in ppm): 174.29, 163.27, 136.07, 127.60, 124.20, 123.71, 120.97, 118.3327, 111.32, 109.58, 106.55, 53.0876, 27.73.

FT-IR (KBr): 3430, 3388, 3221, 1709, 1651, 1540, 1241 and 740 cm^{-1}.

ESI-MS: m/z 175.1202 $[M+H]^+$; M_{calcd}:175.1224, 213.0808 $[M+K]^+$; M_{calcd}:213.1002, 349.2479 $[2M+H]^+$; M_{calcd}:349.2411,

(a) Urea modified leucine

^{1}H NMR (400MHz, DMSO-d$_6$, δ in ppm) (Figure 7): 6.19 (d, 1H, -NH, J = 6.8), 5.54 (s, 2H, -NH_2), 4.05 (t, 1H, -C$^\alpha$H, J = 4.04), 1.44 - 1.4 (m, 2H, -CH_2), 0.87 (m, 6H, -CH_3).

^{13}C NMR (100MHz, DMSO-d$_6$, δ in ppm) (Figure 8): 172.46, 162.77, 47.93, 38.17, 21.39, 19.93, 18.69.

FT-IR (KBr): 3460, 3293, 2959, 1691, 1640, 1571, 1311,1017 and719 cm^{-1} ,

ESI-MS: m/z 247.527 $[M]^+$; M_{calcd}: 247.4987,

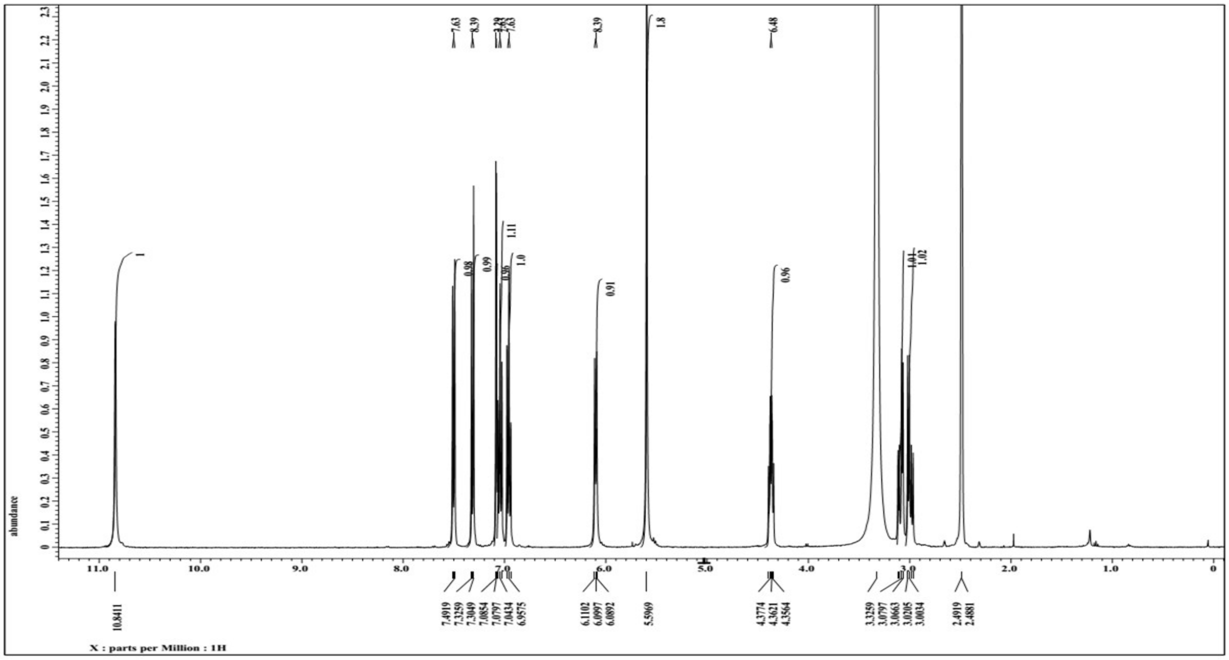

Figure S5: ^{1}HNMR (400 MHz, DMSO-d_6. δ_{ppm}, 298 K) spectra of Compound **1.**

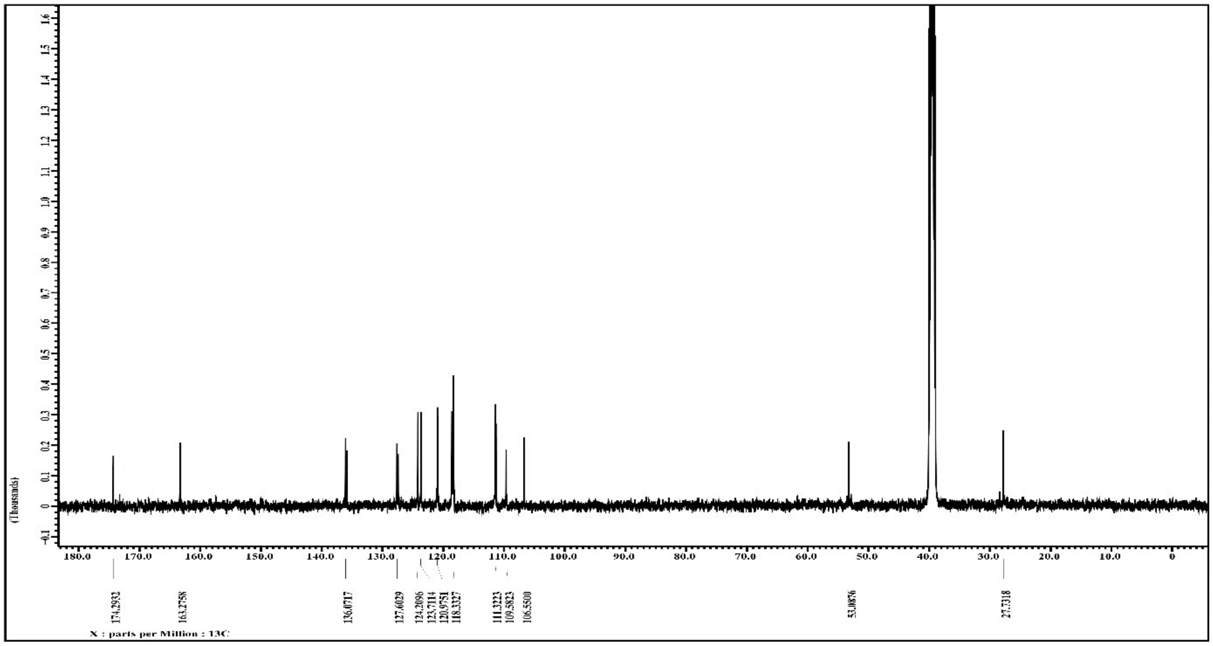

Figure S6: ^{13}CNMR (100 MHz, DMSO-d_6. δ_{ppm}, 298 K) spectra of Compound **1.**

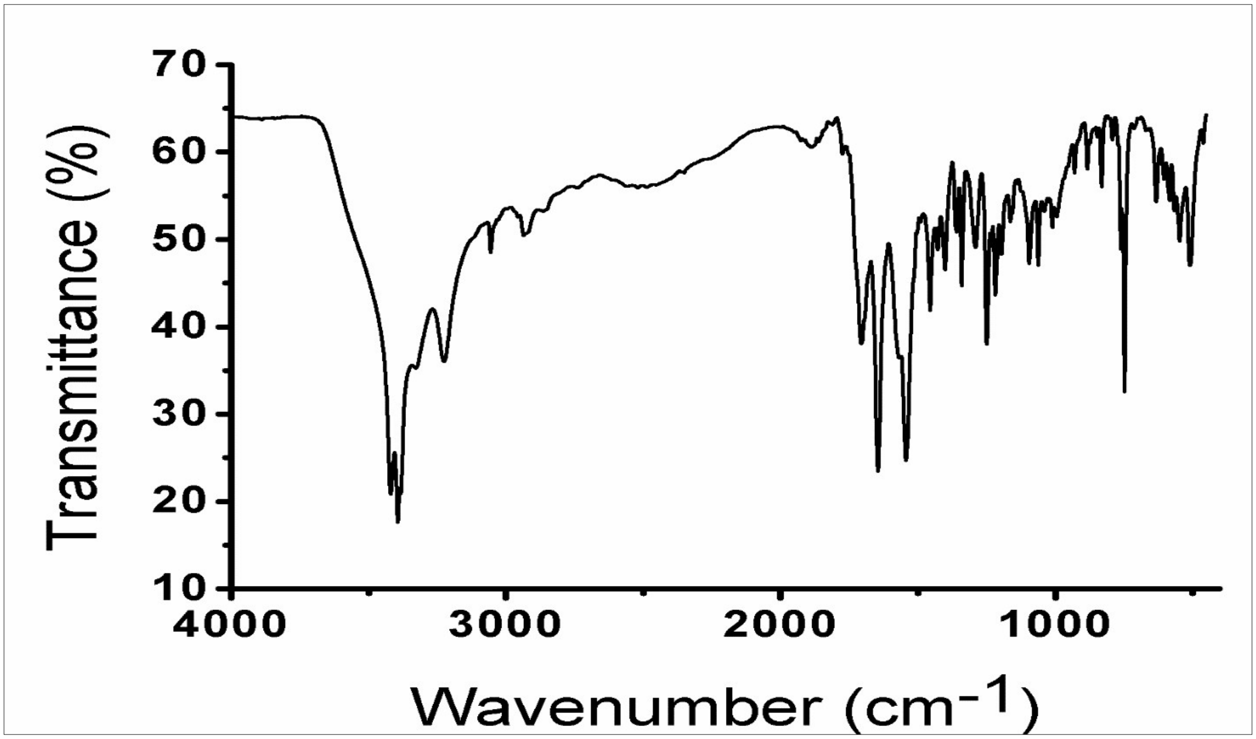

Figure S7: FT-IR Spectra of Compound **1**

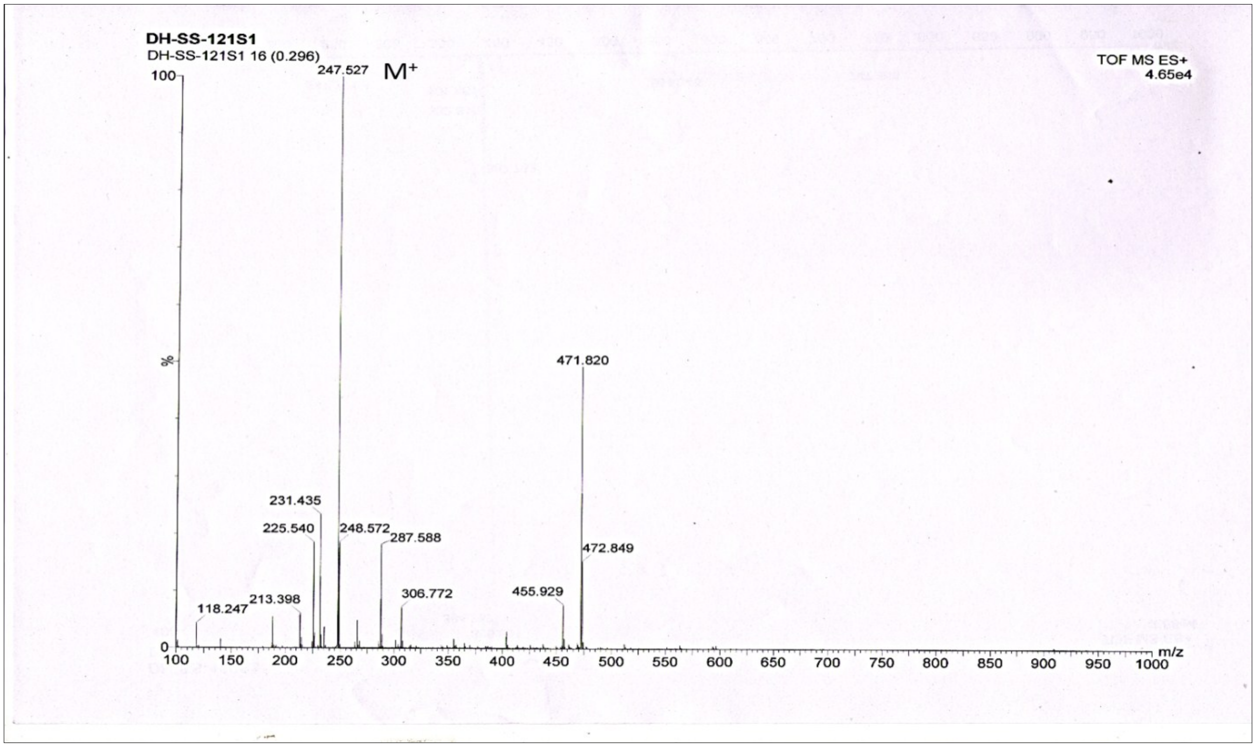

Figure S8: ESI-MS Spectra of Compound **1**

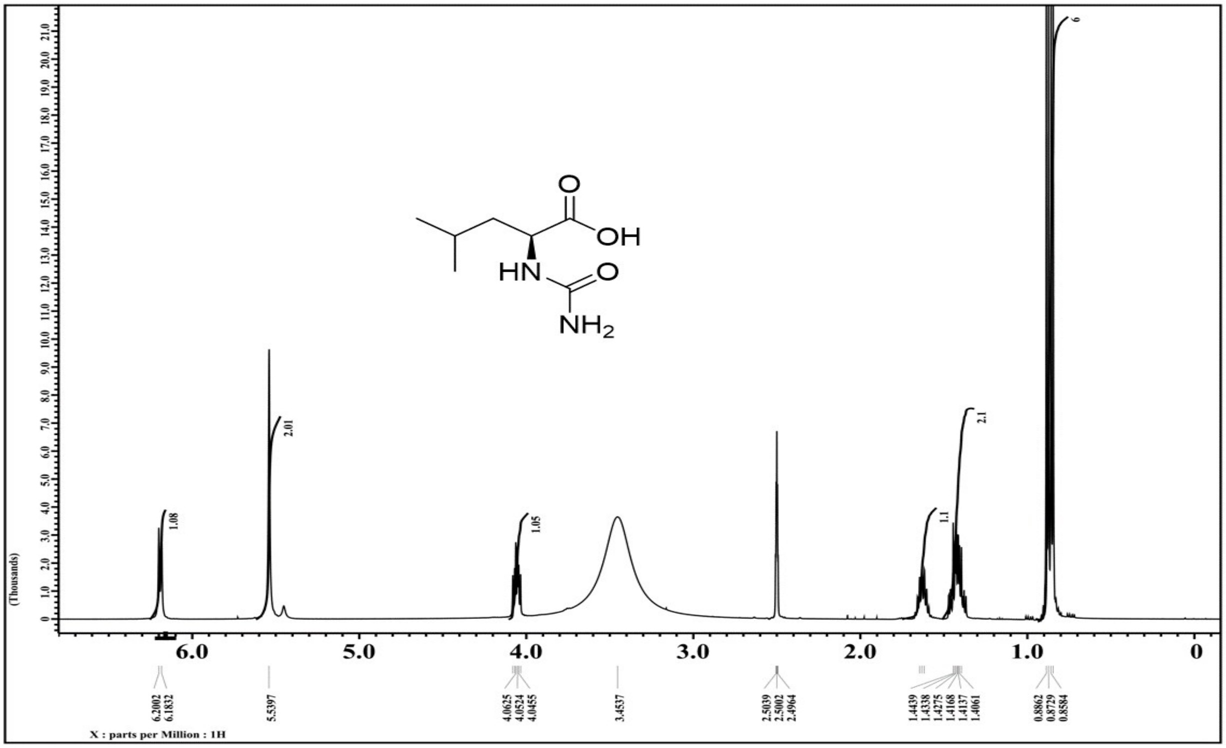

Figure S9: ^{1}H NMR (400 MHz, DMSO-d_6) spectra of Compound **2**.

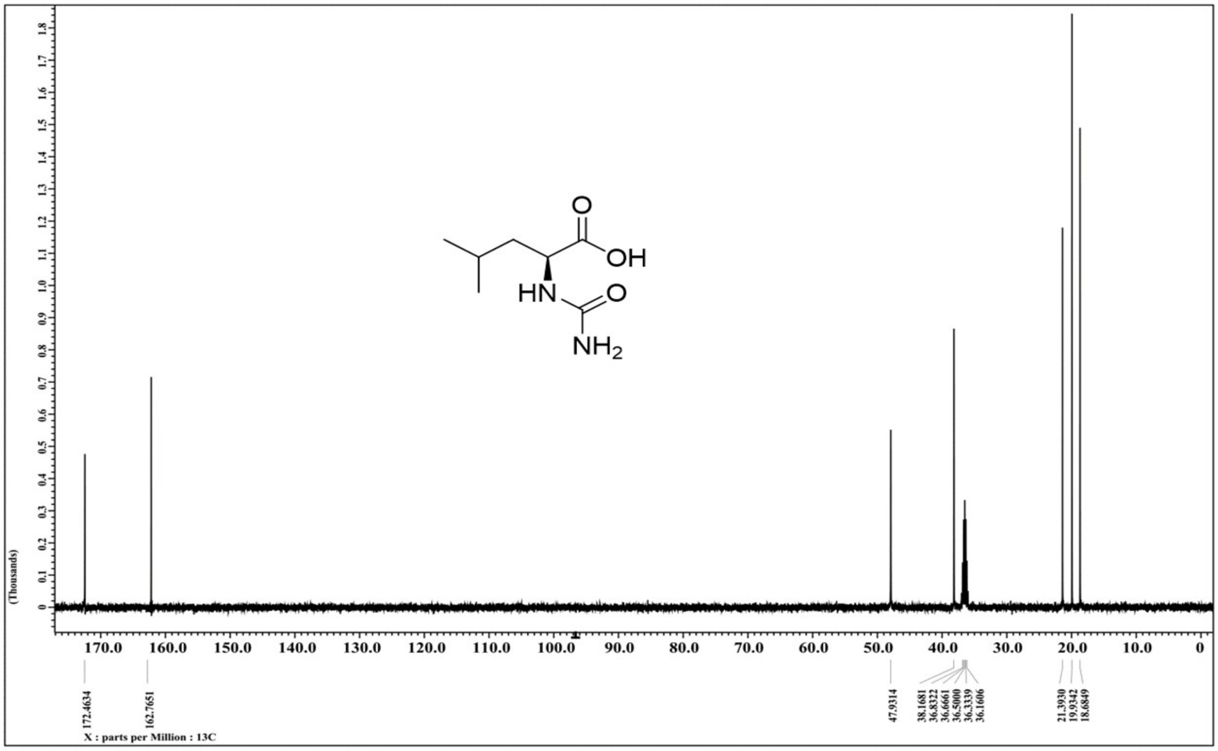

Figure S10: ^{13}C NMR (100 MHz, DMSO-d_6) spectra of Compound **2**.

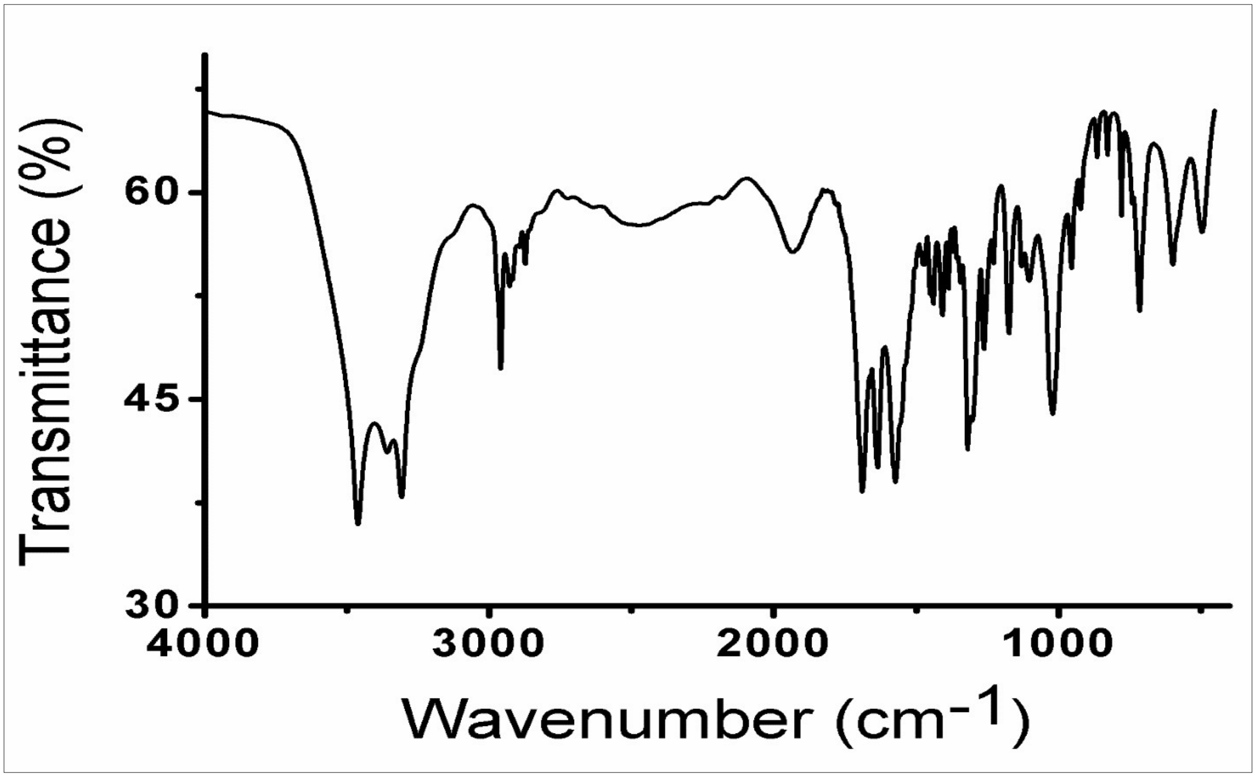

Figure S11: FT-IR Spectra of Compound **2**

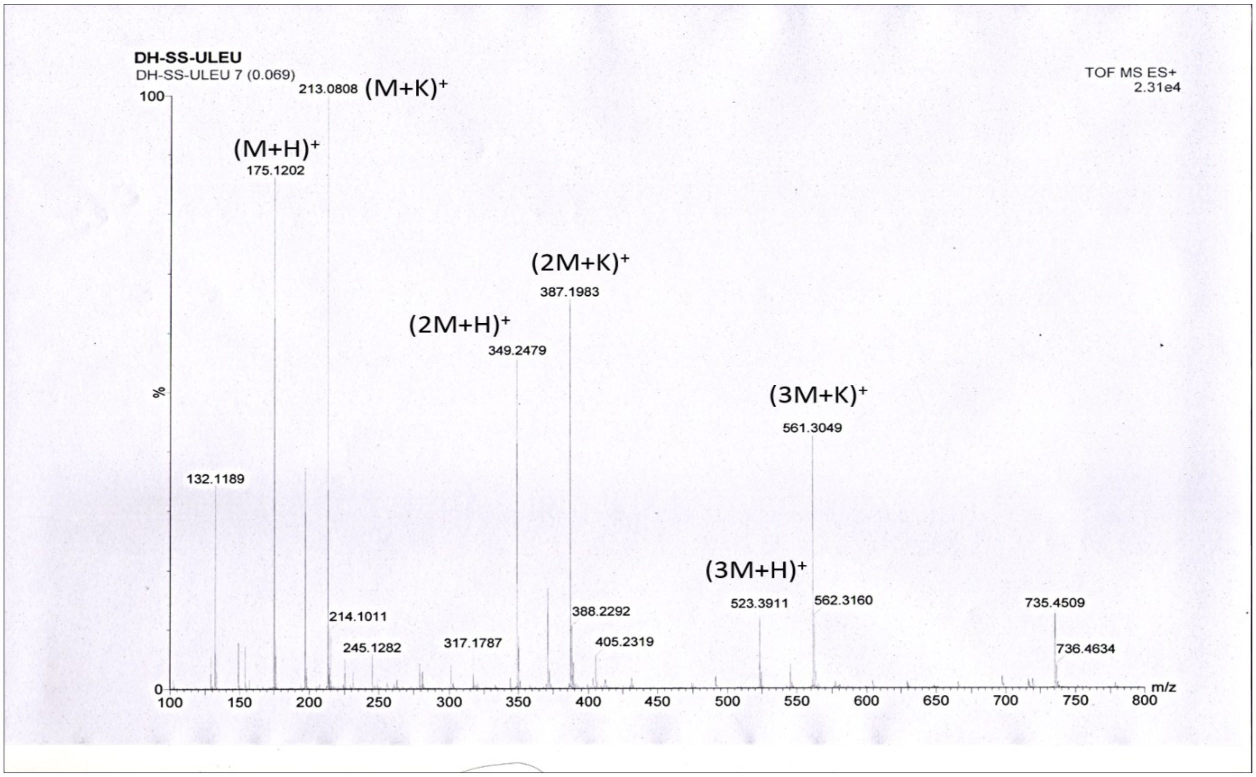

Figure S12: ESI-MS Spectra of Compound **2**

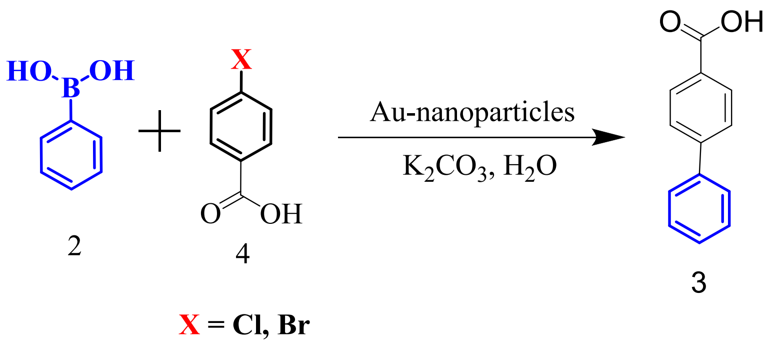

Scheme 2. Schematic presentation of catalysis of Suzuki–Miyaura cross-coupling reaction between arylbromide and arylboronic acids in water using urea derivatives **1** decorated Au-nanoparticles.

Suzuki–Miyaura cross-coupling reaction:

A mixture of aryl halide (1 mmol), Phenylboronic acid (1.5 mmol) and K_2CO_3 (3 mmol) was dissolved in H_2O in a 15 mL round bottomed flask. Aqueous compound **1**-gold nanoparticles (0.01 mol %) catalytic solution was added to it and the mixture was stirred for a time period and temperature as mentioned in Table 1. The progress of the reaction was monitored by TLC. After completion of the reaction and acidified it with 2(N) HCl product was precipitated out for **3a-c, 3f-h, 3k** and separate layer separated out for **3d,e,i,j.** The products were characterized by IR, ^{1}H NMR, ^{13}C NMR and ESI–MS.

Biphenyl-4-Carboxylic Acid (3a).

White solid, 198 mg, 98.37 % yield; m.p.: 185°C.

^{1}H NMR (400 MHz, DMSO-d$_6$. δ$_{ppm}$, 298 K): 12.93 [s, 1H, -CO$_2$**H**], 8.02 [d, 2H, *J* = 8.54, -Ar^1**H**], 7.79 [d, 2H, *J* = 7.93, -Ar^1**H**], 7.72 [d, 2H, *J* = 7.93, -Ar^2**H**], 7.49 [d, 2H, *J* = 7.32, -Ar^2**H**], 7.41 [t, 1H, *J* = 7.32, -Ar^2**H**];

^{13}C NMR (100 MHz, DMSO-d$_6$. δ$_{ppm}$, 298 K): 144.33, 139.05, 130.01, 129.67, 129.32, 126.99, 126.85;

FT-IR (KBr): 2984, 2840, 2667, 2552, 1682, 1608, 1423, 1292, 936, 863, 752, 698 cm^{-1};

ESI-MS: m/z 236.86 [M+K]$^+$; M_{calcd}:198.22.

Biphenyl-4-Carboxylic Acid (3b).

White solid, 325 mg, 78 % yield; m.p: 187°C.

^{1}H NMR (400 MHz, DMSO-d$_6$. δ_{ppm}, 298 K): 12.91 [s, 1H, -CO$_2$**H**], 8.03 [d, 2H, J = 8.51, -Ar^1**H**], 7.79 [d, 2H, J = 7.95, -Ar^1**H**], 7.71 [d, 2H, J = 7.98, -Ar^2**H**], 7.49 [d, 2H, J = 7.30, -Ar^2**H**], 7.42 [t, 1H, J = 7.35, -Ar^2**H**];

^{13}C NMR (100 MHz, DMSO-d$_6$. δ_{ppm}, 298 K): 144.34, 139.05, 130.08, 129.67, 129.32, 126.99, 126.85; 126.85;

FT-IR (KBr): 2987, 2837, 2670, 2542, 1688, 1612, 1428, 1295, 938, 869, 755, 698 cm^{-1}

ESI-MS: m/z 235.96 [M+K]$^+$; M_{calcd}:198.22.

Biphenyl-2,4-diol (3c).

Light brown solid, 287 mg, 89.64 % yield; m.p: 285°C.

^{1}H NMR (400 MHz, CDCl$_3$. δ_{ppm}, 298 K): 8.25 [d, 1H, J = 6.71, -Ar^2**H],** 7.75 [d, 1H, J = 6.71, -Ar^2**H]**, 7.61 [t, 1H, J = 7.32, -Ar^2**H]**, 7.52 [t, 1H, J = 7.32, -Ar^2**H]**, 7.42 [m, 1H, -Ar^2**H]**, 7.14 [d, 1H, J = 9.16, -Ar^1**H]**, 6.53 [d, 1H, J = 7.22, -Ar^1**H]**, 6.38 [dd, 1H, J = 8.54, -Ar^1**H]**, 5.6 [s, 1H, -O**H]**, 5.3 [s, 1H, -O**H]**;

^{13}C NMR (100 MHz, CDCl$_3$. δ_{ppm}, 298 K): 156.13, 152.40, 135.99, 133.80, 133.05, 129.67, 128.33, 111.96, 109.17, 103.87; 126.85;

FT-IR (KBr): 3442, 3315, 3074, 2927, 2851, 2362, 1603, 1442, 1342, 1310, 1159, 1084, 1023, 966, 834, 697, 579 cm$^-$1;

ESI-MS: m/z 225.14 [M+K]$^+$; M_{calcd}: 186.07.

1-(biphenyl-4-yl)ethanone (3d).

Light brown liquid, 288 mg, 88 % yield;

^{1}H NMR (400 MHz, DMSO-d$_6$, δ_{ppm}, 298 K): 7.92 [d, 2H, J = 8.39, -Ar^1**H],** 7.79 [d, 2H, J = 6.87, -Ar^1**H]**, 7.53 [d, 2H, J = 8.39, -Ar^2**H]**, 7.36 [d, 2H, J = 6.87, -Ar^2**H]**, 7.30 [t, 1H, J = 7.25, -Ar^2**H]**, 2.54 [s, 3H, -C**H**$_3$**]**;

^{13}C NMR (100 MHz, DMSO-d$_6$, δ_{ppm}, 298 K): 197.16, 138.31, 135.25, 130.19, 128.90, 127.51, 26.80; 126.85;

FT-IR (KBr): 1680, 1585, 1446, 1395, 1359, 1260, 1093, 1010, 952, 825, 761, 697 cm$^-$1;

ESI-MS: m/z 219.91 [M+Na]$^+$; M_{calcd}: 196.09.

Biphenyl-2-carbaldehyde (3e).

Brown liquid, 270 mg, 96 % yield;

^{1}H NMR (500 MHz, DMSO-d$_6$, δ$_{ppm}$, 298 K): 10.34 [s, 1H, -C**H**O], 8.01 [d, 1H, *J* = 6.62, -Ar^1**H]**, 7.86 [t, 1H, *J* = 5.99, -Ar^1**H]**, 7.83 [d, 1H, *J* = 7.25, -Ar^1**H]**, 7.67 [t, 2H, *J* = 7.25, -Ar^1**H]**, 7.58 [d, 2H, *J* = 7.25, -Ar^2**H]**, 7.50 [d, 2H, *J* = 7.57, -Ar^2**H]**, 7.43 [m, 1H, -Ar^2**H]**;

^{13}C NMR (125 MHz, DMSO-d$_6$, δ$_{ppm}$, 298 K): 189.86, 135.82, 134.42, 134.17, 130.78, 129.73, 127.92, 127.40 ; 126.85;

FT-IR (KBr): 3646, 3050, 1780, 1690, 1591, 1443, 1302, 1271, 1182, 1111, 1069, 1051, 1028 cm^{-1};

ESI-MS: m/z 221.02 [M+K]$^+$; *M*$_{calcd}$: 182.22. λ = 193 nm (ε = 2.67 X 10^5 M^{-1} cm^{-1}), 210 nm (ε = 1.5 X 10^4 M^{-1} cm^{-1}), 249 nm (ε = 4.17 X 10^3 M^{-1} cm^{-1}).

2,4-dimethoxy-6-phenyl-1,3,5-triazine (3f).

Light brown semisolid, 255 mg, 97 % yield;

^{1}H NMR (400 MHz, DMSO-d$_6$, δ$_{ppm}$, 298 K): 8.01 [d, 2H, *J* = 7.63, -Ar^2**H]**, 7.85 [d, 2H, *J* = 7.63, -Ar^2**H]**, 7.81 [t, 1H, *J* = 6.10, -Ar^2**H]**, 3.91 [s, 6H, -OC**H$_3$]**;

^{13}C NMR (100 MHz, DMSO-d$_6$, δ$_{ppm}$, 298 K): 173.17, 134.16, 130.88, 130.05, 127.42, 126.53 ; 126.85;

FT-IR (KBr): 3480, 3256, 3090, 2254, 2128, 1655, 1440, 1367, 1024, 996, 824, 767, 704, 643 cm^{-1}

ESI-MS: m/z 242.22 [M+Na]$^+$; *M*$_{calcd}$: 218.22.

2-methoxy-4,6-diphenyl-1,3,5-triazine (3g).

Brown semisolid, 315 mg, 95 % yield;

^{1}H NMR (400 MHz, DMSO-d$_6$, δ$_{ppm}$, 298 K): 8.01 [d, 4H, *J* = 7.63, -Ar^2**H]**, 7.85 [d, 4H, *J* = 7.63, -Ar^2**H]**, 7.82 [t, 2H, *J* = 6.10, -Ar^2**H]**, 3.92 [s, 3H, -OC**H$_3$]**;

^{13}C NMR (100 MHz, DMSO-d$_6$, δ$_{ppm}$, 298 K): 173.16, 134.16, 130.88, 130.06, 127.42, 126.53 ;

ESI-MS: m/z 301.07 [M+K]$^+$; *M*$_{calcd}$: 263.29.

2,4,6-triphenyl-1,3,5-triazine (3h).

Brown semisolid, 375 mg, 94 % yield;

^{1}H NMR (400 MHz, DMSO-d$_6$, δ_{ppm}, 298 K): 8.20-7.26 [m, 15H, -Ar^2**H**];

^{13}C NMR (100 MHz, DMSO-d$_6$, δ_{ppm}, 298 K): 177.88, 135.33, 133.33, 132.24, 128.04;

ESI-MS: m/z 310.36 [M+H]$^+$; M_{calcd}: 309.22.

4-vinylbiphenyl (3i).

White liquid, 346 mg, 75 % yield;

^{1}H NMR (400 MHz, DMSO-d$_6$, δ_{ppm}, 298 K): 7.74 [d, 2H, J = 8.24, -Ar^1**H],** 7.68 [d, 2H, J = 8.17, -Ar^1**H]**, 7.48 [d, 2H, J = 8.11, -Ar^2**H]**, 7.38 [d, 2H, J = 8.25, - Ar^2**H]**, 7.22 [t, 1H, J = 8.31, - Ar^2**H]**, 6.66 [t, 1H, J = 17.92, -C**H**], 5.84 [d, 1H, J = 18.32, -C**H**$_2$], 5.29 [d, 1H, J = 8.44, -C**H**$_2$];

^{13}C NMR (100 MHz, DMSO-d$_6$, δ_{ppm}, 298 K): 134.42, 134.26, 134.16, 130.88, 130.85, 127.59, 127.42, 126.53 ; 126.85;

FT-IR (KBr): 3490, 3370, 3252, 3107, 2382, 2258, 2127, 1649, 1025, 997, 824, 767, 474 cm^{-1} ;

ESI-MS: m/z 181.25 [M+H]$^+$; M_{calcd}: 180.25

5-nitrobiphenyl-3-carbaldehyde (3j).

Light brown solid, 243 mg, 81 % yield, m.p. 130°C;

^{1}H NMR (400 MHz, DMSO-d$_6$, δ_{ppm}, 298 K): 10.12 [s, 1H, -C**H**O], 8.66 [s, 1H, -Ar^1**H]**, 8.49 [dd, 1H, J = 8.77, -Ar^1**H]**, 8.30 [d, 1H, J = 7.63, - Ar^1**H]**, 7.86 [t, 2H, J = 8.01, - Ar^2**H**], 7.77 [d, 1H, J = 6.87, - Ar^2**H**], 7.36 [d, 1H, J = 5.34, - Ar^2**H**], 7.30 [t, 1H, J = 7.25, - Ar^2**H**];

^{13}C NMR (100 MHz, DMSO-d$_6$, δ_{ppm}, 298 K): 191.79, 148.25, 137.17, 134.92, 134.10, 133.46, 130.95, 130.01, 128.54, 127.35, 124.05;

ESI-MS: m/z 225.16 [M]$^+$; M_{calcd}: 225.22.

4-phenylpyridine-2,6-dicarboxylic acid (3k).

White solid, 254.33 mg, 78 % yield, m.p. 281°C;

^{1}H NMR (400 MHz, DMSO-d$_6$, δ_{ppm}, 298 K): 11.95 [br, 2H, -CO$_2$**H**], 8.05 [s, 2H, -Ar^1**H]**, 7.8 [t, 2H, J = 8.77, -Ar^2**H]**, 7.32 [t, 2H, J = 7.43, - Ar^2**H]**, 7.39 [t, 1H, J = 7.25, - Ar^2**H**];

^{13}C NMR (100 MHz, DMSO-d$_6$, δ_{ppm}, 298 K): 172.09, 134.39, 134.14, 133.46, 130.06, 127.57, 127.39;

ESI-MS: m/z 522.37 $[2M+K]^+$; M_{calcd}: 243.05.

NMR experiments

All NMR studies were carried out on a Brüker AVANCE 500 MHz spectrometer and Jeol 400 MHz at 278 K. Compound concentrations were in the range 1–10 mM in $CDCl_3$ and $(CD_3)_2SO$.

FT-IR spectroscopy.

All reported solid-state FTIR spectra were obtained with a Perkin Elmer Spectrum RX1 spectrophotometer with the KBr disk technique.

Mass spectrometry.

Mass spectra were recorded on a Q-Tof Micro YA263 high-resolution (Waters Corporation) mass spectrometer by positive-mode electrospray ionization.

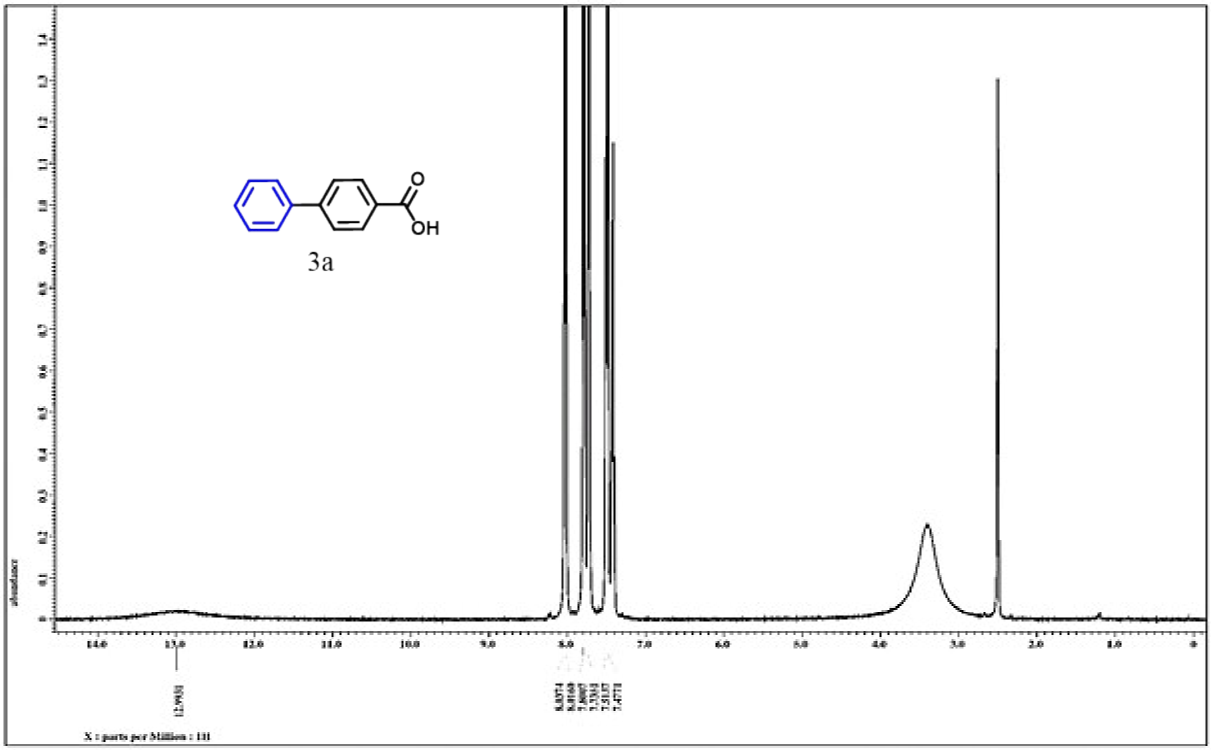

Figure S13: ^{1}HNMR (400 MHz, DMSO-d$_6$. δ_{ppm}, 298 K) spectra of Compound **3a.**

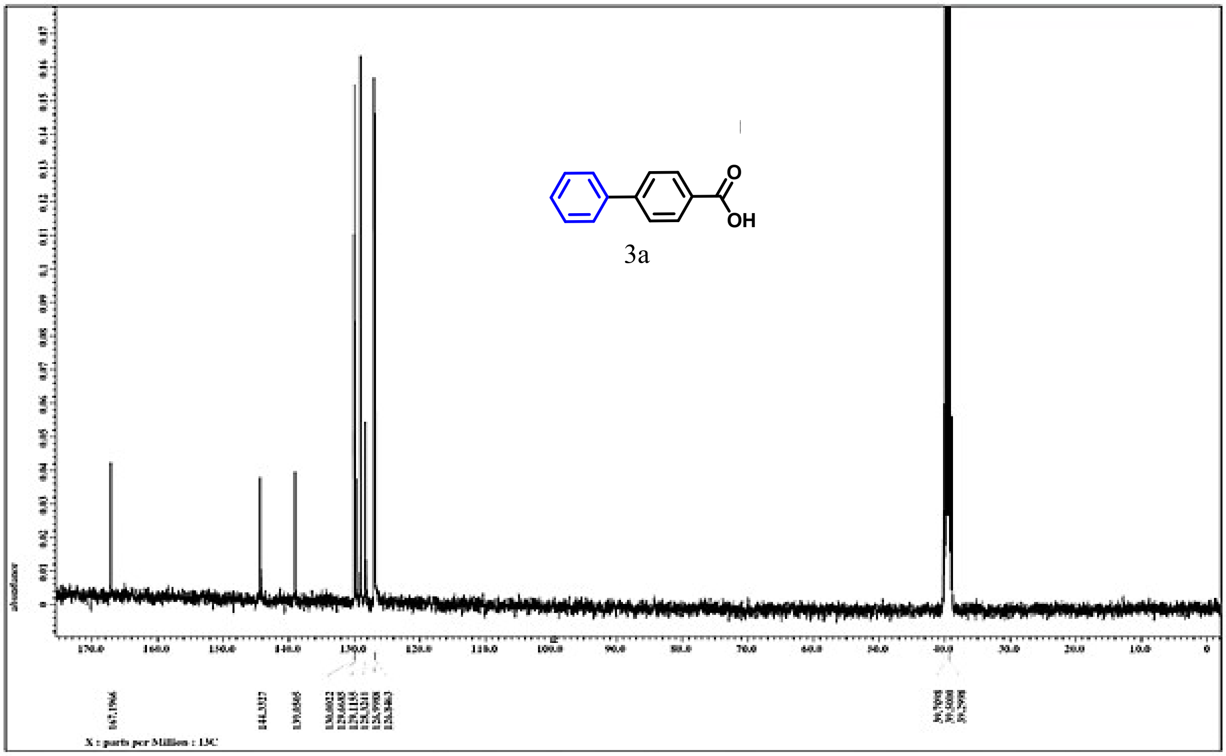

Figure S14: ^{13}CNMR (100 MHz, DMSO-d$_6$. δ_{ppm}, 298 K) spectra of Compound **3a.**

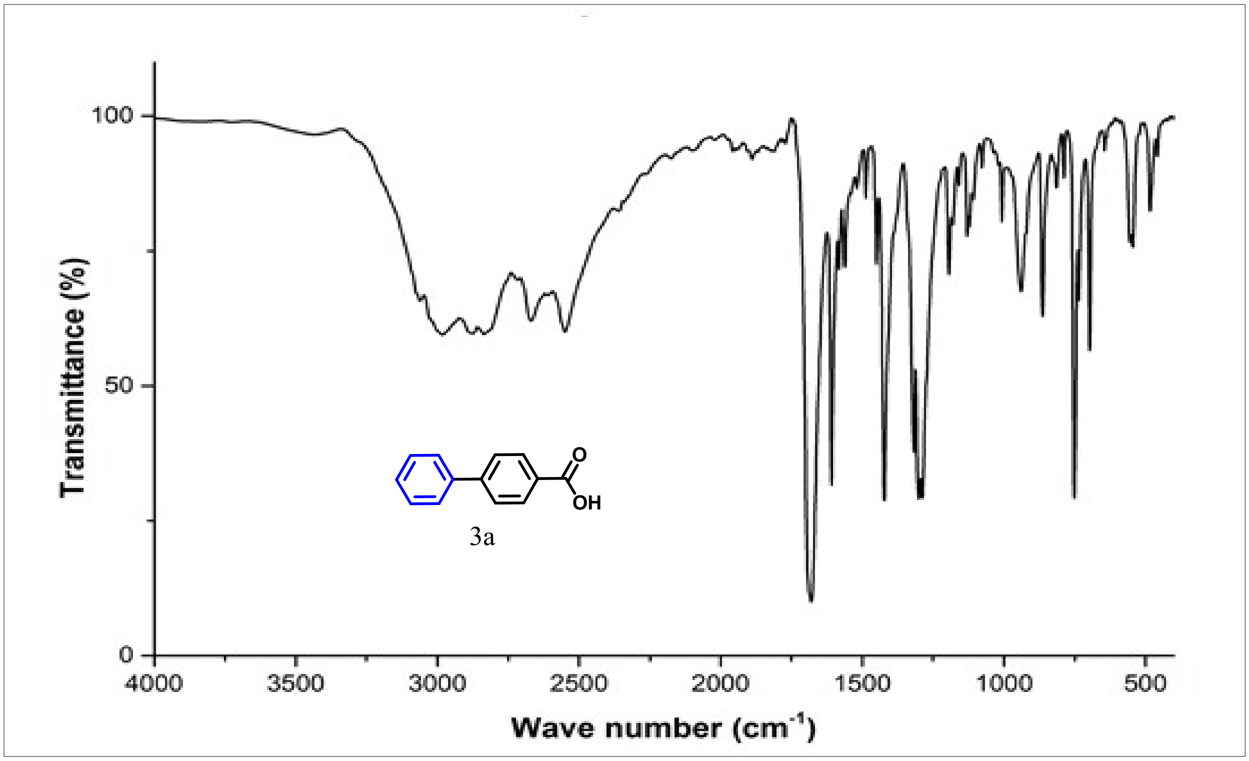

Figure S15: FT-IR Spectra of Compound **3a**

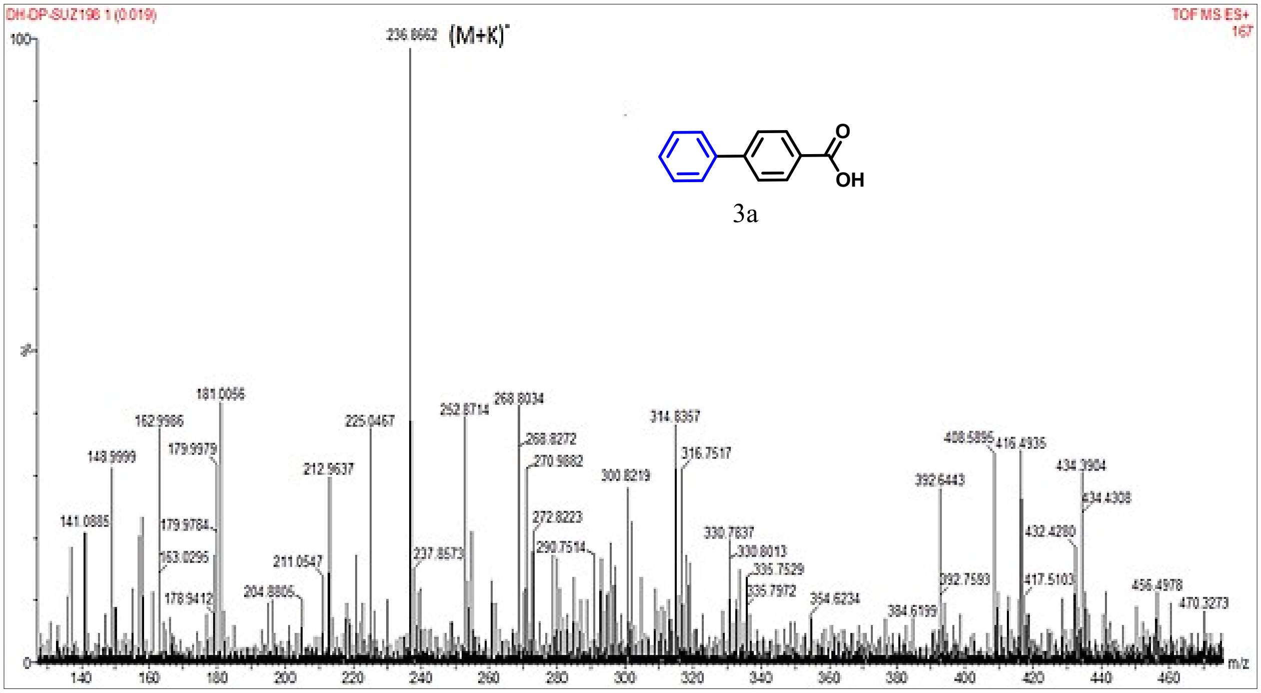

Figure S16: ESI-MS Spectra of Compound **3a**

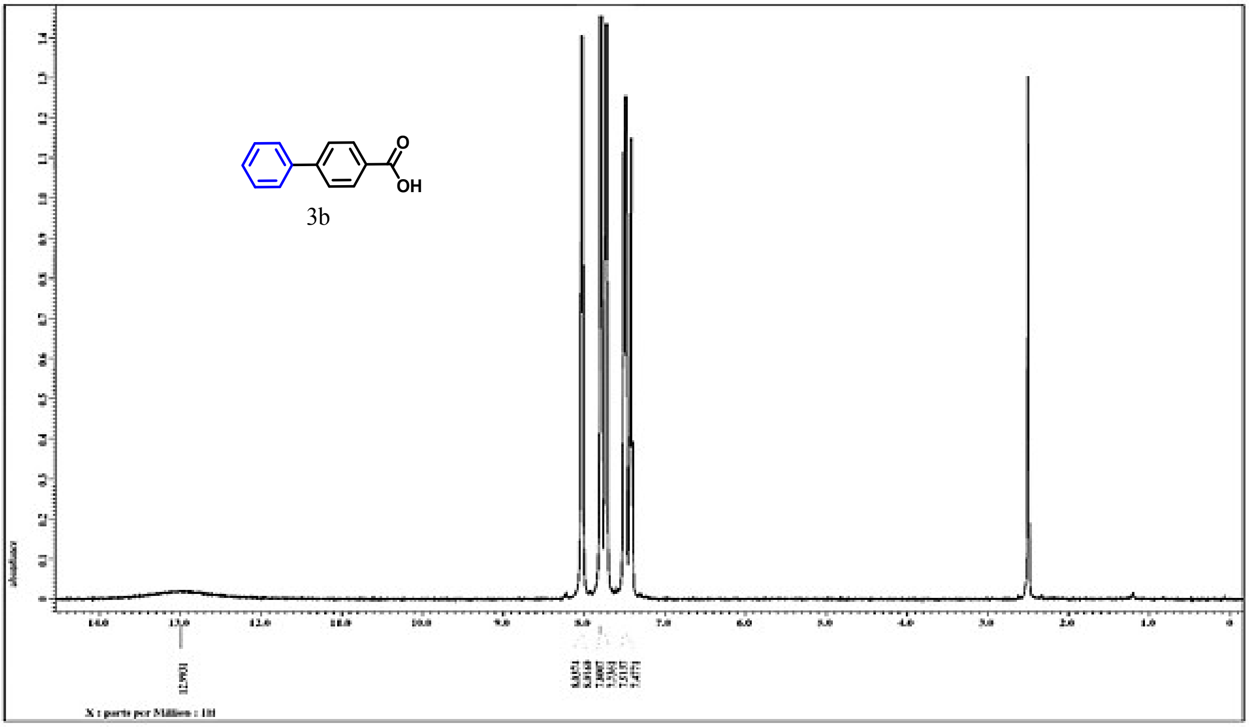

Figure S17: ^{1}HNMR (400 MHz, DMSO-d$_6$. δ$_{ppm}$, 298 K) spectra of Compound **3b.**

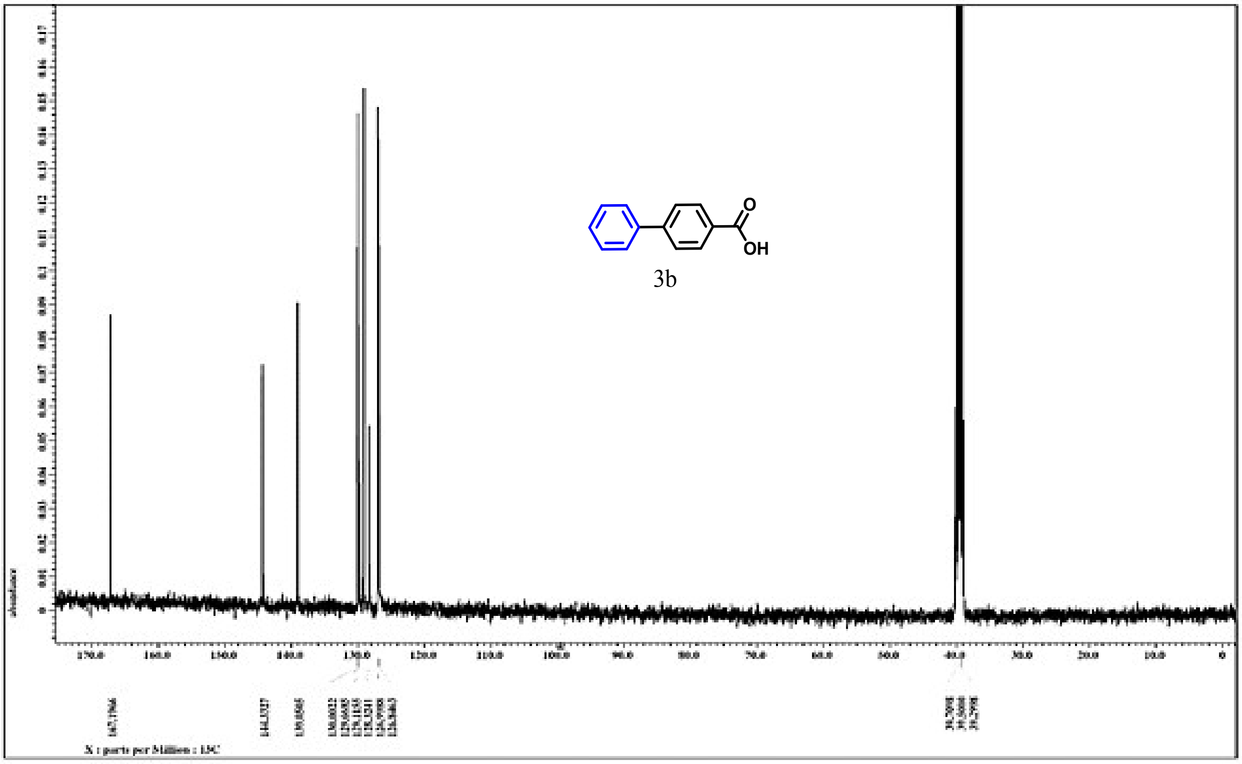

Figure S18: ^{13}CNMR (100 MHz, DMSO-d$_6$. δ$_{ppm}$, 298 K) spectra of Compound **3b.**

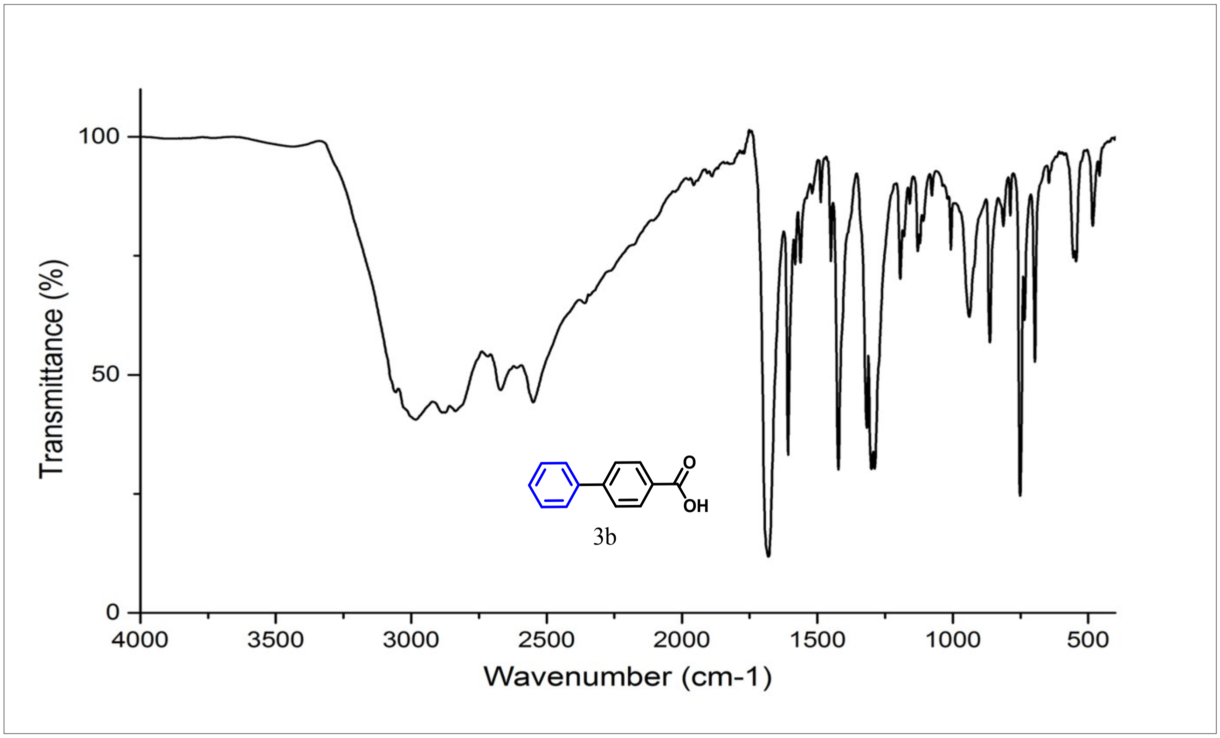

Figure S19: FT-IR Spectra of Compound **3b.**

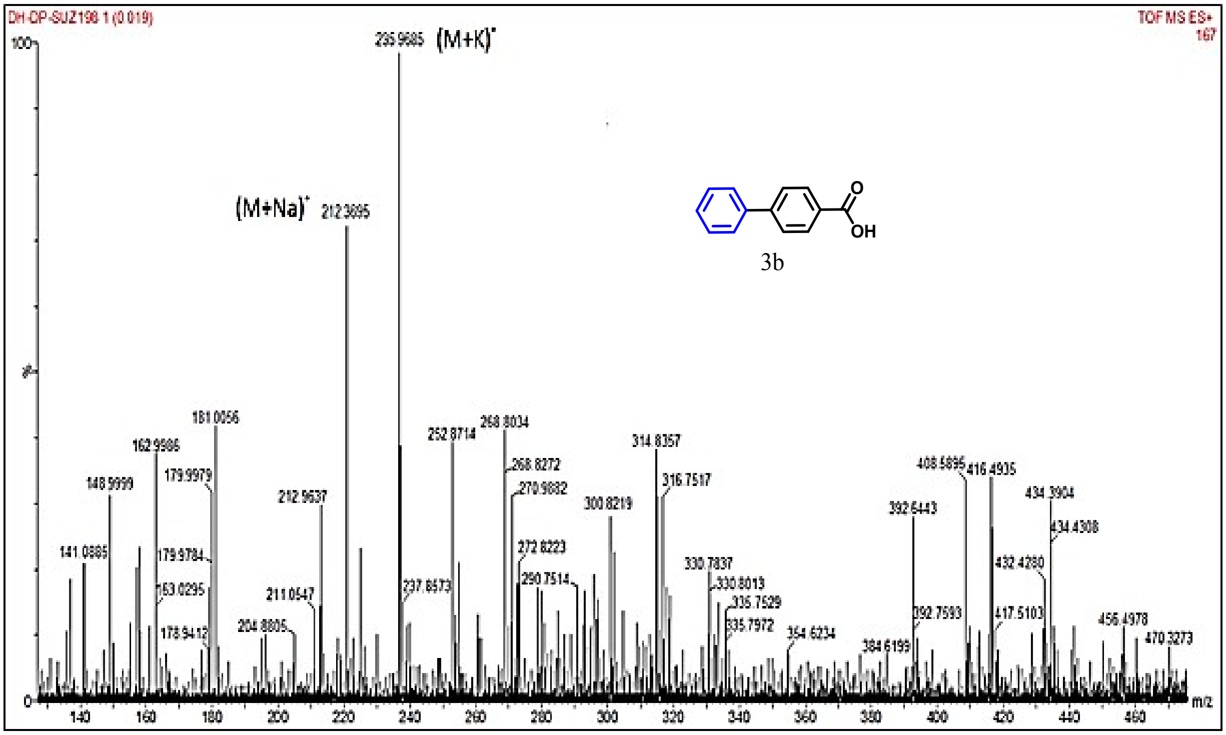

Figure S20: ESI-MS Spectra of Compound **3b.**

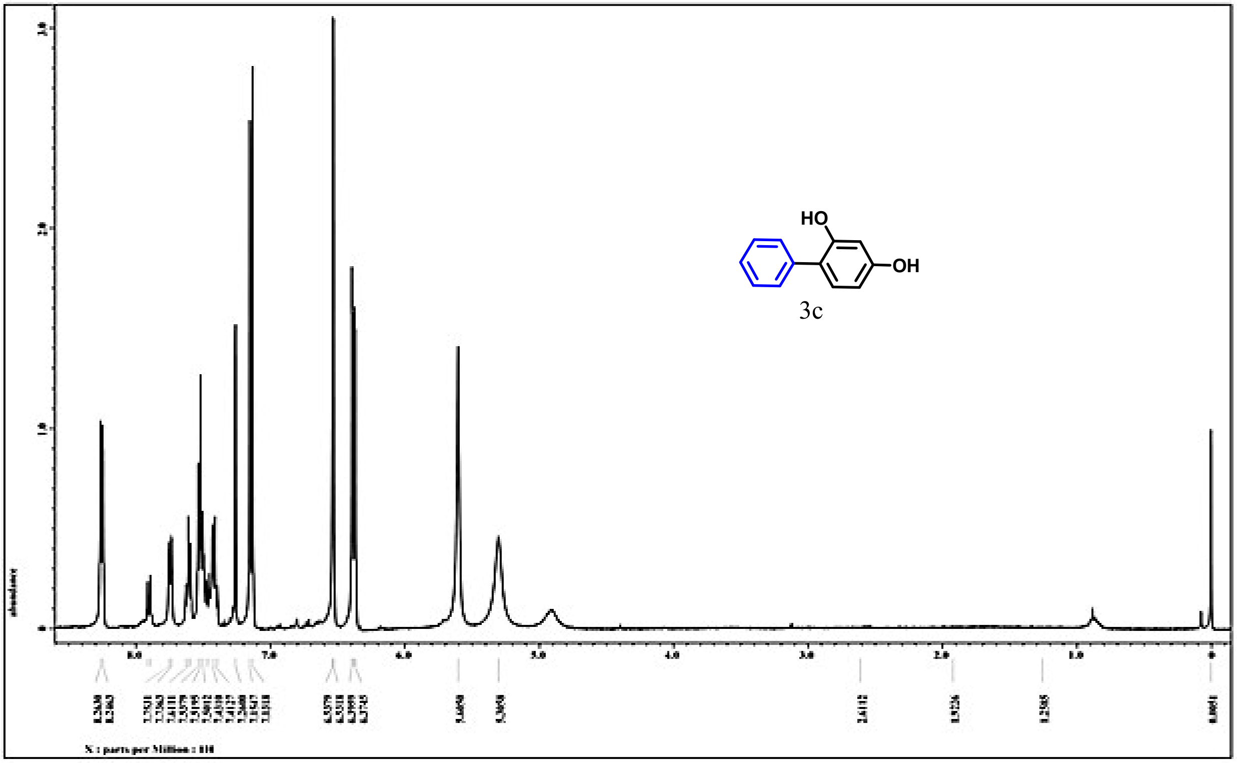

Figure S20: ^{1}HNMR (400 MHz, $CDCl_3$. δ_{ppm}, 298 K) spectra of Compound **3c.**

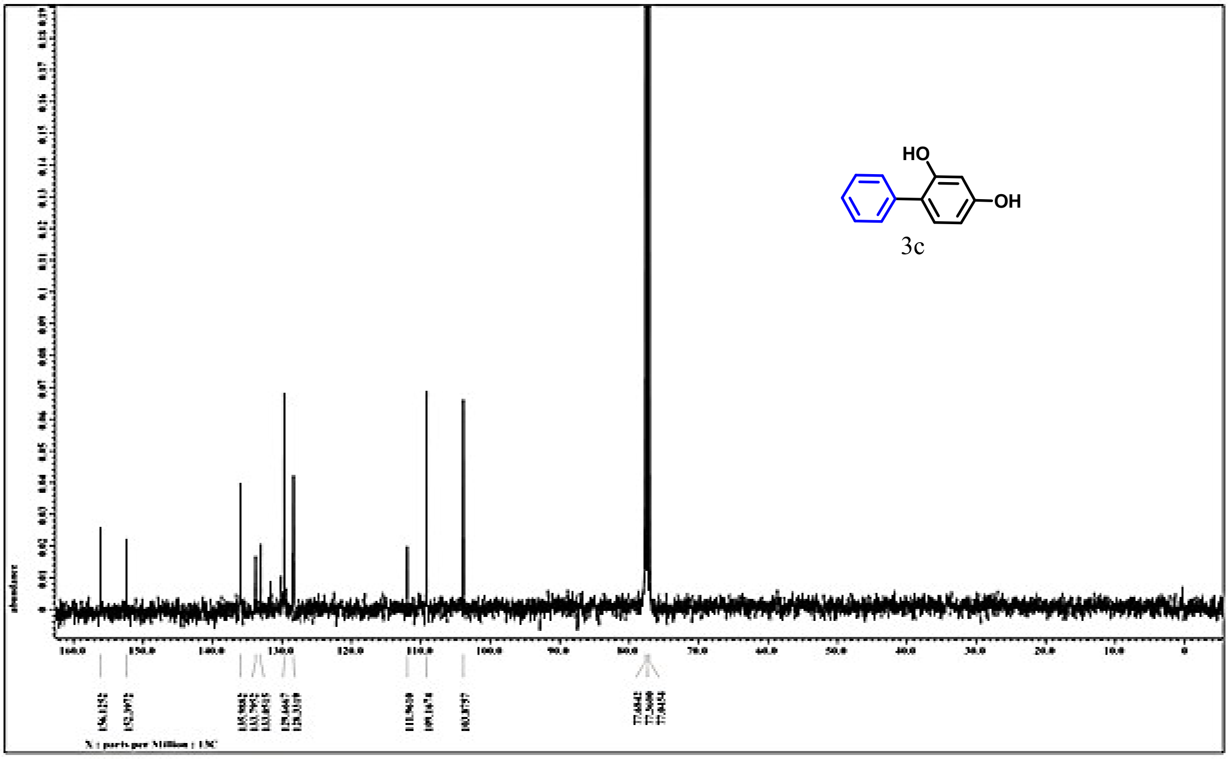

Figure S22: ^{13}CNMR (100 MHz, $CDCl_3$. δ_{ppm}, 298 K) spectra of Compound **3c.**

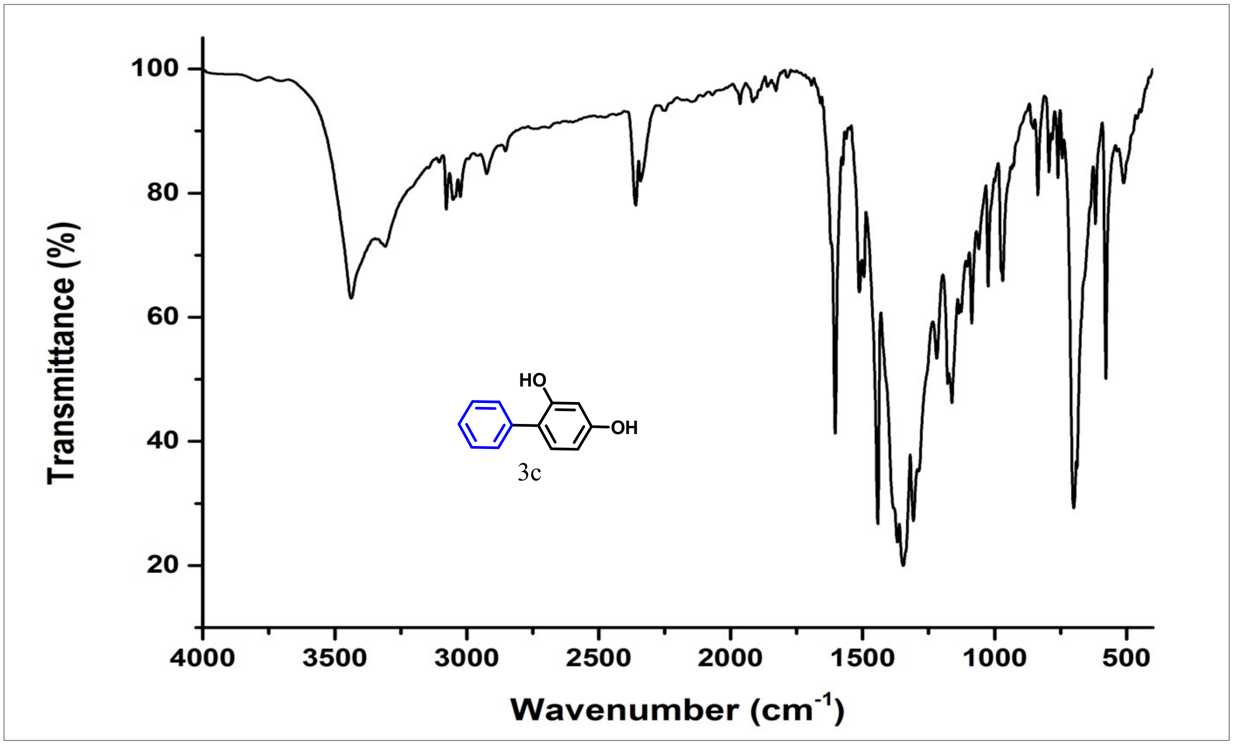

Figure S23: FT-IR Spectra of Compound **3c.**

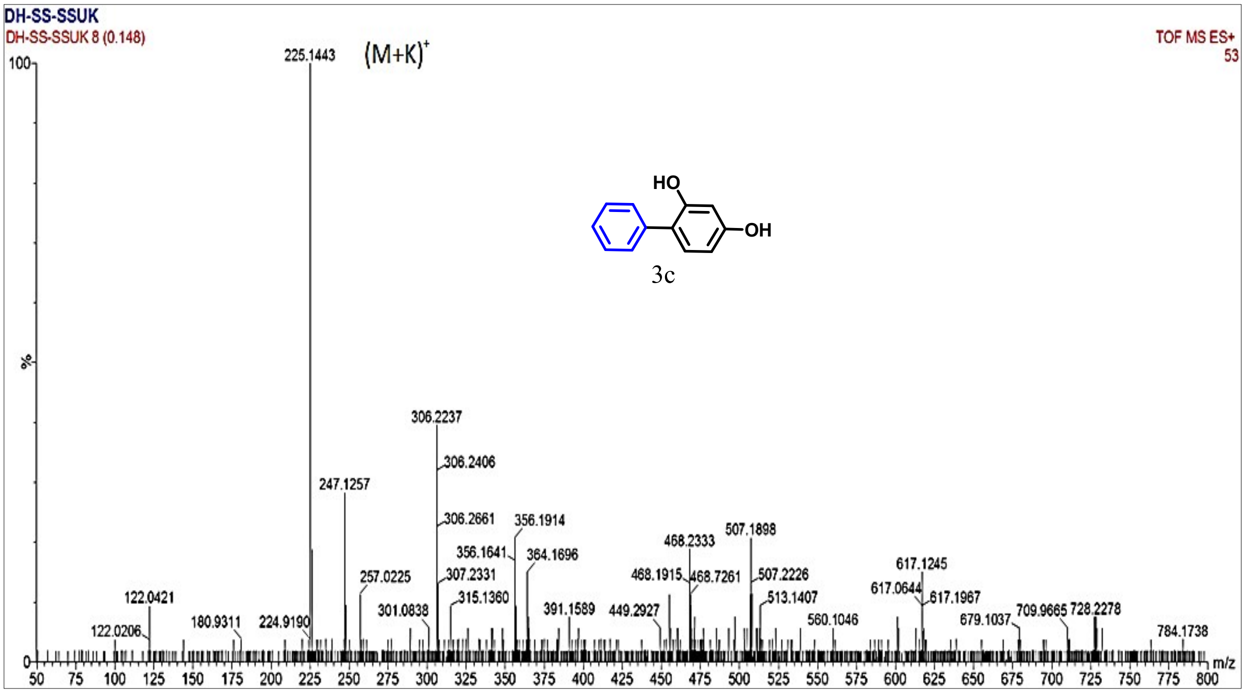

Figure S24: ESI-MS Spectra of Compound **3c.**

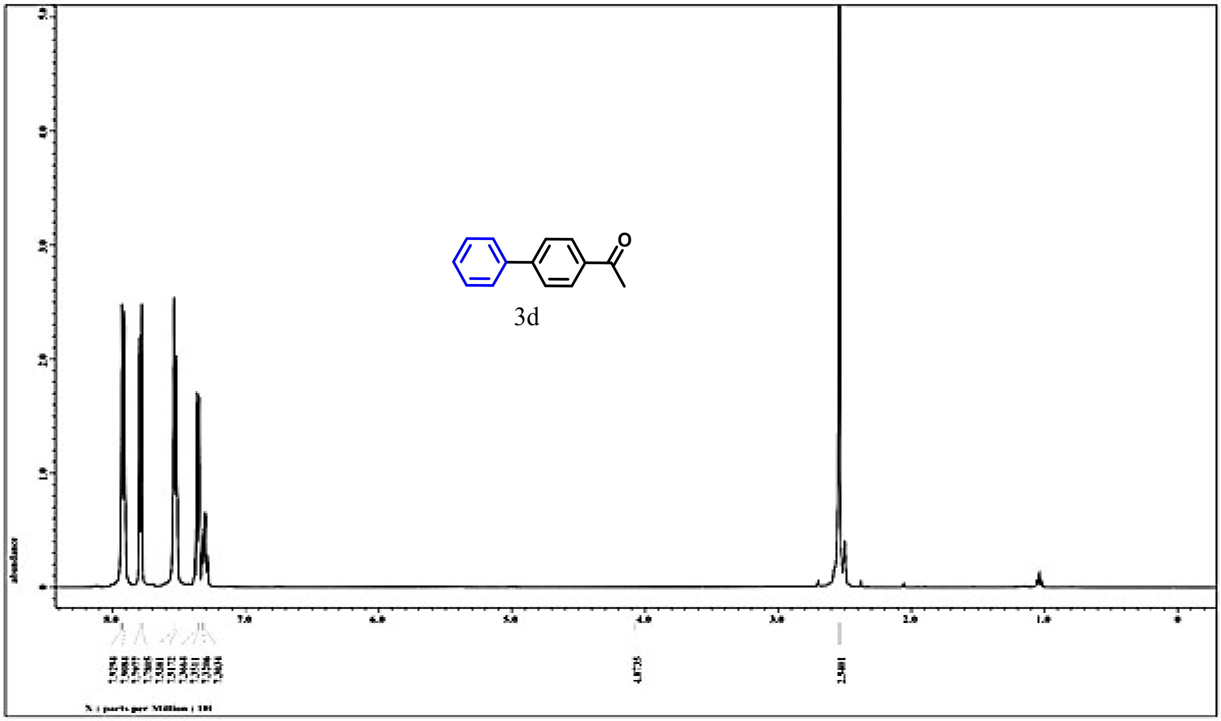

ESI Figure S25: ^{1}HNMR (400 MHz, DMSO-d$_6$. δ_{ppm}, 298 K) spectra of Compound **3d.**

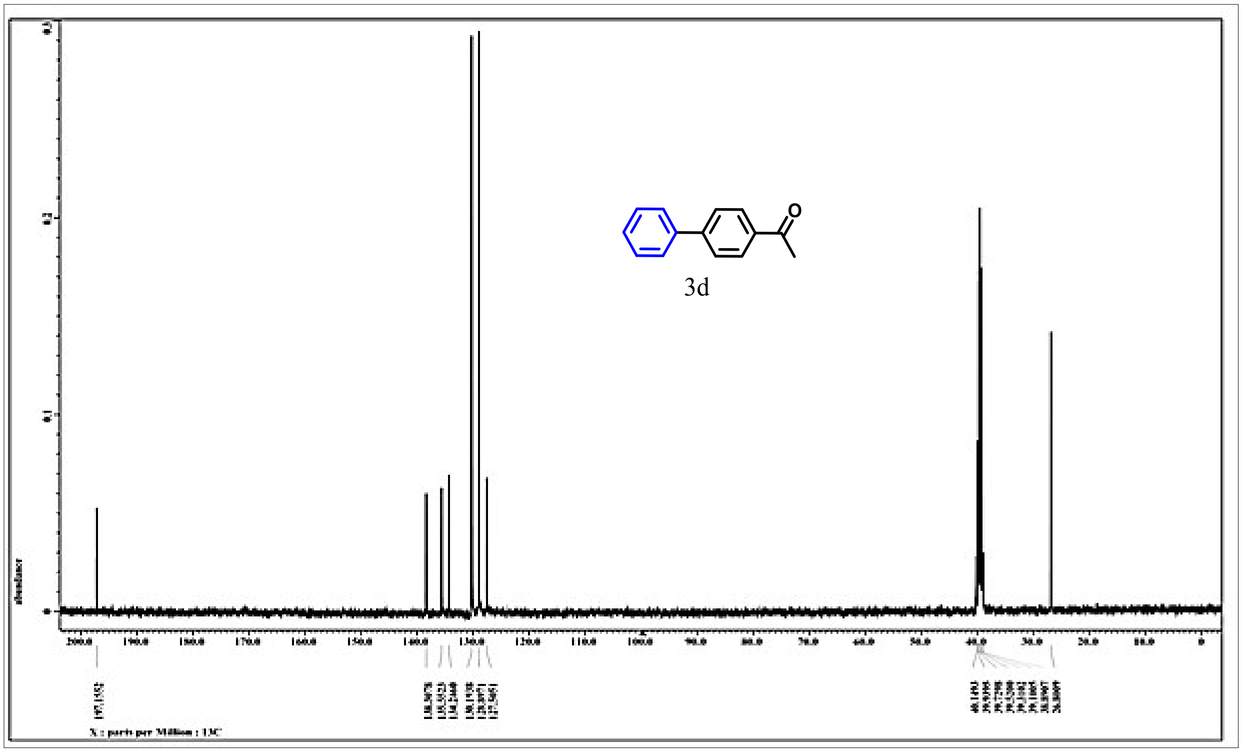

Figure S26: ^{13}CNMR (100 MHz, DMSO-d$_6$. δ_{ppm}, 298 K) spectra of Compound **3d.**

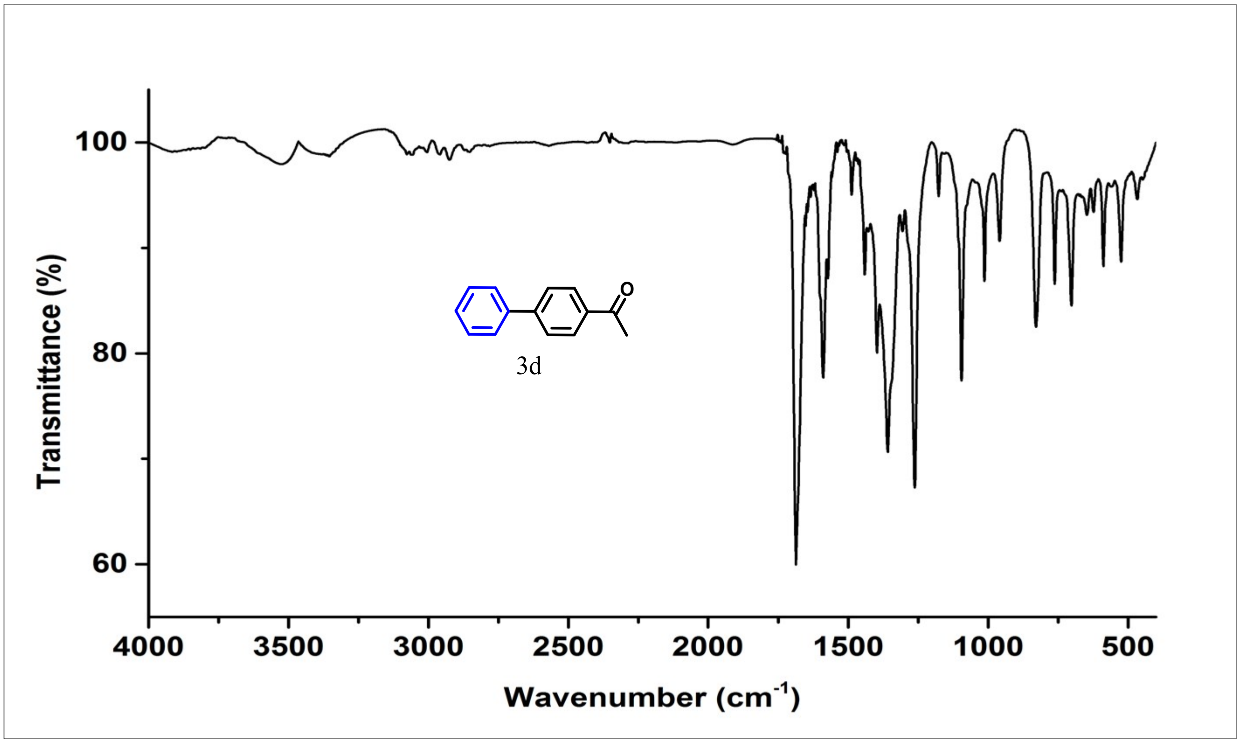

Figure S27: FT-IR Spectra of Compound **3d.**

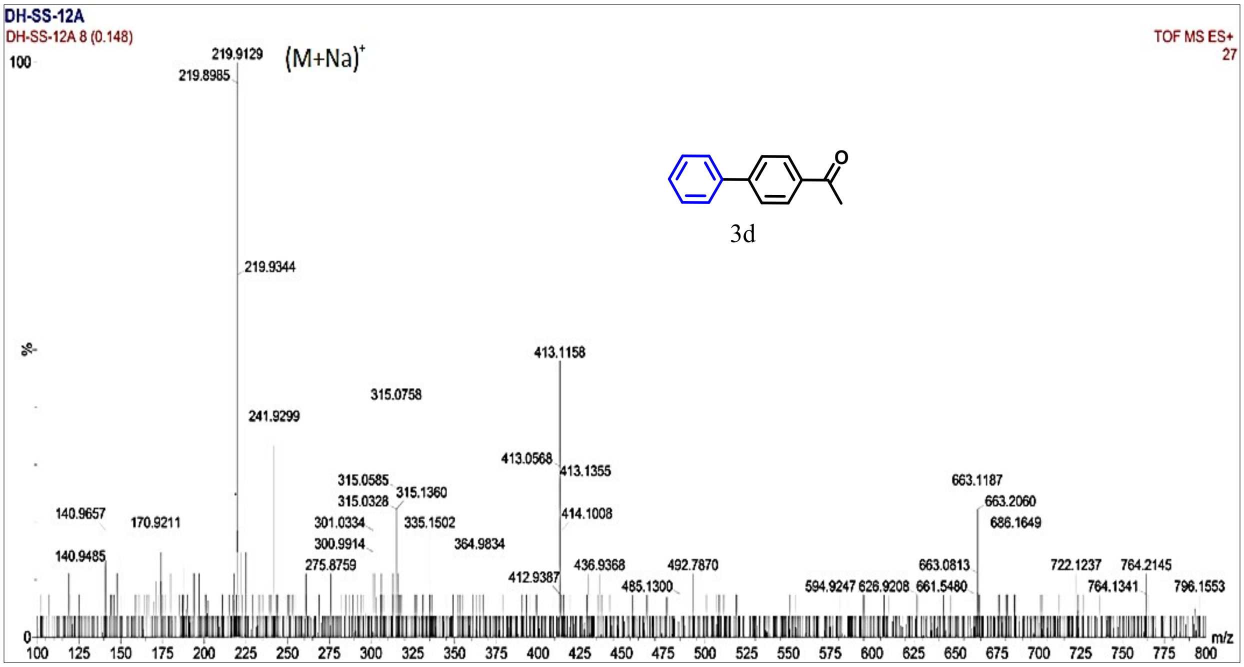

ESI Figure S28: ESI-MS Spectra of Compound **3d.**

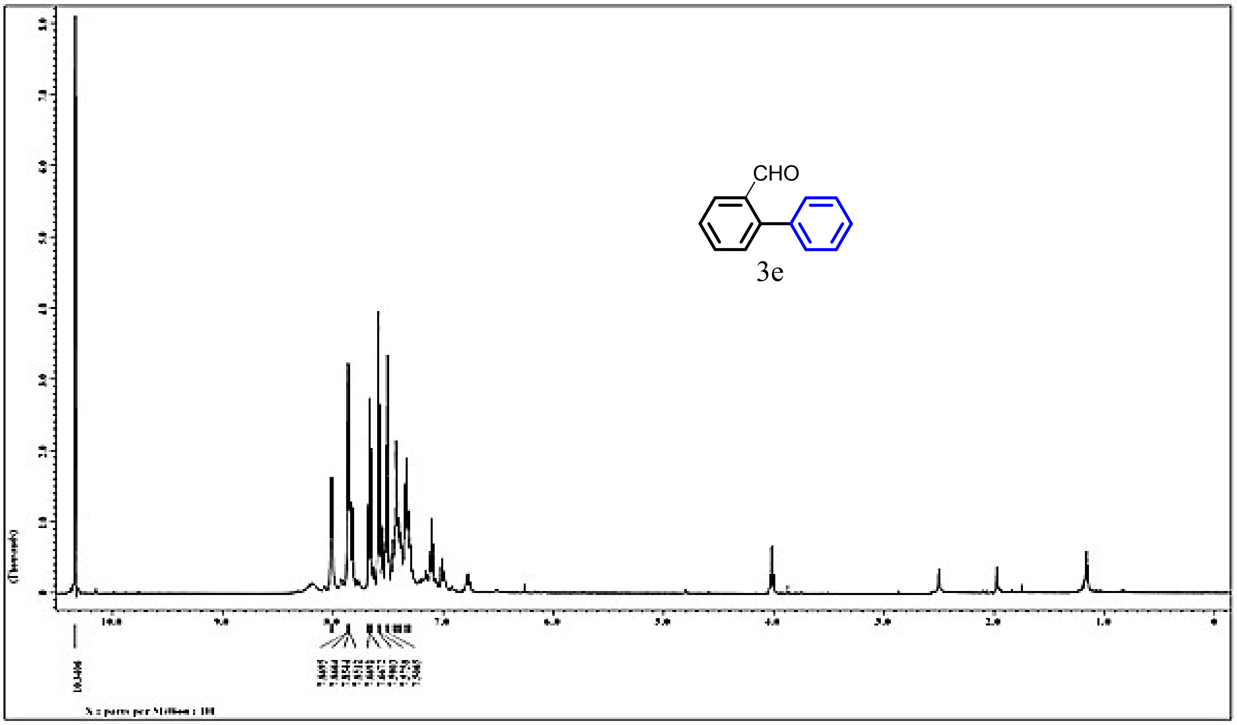

ESI Figure S29: ^{1}HNMR (500 MHz, DMSO-d$_6$. δ$_{ppm}$, 298 K) spectra of Compound **3e.**

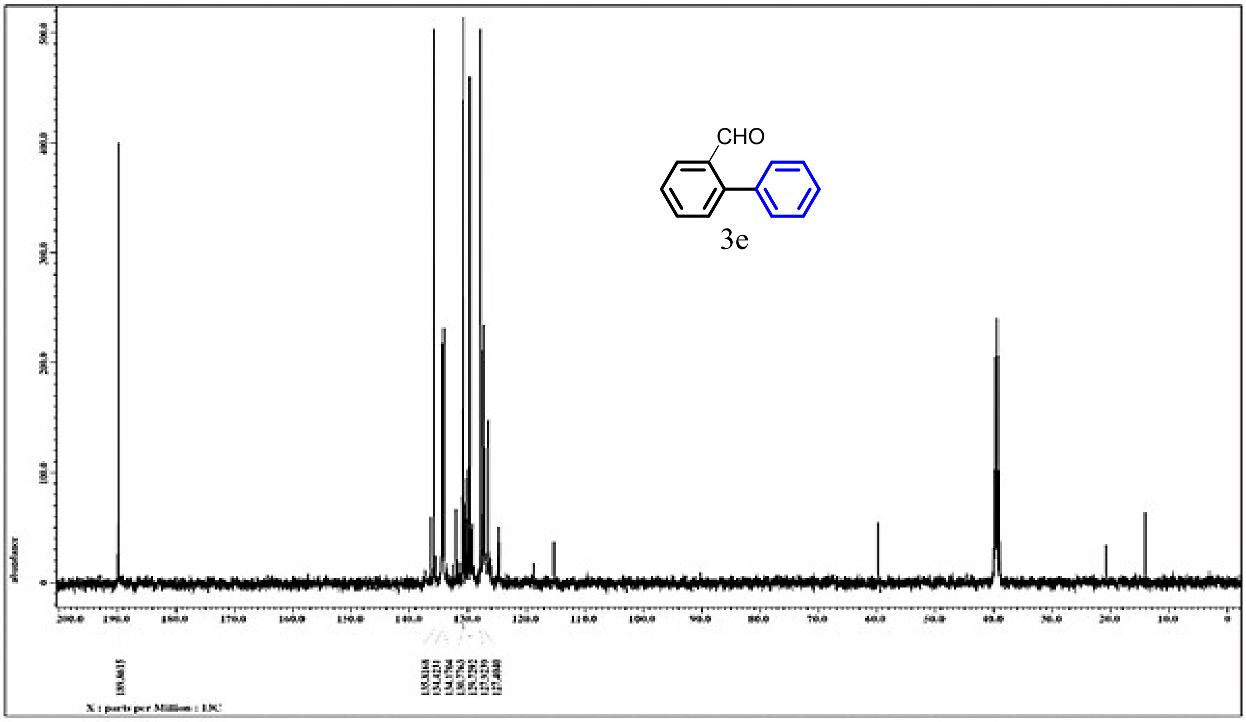

Figure S30: ^{13}CNMR (125 MHz, DMSO-d$_6$. δ$_{ppm}$, 298 K) spectra of Compound **3e.**

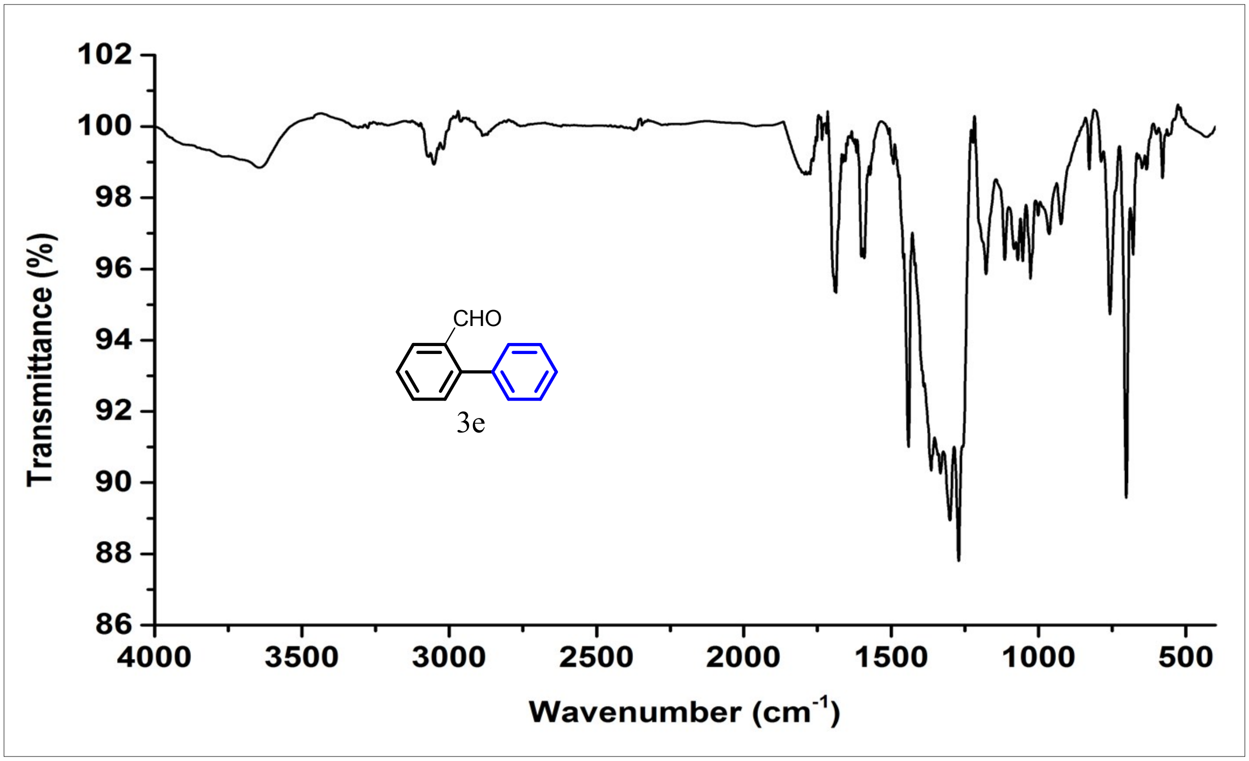

Figure S31: FT-IR Spectra of Compound **3e.**

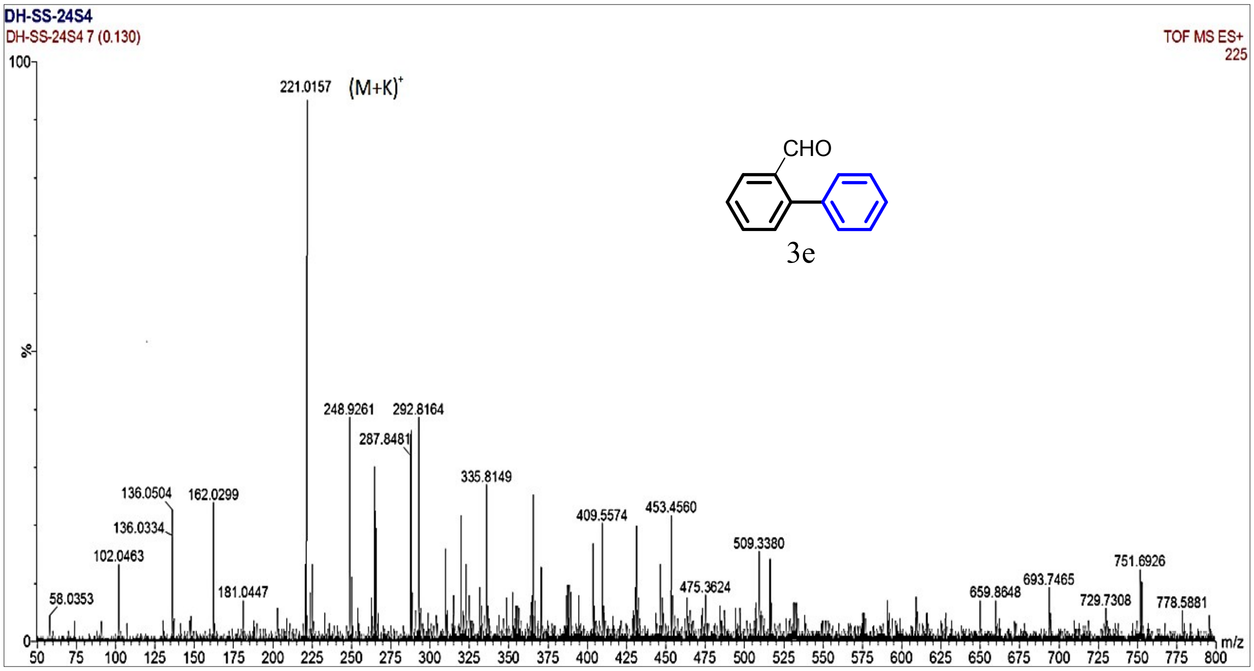

Figure S32: ESI-MS Spectra of Compound **3e.**

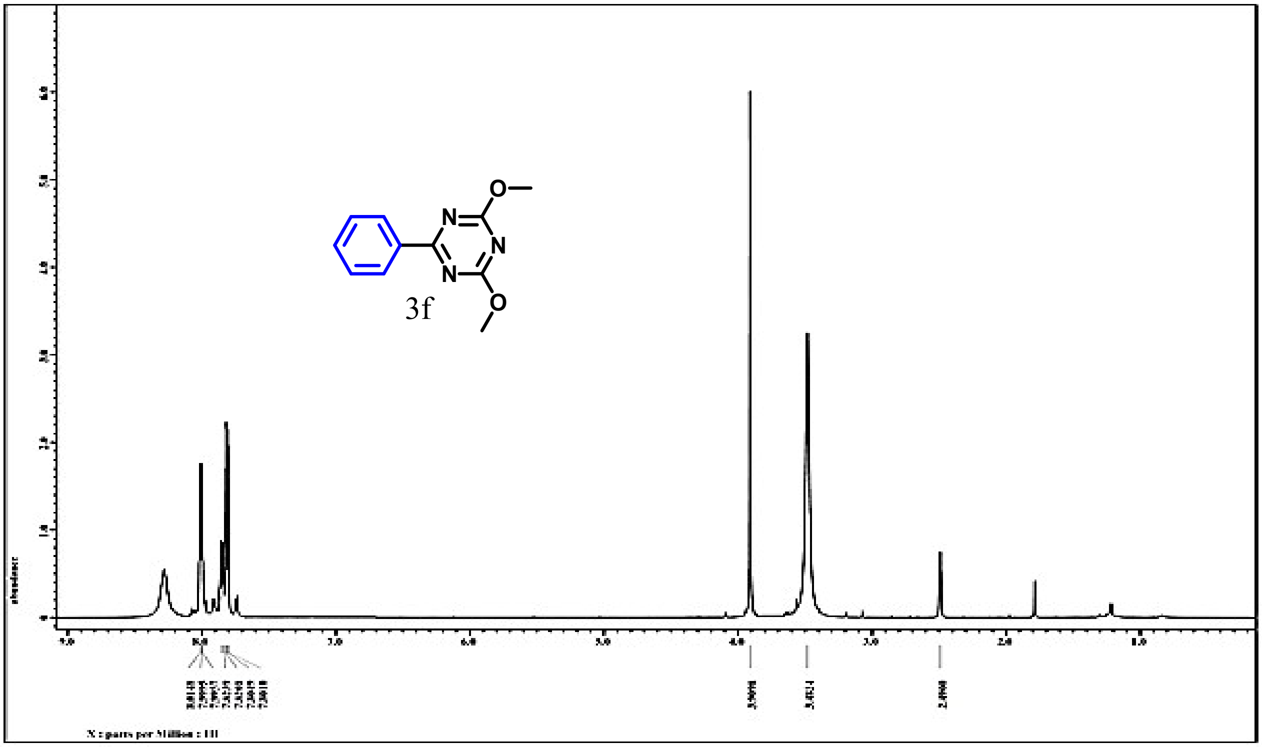

Figure S33: ^{1}HNMR (400 MHz, DMSO-d$_6$. δ_{ppm}, 298 K) spectra of Compound **3f.**

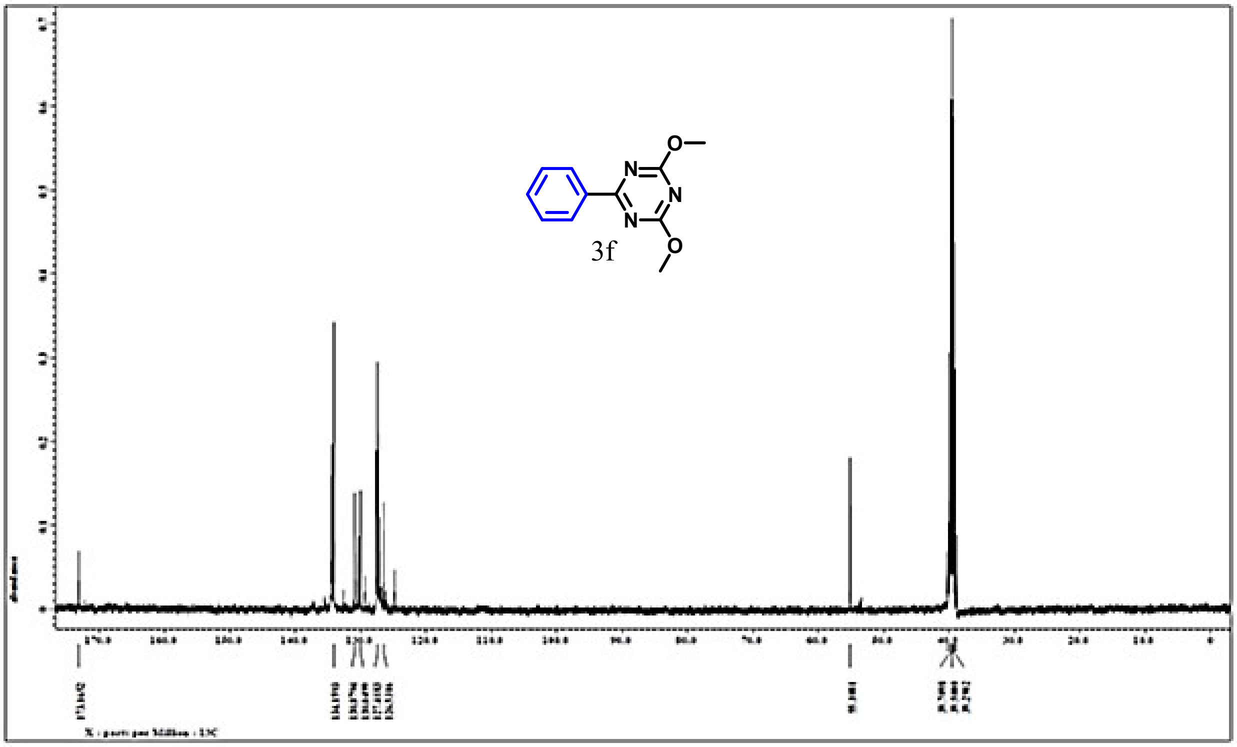

Figure S34: ^{13}CNMR (100 MHz, DMSO-d$_6$. δ_{ppm}, 298 K) spectra of Compound **3f.**

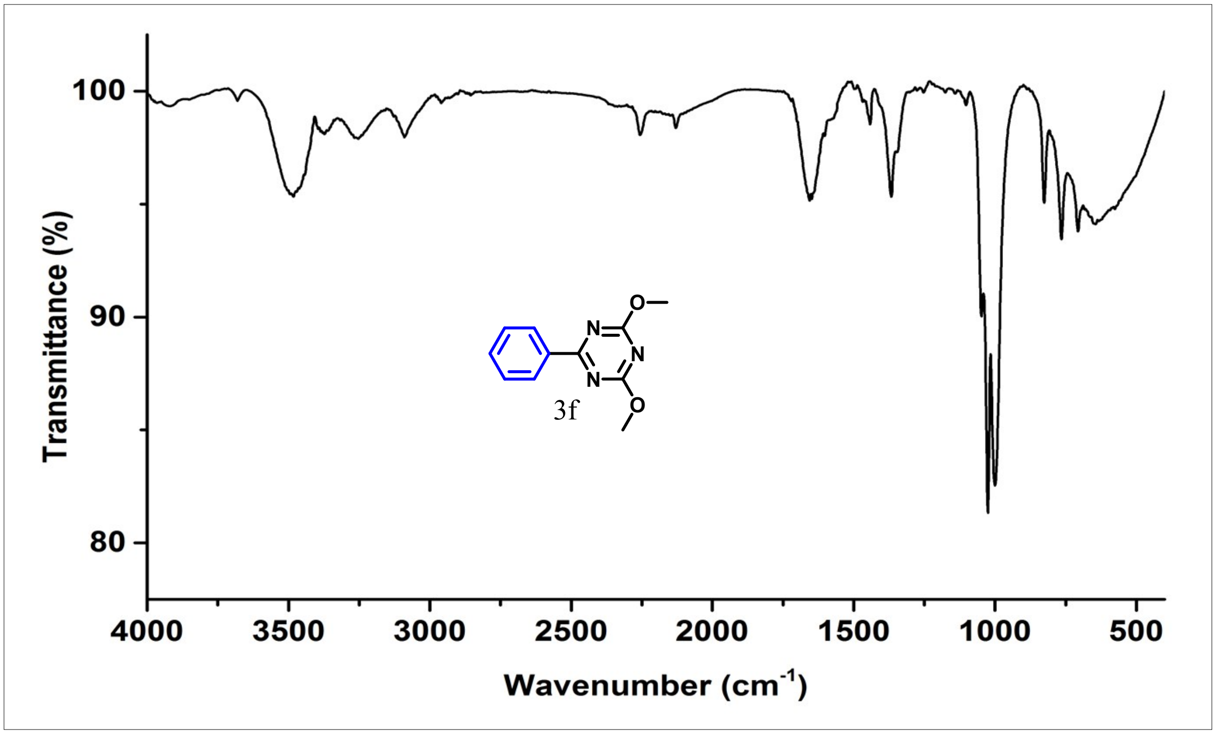

Figure S35: FT-IR Spectra of Compound **3f.**

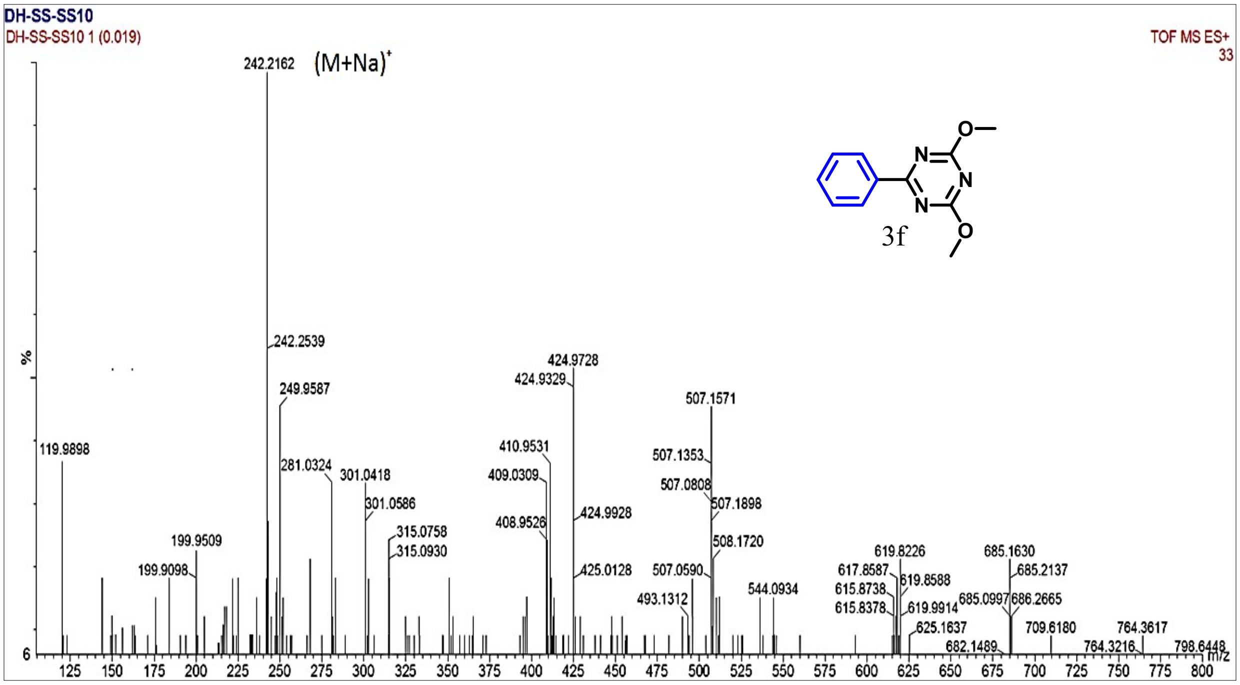

Figure S36: ESI-MS Spectra of Compound **3f.**

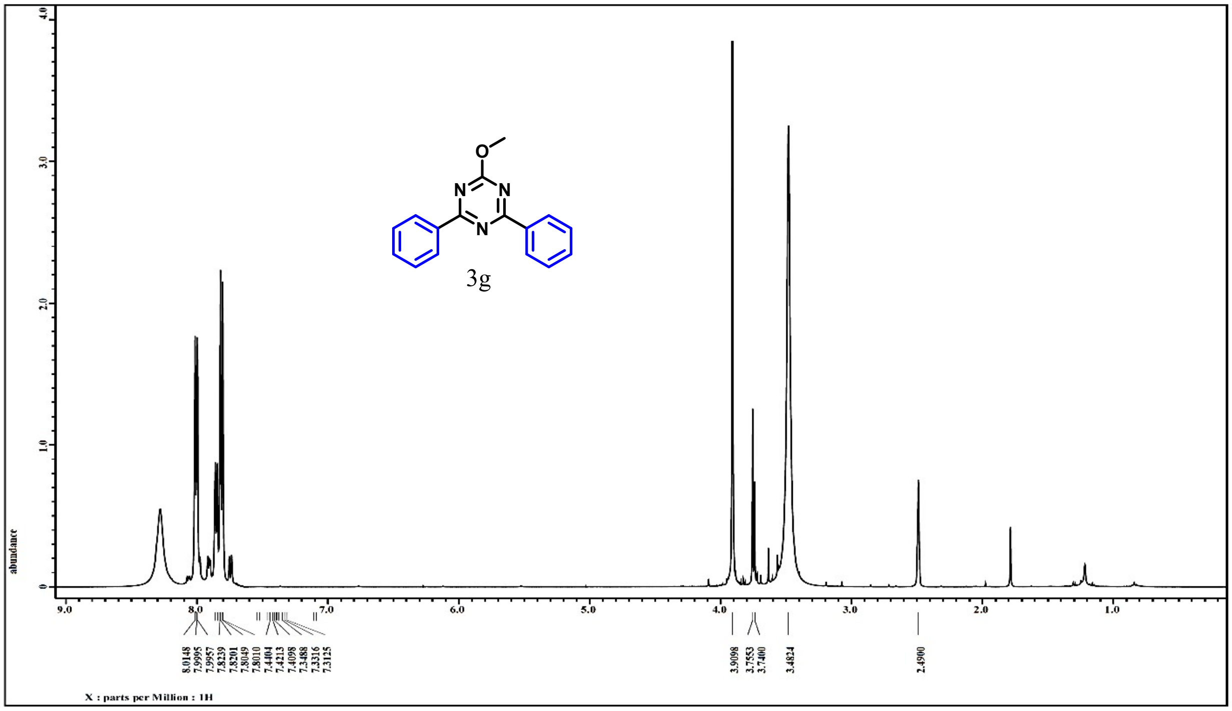

Figure S37: ^{1}HNMR (500 MHz, DMSO-d_6. δ_{ppm}, 298 K) spectra of Compound **3g.**

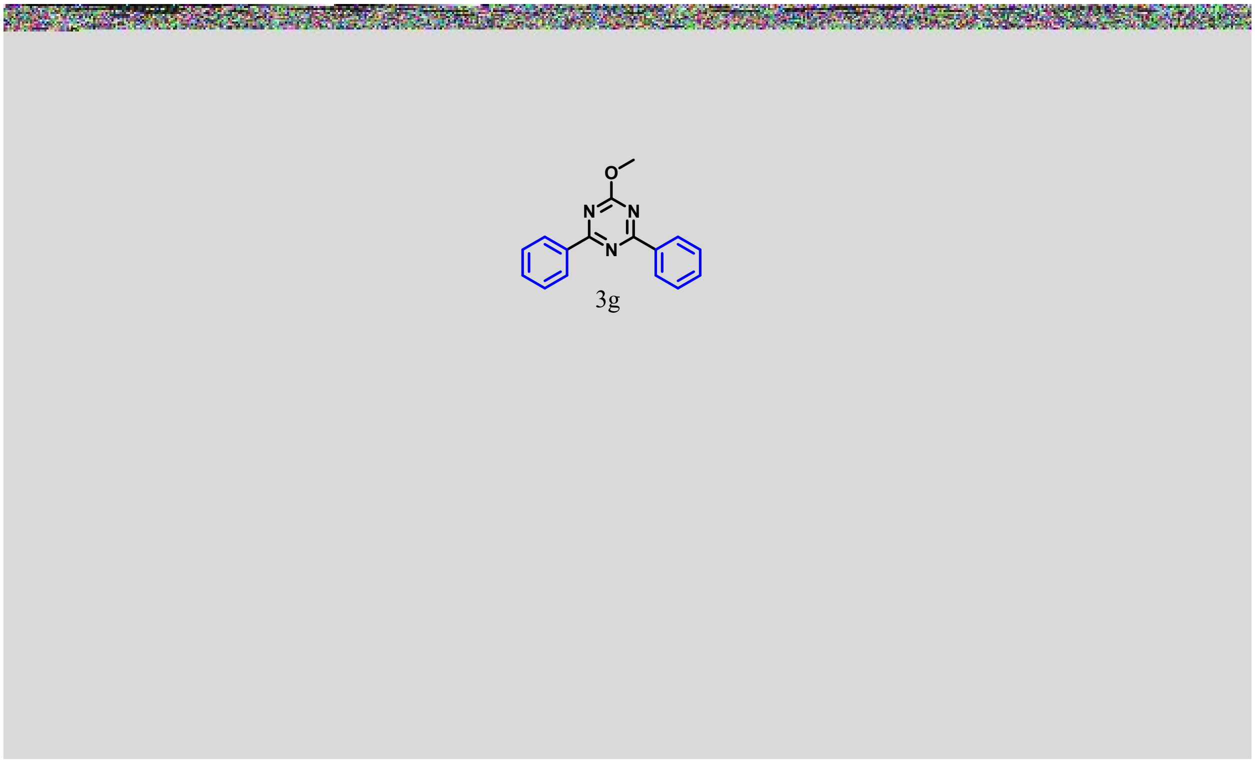

Figure S38: ^{13}CNMR (100 MHz, DMSO-d_6. δ_{ppm}, 298 K) spectra of Compound **3g.**

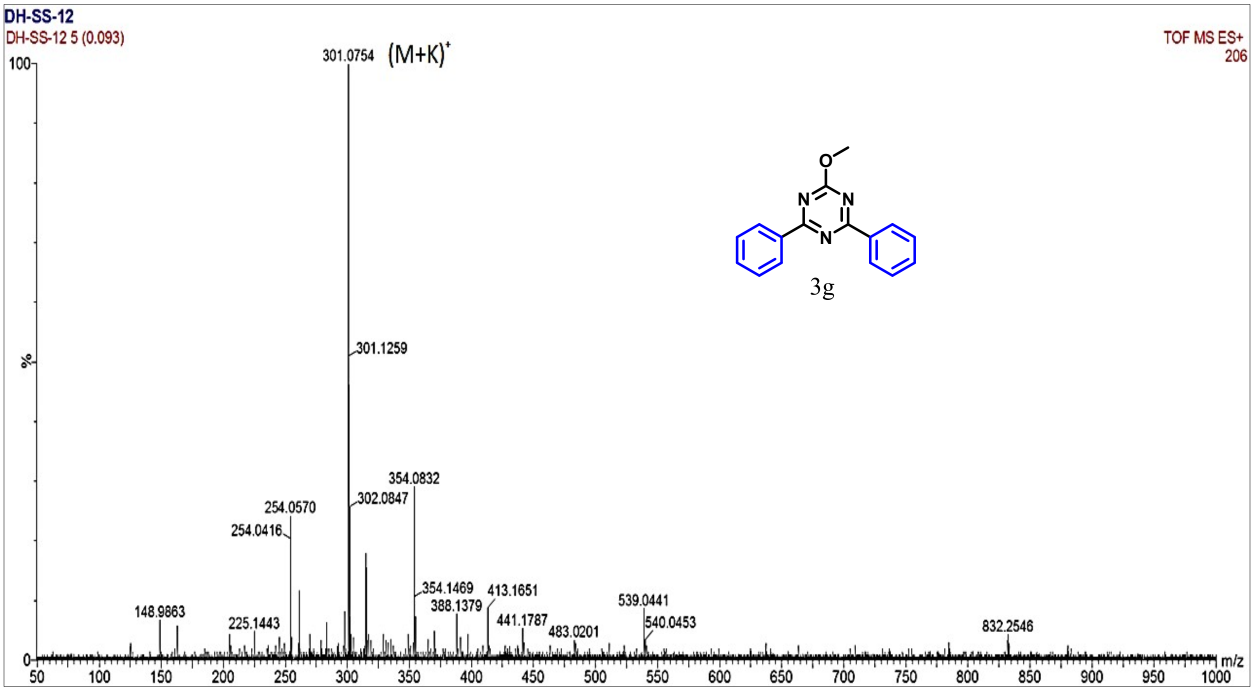

Figure S39: ESI-MS Spectra of Compound **3g.**

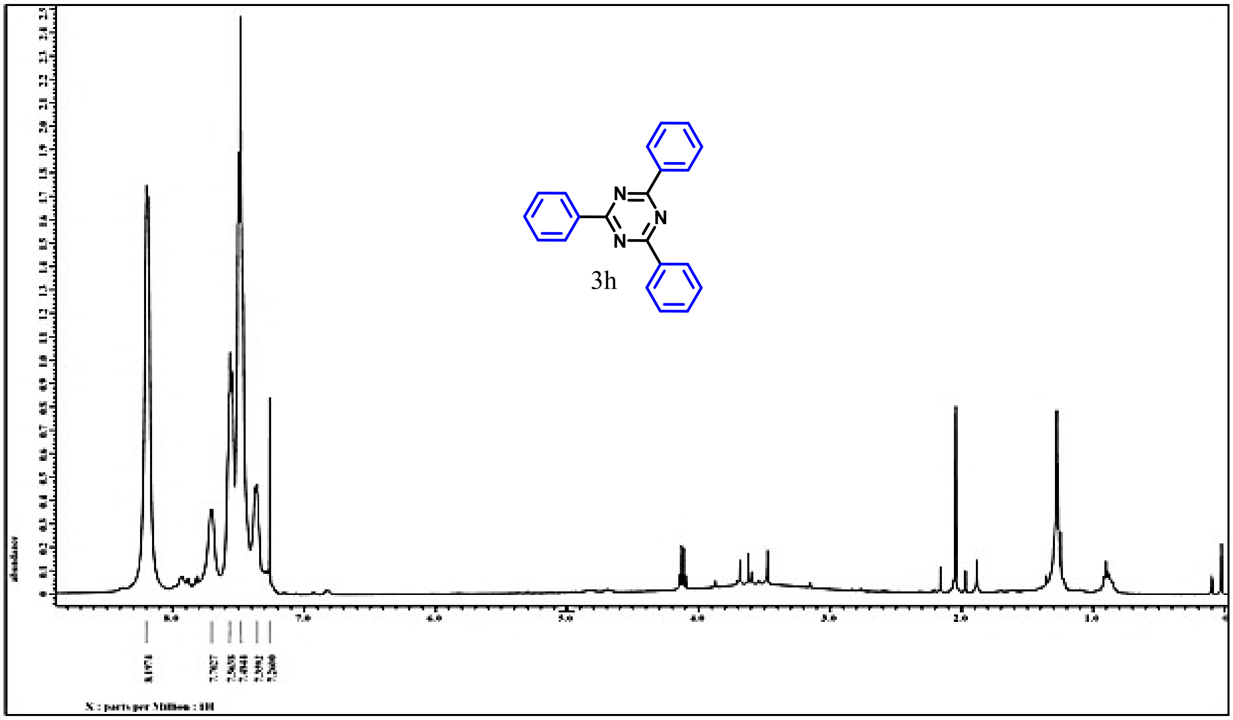

Figure S40: ^{1}HNMR (400 MHz, DMSO-d_6. δ_{ppm}, 298 K) spectra of Compound **3h.**

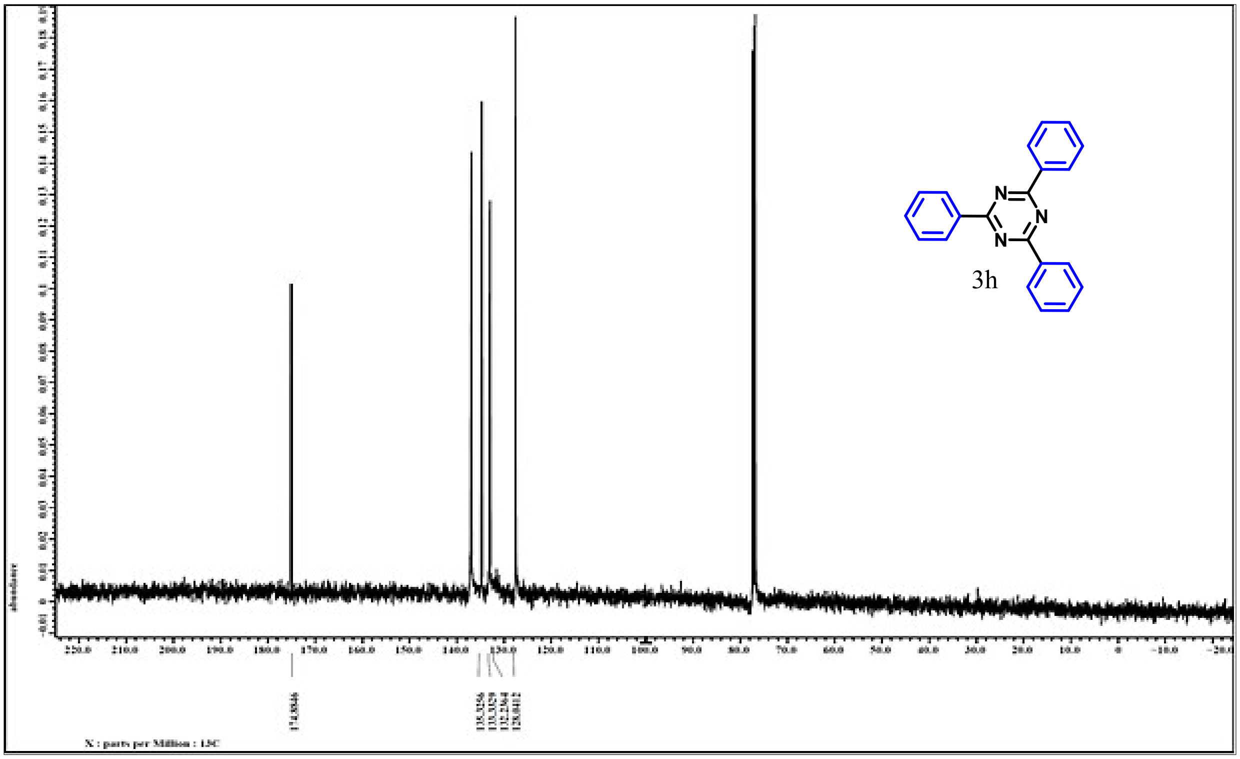

Figure S41: ^{13}CNMR (100 MHz, DMSO-d$_6$. δ_{ppm}, 298 K) spectra of Compound **3h.**

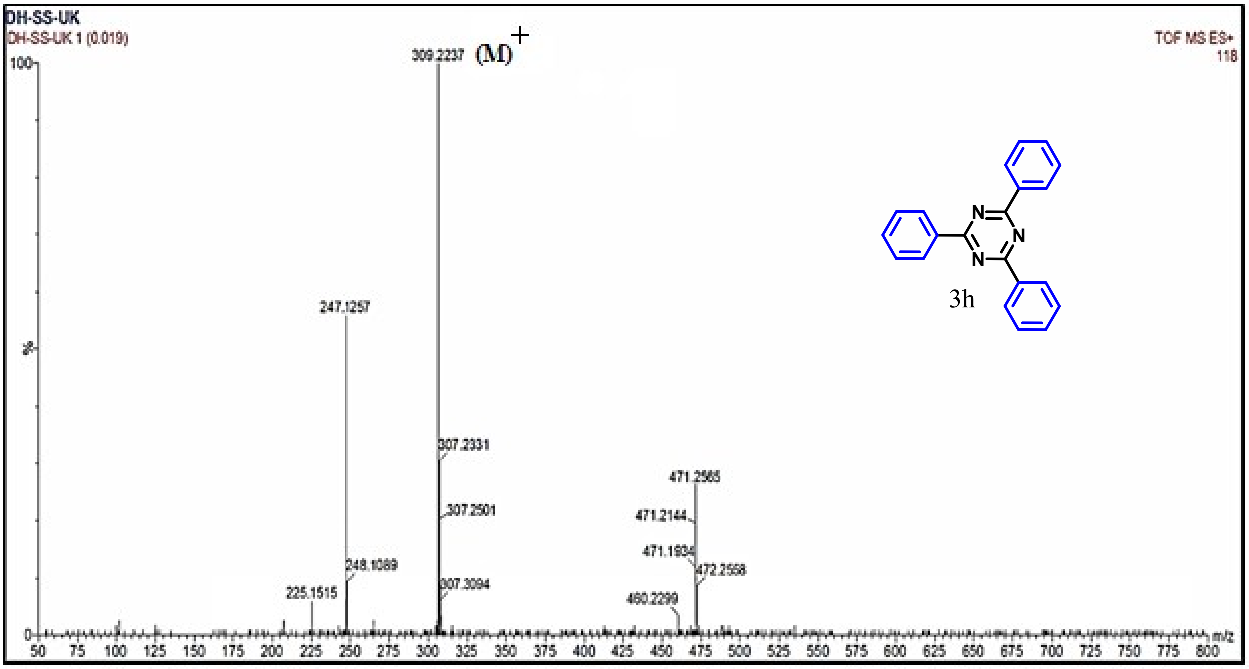

Figure S42: ESI-MS Spectra of Compound **3h.**

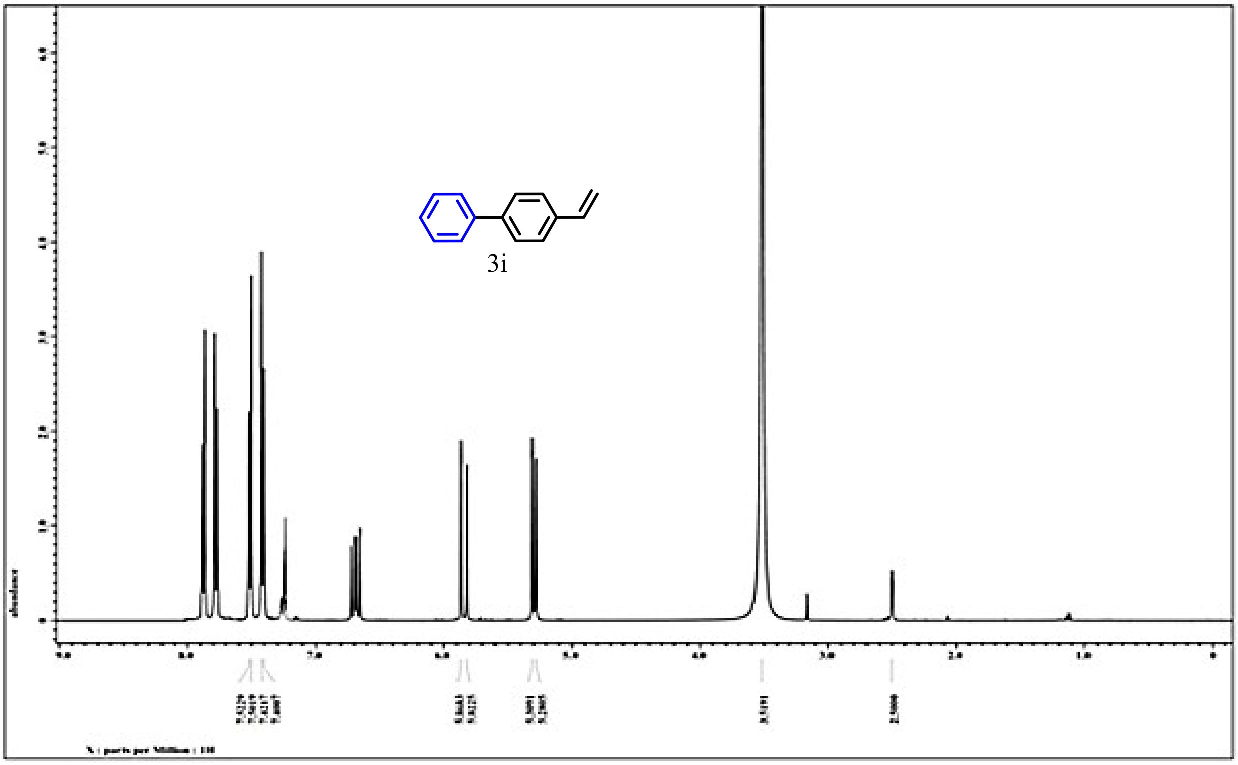

Figure S43: ^{1}HNMR (400 MHz, DMSO-d_6. δ_{ppm}, 298 K) spectra of Compound **3i.**

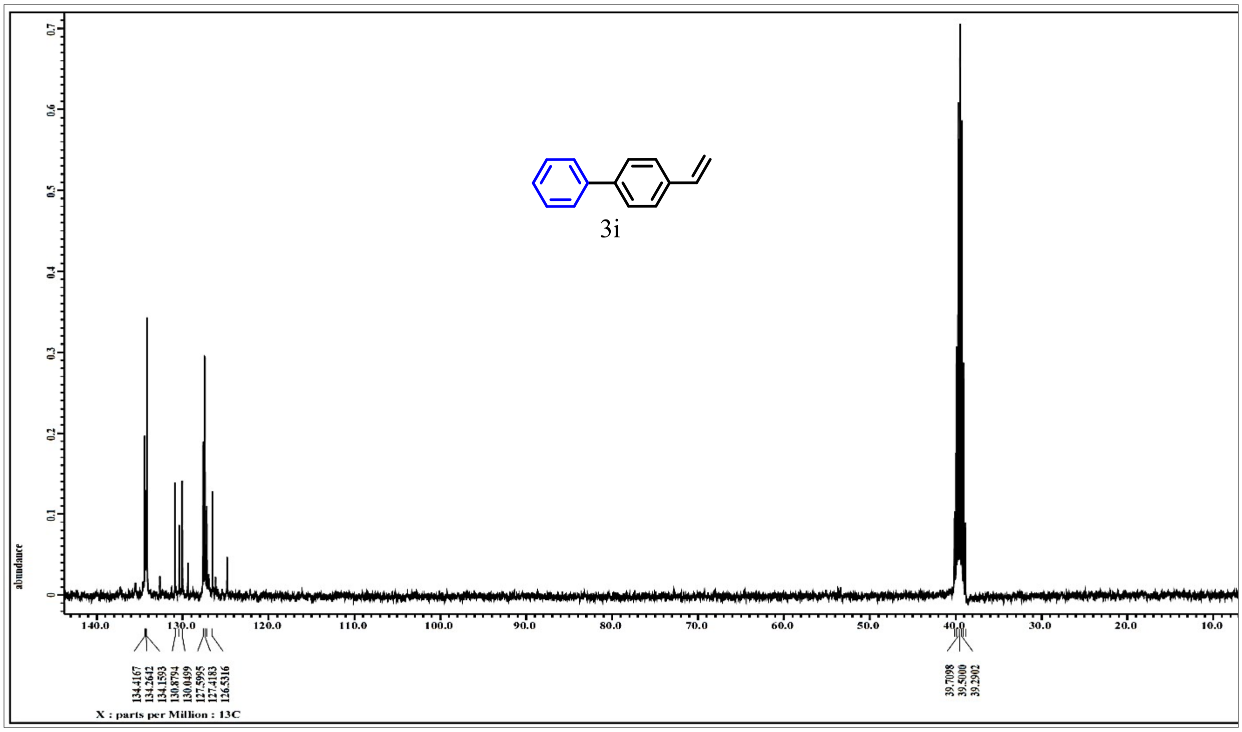

Figure S44: ^{13}CNMR (100 MHz, DMSO-d_6. δ_{ppm}, 298 K) spectra of Compound **3i.**

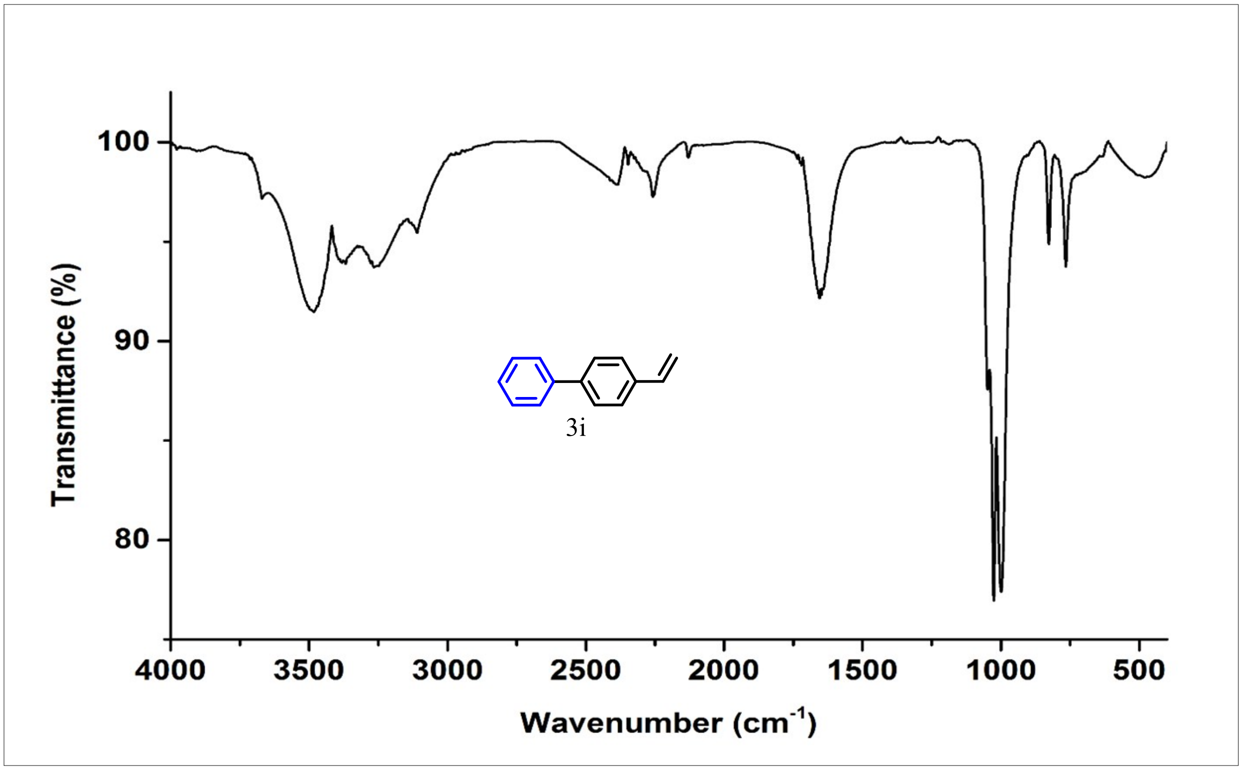

Figure S45: FT-IR Spectra of Compound **3i.**

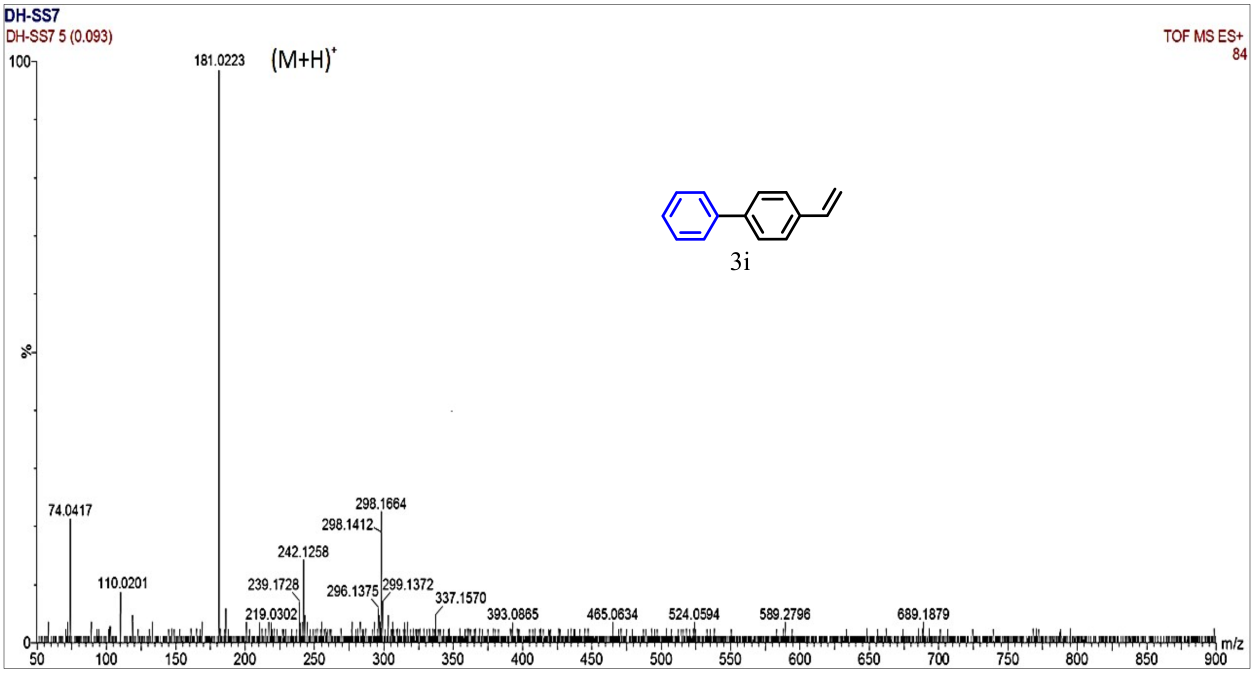

Figure S46: ESI-MS Spectra of Compound **3i.**

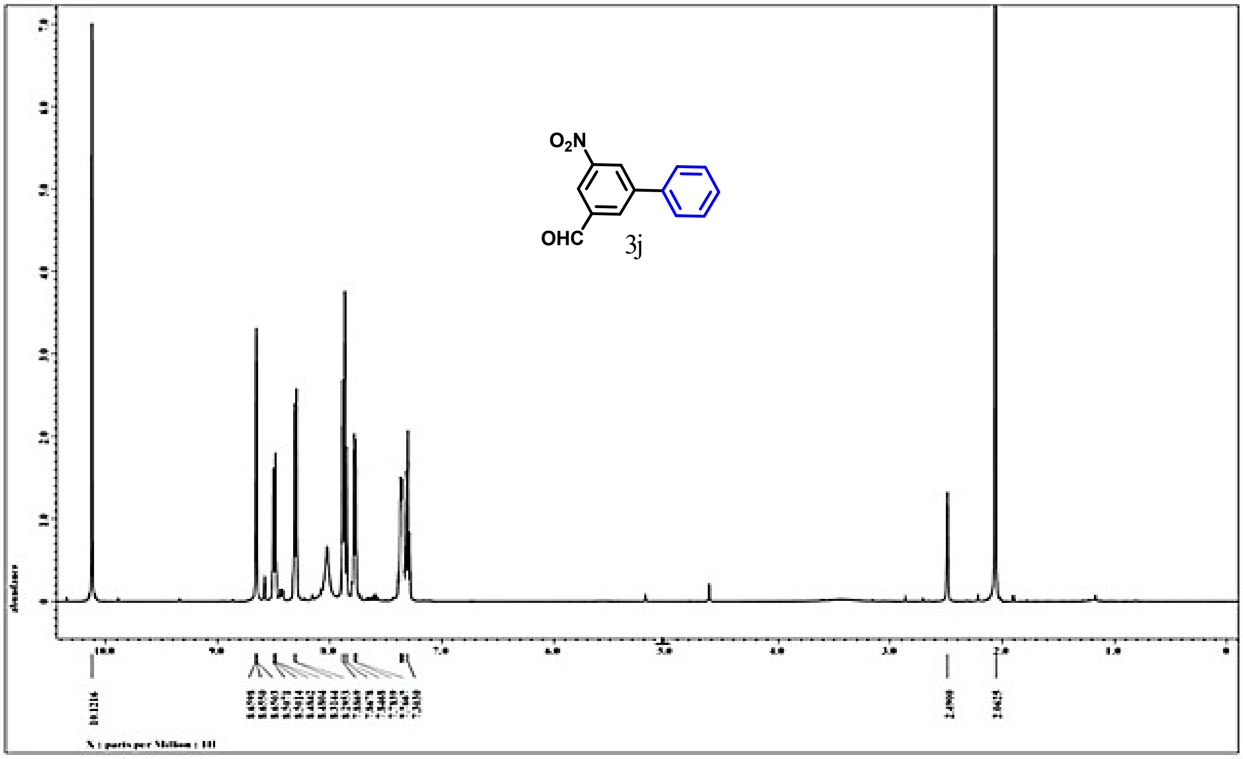

Figure S47: ^{1}HNMR (400 MHz, DMSO-d_6. δ_{ppm}, 298 K) spectra of Compound **3j.**

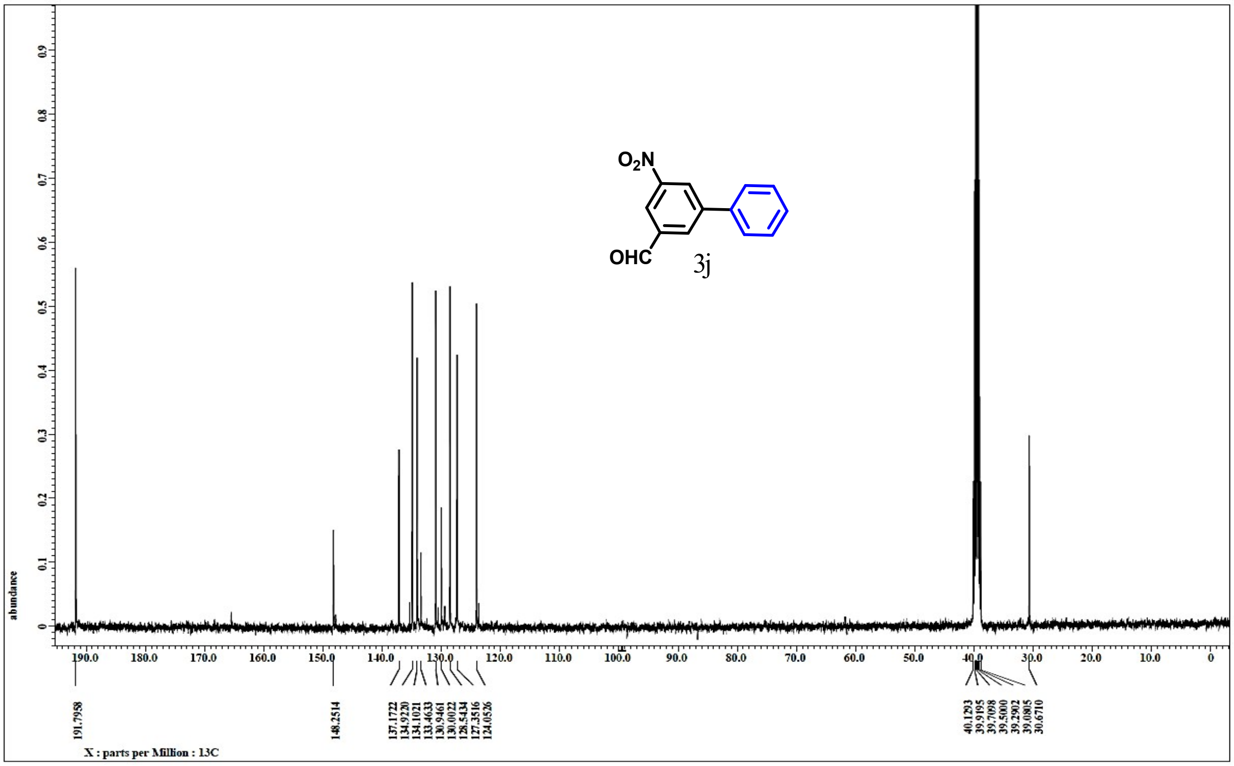

Figure S48: ^{13}CNMR (100 MHz, DMSO-d_6. δ_{ppm}, 298 K) spectra of Compound **3j.**

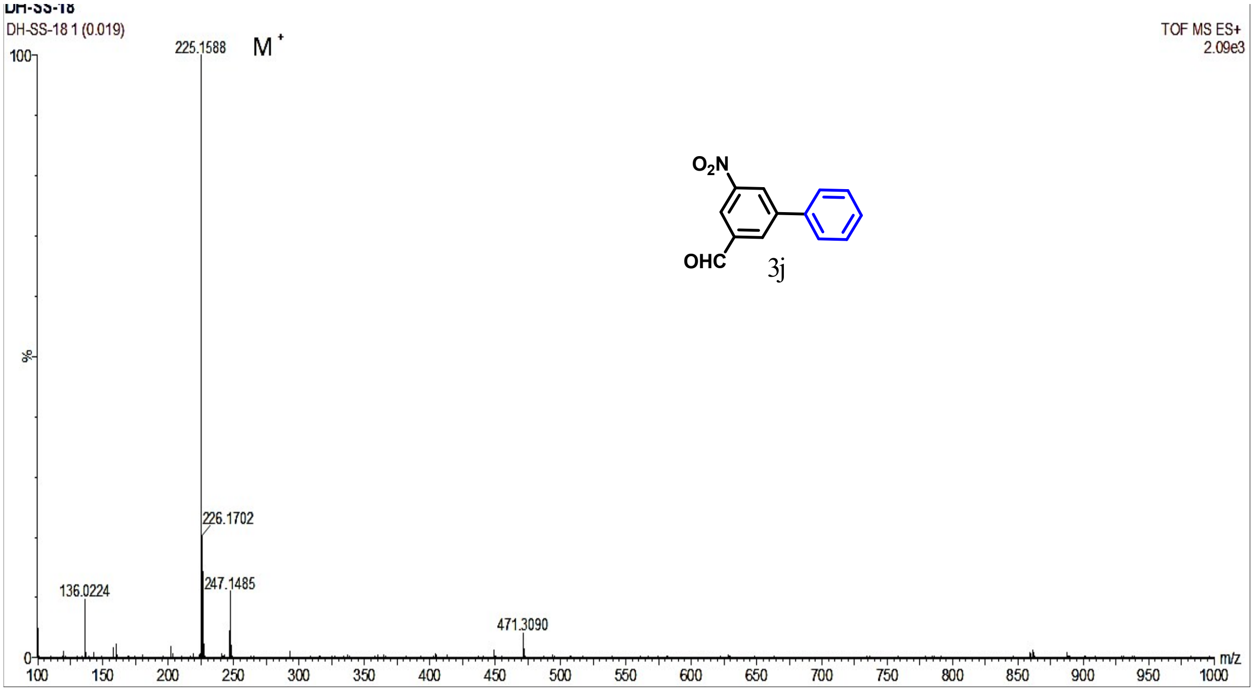

Figure S49: ESI-MS Spectra of Compound **3j.**

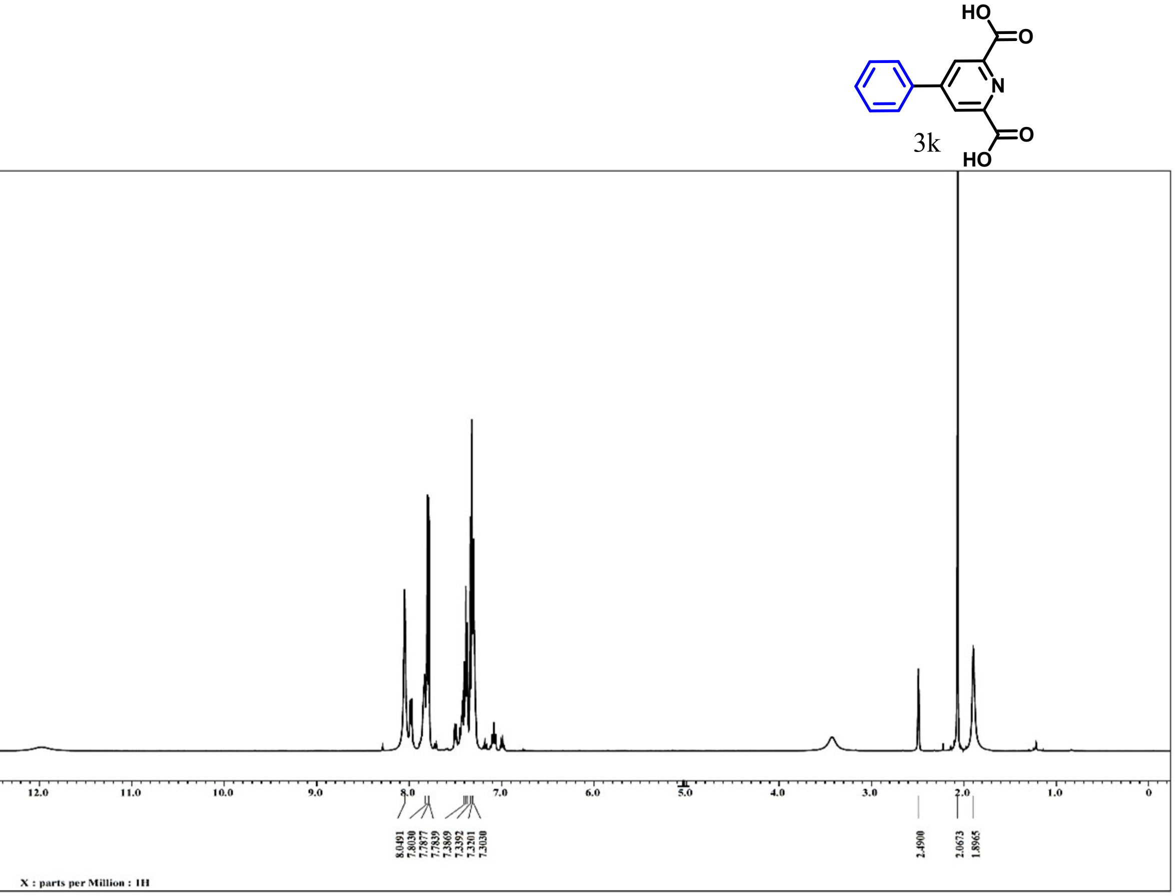

Figure S50: ^{1}HNMR (400 MHz, DMSO-d_6. δ_{ppm}, 298 K) spectra of Compound **3k.**

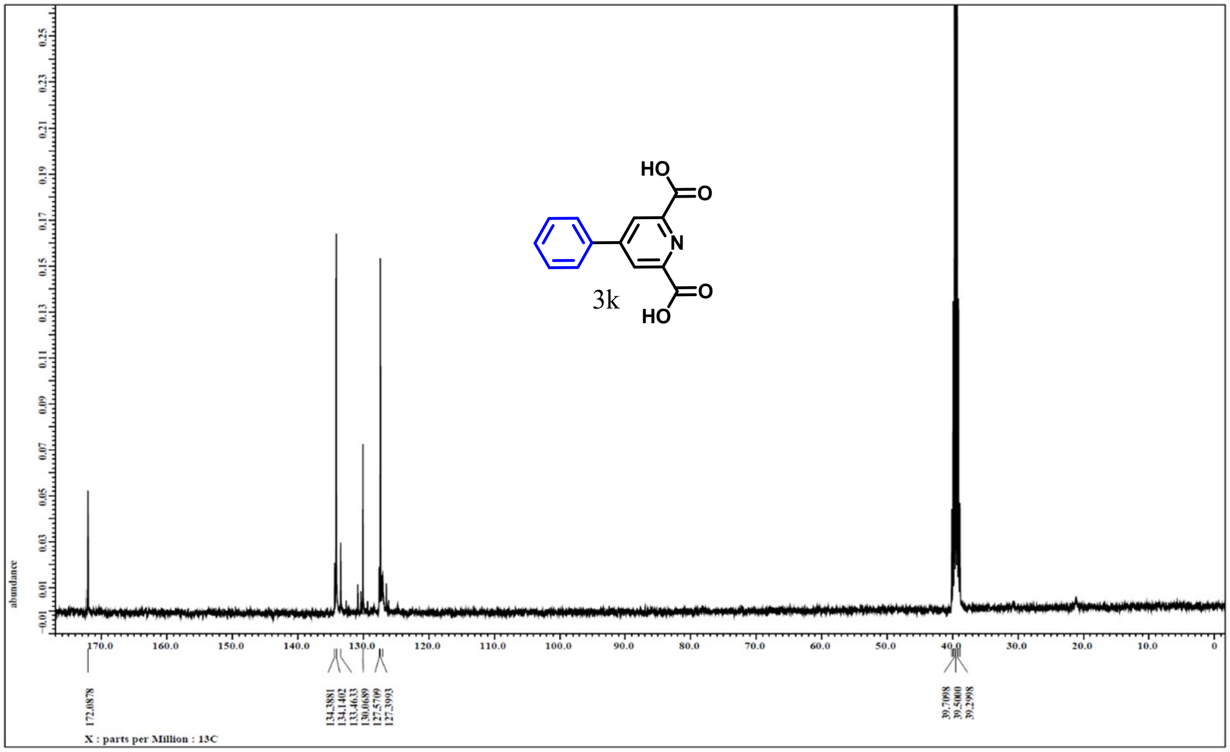

Figure S51: ^{13}CNMR (100 MHz, DMSO-d$_6$. δ_{ppm}, 298 K) spectra of Compound **3k.**

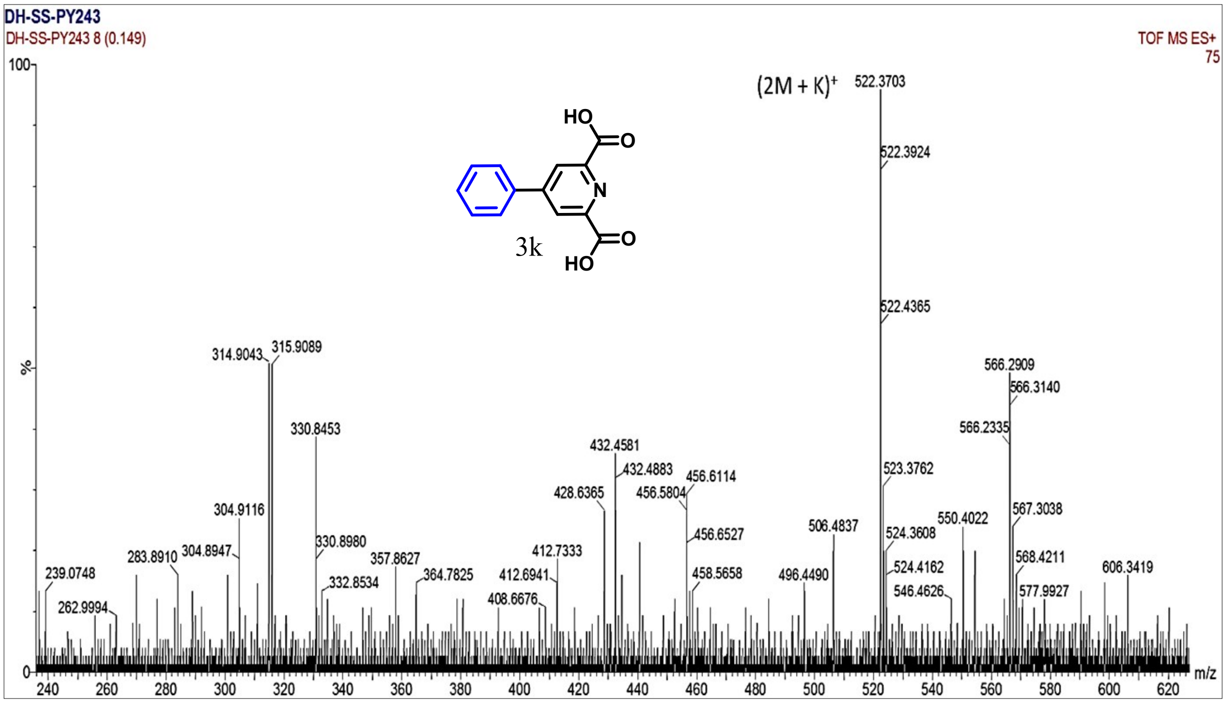

Figure S52: ESI-MS Spectra of Compound **3k.**

ESI-Table 1: Hydrogen bonding parameters in crystal structure of urea modified leucine.

D-H….A	D-H	H…..A	D………A	D-H..A(°)
N(1) --H(1A) ..O(2) a	0.86	2.24	3.1	175
N(2) --H(2) ..O(2) c	0.86	2.27	2.905	131
O(3) --H(3) ..O(1) b	0.82	1.73	2.523	162

Symmetry equivalent: a = 1-x,-1/2+y,3/2-z, b = 1-x,1/2+y,3/2-z, c = -1+x,y,z

Table 2: Torsional angle parameter of urea modified leucine.

	ϕ(°)		ψ(°)
C1-N2-C2-C3	-65.49	N2-C2-C3-O3	-35.91

Table 3: Crystallographic Parameters of Compound **2**.

Empirical formula	$C_7H_{14}N_2O_3$
Formula weight	174.20
Temperature/K	198(140)
Crystal system	Orthorhombic
Space group	$P2_12_12_1$
a/Å	5.2432(9)
b/Å	10.3449(19)
c/Å	17.386(4)
α/°	90
β/°	90
γ/°	90
Volume/Å^3	943.0(3)
Z	4
ρ_{calc}g/cm^3	1.227
μ/mm^{-1}	0.096
F(000)	376.0

Crystal size/mm^3	0.2589 × 0.2489 × 0.2209
Radiation	MoKα (λ = 0.71073)
2Θ range for data collection/°	4.582 to 50.014
Index ranges	-6 ≤ h ≤ 6, -12 ≤ k ≤ 12, -17 ≤ l ≤ 20
Reflections collected	3782
Independent reflections	1659 [R_{int} = 0.0788, R_{sigma} = 0.0906]
Data/restraints/parameters	1659/0/113
Goodness-of-fit on F^2	1.073
Final R indexes [I>=2σ (I)]	R_1 = 0.0676, wR_2 = 0.1787
Final R indexes [all data]	R_1 = 0.0774, wR_2 = 0.1915
Largest diff. peak/hole / e Å^{-3}	0.40/-0.43
Flack parameter	-2.7(10)

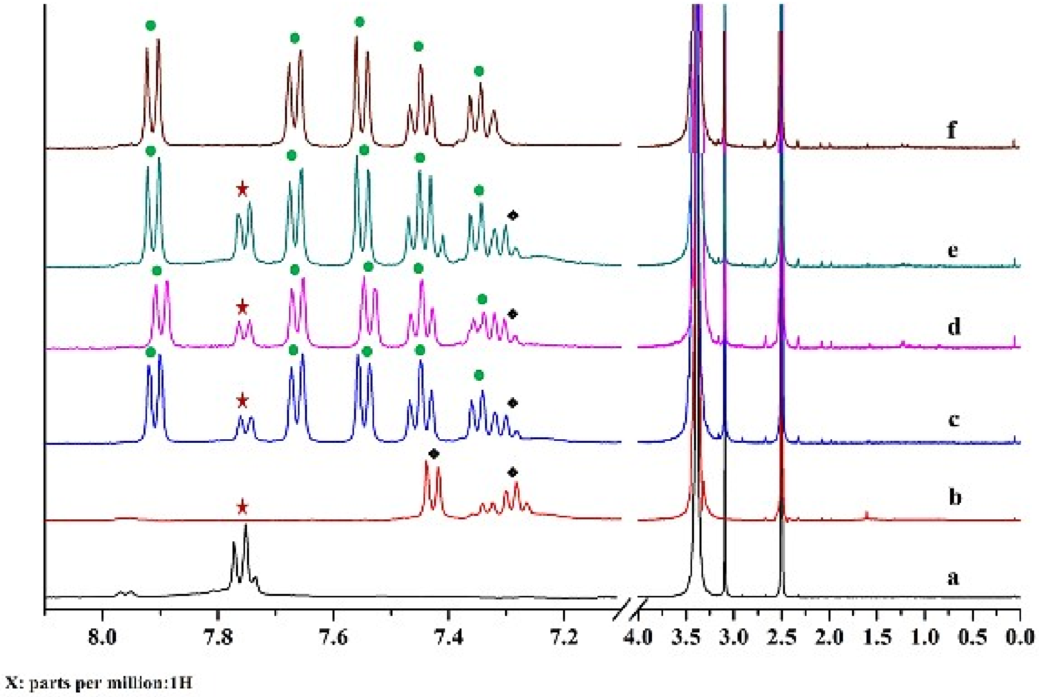

Fig. S53:^{1}HNMR Spectra of a) 4-Bromo Benzoic Acid, b) Phenyl Boronic Acid, c) After 1st catalytic Cycle, d) After 2nd catalytic Cycle, e) After 3rd catalytic Cycle, f) compound **5**.

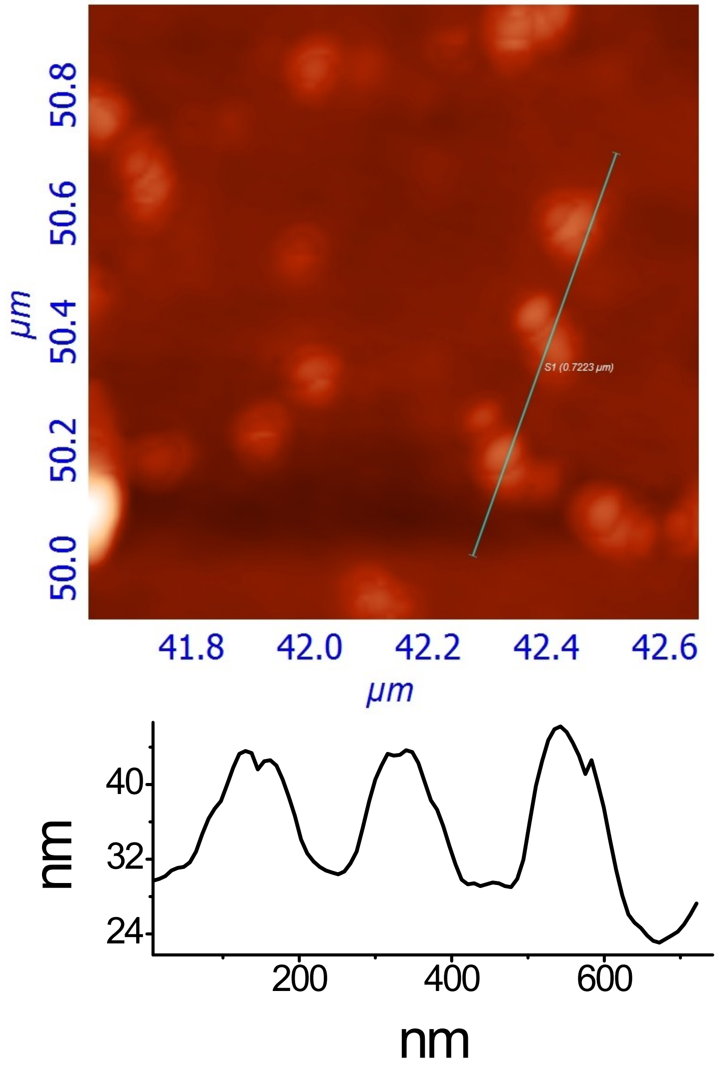

Figure S54: AFM image and height profile diagram of the gold nanoparticles.
